# Fentanyl Family at the Mu-Opioid Receptor: Uniform Assessment of Binding and Computational Analysis

**DOI:** 10.3390/molecules24040740

**Published:** 2019-02-19

**Authors:** Piotr F. J. Lipiński, Piotr Kosson, Joanna Matalińska, Piotr Roszkowski, Zbigniew Czarnocki, Małgorzata Jarończyk, Aleksandra Misicka, Jan Cz. Dobrowolski, Joanna Sadlej

**Affiliations:** 1 Department of Neuropeptides, Mossakowski Medical Research Centre, Polish Academy of Sciences, 02-106 Warsaw, Poland; jmatalinska@imdik.pan.pl (J.M.); misicka@imdik.pan.pl (A.M.); 2 Toxicology Research Laboratory, Mossakowski Medical Research Centre, Polish Academy of Sciences, 02-106 Warsaw, Poland; pkosson@imdik.pan.pl; 3 Faculty of Chemistry, University of Warsaw, 02-093 Warsaw, Poland; roszkowski@chem.uw.edu.pl (P.R.); czarnoz@chem.uw.edu.pl (Z.C.); misicka@chem.uw.edu.pl (A.M.); 4 National Medicines Institute, 00-725 Warsaw, Poland; m.jaronczyk@nil.gov.pl (M.J.); j.dobrowolski@nil.gov.pl (J.Cz.D.); 5 Faculty of Mathematics and Natural Sciences, University of Cardinal Stefan Wyszyński, 1/3 Wóycickiego-Str., 01-938 Warsaw, Poland

**Keywords:** fentanyl, opioid receptors, molecular dynamics, designer drugs

## Abstract

Interactions of 21 fentanyl derivatives with μ-opioid receptor (μOR) were studied using experimental and theoretical methods. Their binding to μOR was assessed with radioligand competitive binding assay. A uniform set of binding affinity data contains values for two novel and one previously uncharacterized derivative. The data confirms trends known so far and thanks to their uniformity, they facilitate further comparisons. In order to provide structural hypotheses explaining the experimental affinities, the complexes of the studied derivatives with μOR were modeled and subject to molecular dynamics simulations. Five common General Features (**GFs**) of fentanyls’ binding modes stemmed from these simulations. They include: **GF1**) the ionic interaction between D147 and the ligands’ piperidine NH^+^ moiety; **GF2**) the N-chain orientation towards the μOR interior; **GF3**) the other pole of ligands is directed towards the receptor outlet; **GF4**) the aromatic anilide ring penetrates the subpocket formed by TM3, TM4, ECL1 and ECL2; **GF5**) the 4-axial substituent (if present) is directed towards W318. Except for the ionic interaction with D147, the majority of fentanyl-μOR contacts is hydrophobic. Interestingly, it was possible to find nonlinear relationships between the binding affinity and the volume of the N-chain and/or anilide’s aromatic ring. This kind of relationships is consistent with the apolar character of interactions involved in ligand–receptor binding. The affinity reaches the optimum for medium size while it decreases for both large and small substituents. Additionally, a linear correlation between the volumes and the average dihedral angles of W293 and W133 was revealed by the molecular dynamics study. This seems particularly important, as the W293 residue is involved in the activation processes. Further, the Y326 (OH) and D147 (Cγ) distance found in the simulations also depends on the ligands’ size. In contrast, neither RMSF measures nor D114/Y336 hydrations show significant structure-based correlations. They also do not differentiate studied fentanyl derivatives. Eventually, none of 14 popular scoring functions yielded a significant correlation between the predicted and observed affinity data (R < 0.30, *n* = 28).

## 1. Introduction

Fentanyl (*N*-phenyl-*N*-[1-(2-phenylethyl)piperidin-4-yl]propanamide) is a very strong agonist of μ-opioid receptor (μOR) and as such it is one of the most important analgesics used in medicine. Being highly lipophilic, it is 50–125 times more potent than morphine and it has a faster onset of action [1]. The compound’s applications include management of chronic and cancer pain as well as postoperative pain and analgosedation in intensive care settings. Further, the substance is used for surgery premedication or general anesthesia (or adjunct to general anesthesia) before minor and major surgical procedures. 

The compound is a prototype of a wider family of synthetic opioid analgesics: 4-anilidopiperidines. Since the early 1960s, when fentanyl was invented by Paul Janssen and co-workers [2], numerous members of the group have been synthesized and reported. This research played a key role in developing our knowledge on structure–activity relationships (SAR) for opioid analgesics. It resulted in a bunch of very interesting and useful compounds [3], including extremely potent derivatives such as ohmefentanyl and carfentanil, or ultra-short acting opioids as remifentanil or alfentanil. A very comprehensive review from the point of view of SAR and chemistry of the class was provided by Vardanyan and Hruby [3]. Further interesting accounts can be found in other recent papers [2,4]. 

Even though fentanyl and its derivatives have been known for now almost 60 years, the compound is still able to stimulate modern research in many subfields at the interface of chemistry and pharmacology. For instance, Valdez et al. presented optimized syntheses of fentanyl and analogues [5] and Jevtić et al. described a route for new derivatives via orthogonally protected (in positions 1 and 3) 3-amino-4-anilidopiperidines [6]. Issues of formulation and drug delivery prompted several groups to study fentanyl interactions with cyclodextrins in aqueous solutions [7] or solid state interactions of fentanyl citrate polymorphs and solvates [8]. In a very interesting contribution, Bick et al. described design and synthesis of proteins binding fentanyl for purposes of creating environmental sensors [9].

Yet novel fentanyl derivatives continue to appear. Researchers of various groups reported novel analogues, including those of norsufentanil [10], carfentanil amides [11] or acrylic derivatives [12]. Fentanyl scaffold has also been used in the design of bivalent (multitarget) compounds to create mixed μ-/δ-OR ligands [13,14,15], or molecules joining even more distant pharmacophores like opioid receptor agonist and FAAH/MAGL hydrolases inhibitors [16], or opioid and dopamine receptors D2/D3 ligands [17]. Most excitingly, fentanyl has its place also in the most up-to-date trends of pain pharmacology that is in the research on biased μOR agonists which holds promise for finding strong analgesics without adverse effects. Although fentanyl itself or sufentanil are able to very effectively elicit unwanted (connected to adverse effects) β-arrestin recruitment [18], structurally close lie compounds with much higher preference for G-protein activation [19]. Another fascinating possibility for developing safer analgesic agents opens up with pH-dependent opioid agonists. Here, novel, computationally designed, fluorinated fentanyl derivatives showed injury-restricted analgesia in rat models of inflammatory, postoperative or neuropathic pain [20,21,22], and for some of them, a significant reduction of typical opioid adverse effects was reported [22]. For now, even the long-known derivatives still have potential for therapeutic innovation, as testified to by a recent (2018) FDA approval of sufentanil sublingual formulation (Dsuvia^TM^ by AcelRx Pharmaceuticals Inc., Redwood, CA, USA) for the treatment of acute pain [23]. 

Unfortunately, the excellent analgesics and research compounds of the fentanyl family have their darker side, too. The substances bear a high potential for abuse and addiction, and because of narrow difference between the active (be it for medical or abuse purposes) dose and the lethal one, they can easily deprive an abuser of life. The issue has been a public health concern since as long ago as late 1970s, when α-methylfentanyl was part of the so-called ‘China White’ responsible for many hundred deaths of street drug users. In recent years the toxicological significance of fentanyl has grown even more. The United States is amid the opioid overdose crisis. According to the estimates by National Centre For Health Statistics, US, in 2017 [24], more than two thirds of drug overdose deaths were caused by opioids (49068 out of 72306), and among them these resulting from illicitly manufactured fentanyl (or fentanyl derivatives) intake were the largest group (more than 50%) and the most sharply growing one. Some alarming reports come also from European countries [25]. The reasons for this deplorable popularity renaissance include relatively facile and cheap syntheses as well as high potencies of fentanyl derivatives. From the point of view of chemical sciences, the fentanyl crisis increases the demand for analytical capabilities to identify illicit substances [26,27,28,29,30,31,32,33,34], in particular novel derivatives. This is due to not only the requirements of forensic medicine, but it is also important for regulatory, preventive and, law enforcement purposes. Here, knowing the current abuse trends enables taking proper countermeasures in a time-efficient manner.

**Figure 1:**
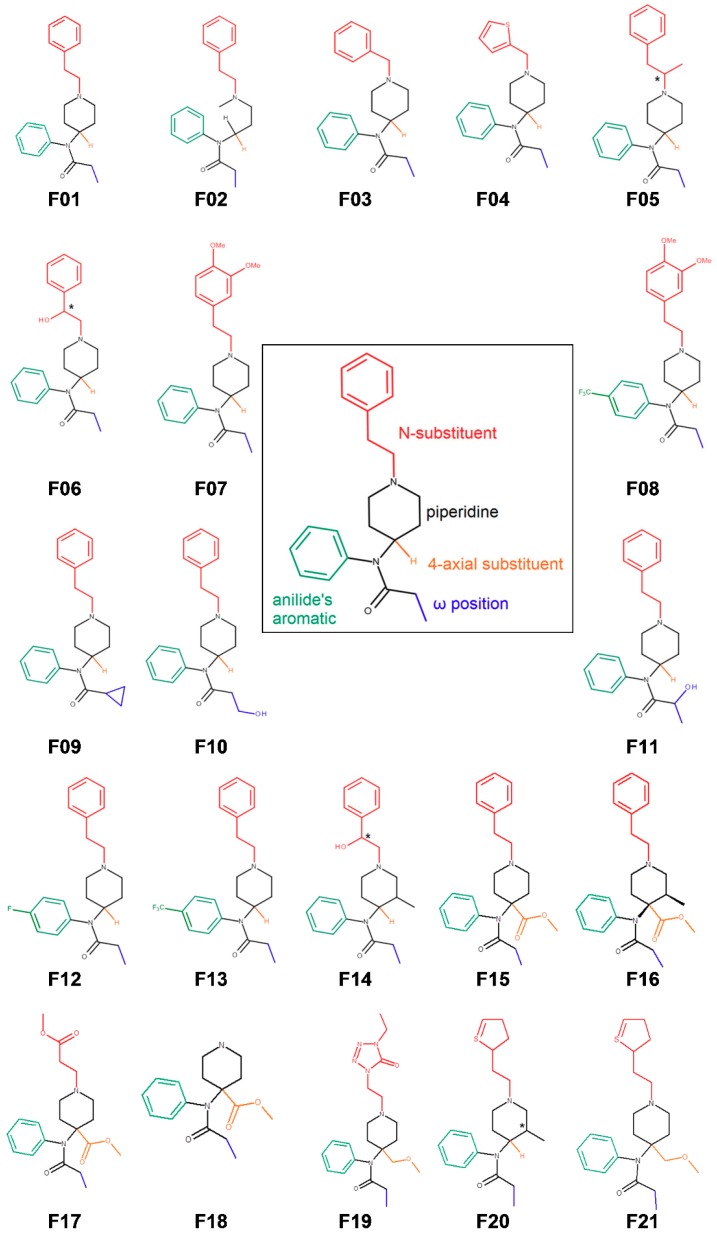
Structures of 21 fentanyl derivatives under the study and a logical disassembly of the parent fentanyl into structural elements subject to variation in the set.

Having regard to the importance of the fentanyl family in both medicinal and forensic chemistry, we have set out to study the interactions of 21 fentanyl derivatives (Figure 1) with μ-opioid receptor by experiment and computations. Our aims were:

1. to provide the readers with a uniform assessment (ranking) of the affinity of fentanyl derivatives for the receptor,
2. to translate the observed experimental trends in structure–activity relationships into structural terms by the means of molecular modeling, and
3. using our experimental values, to see whether the popular scoring methods would be able to reproduce them with reasonable accuracy.

The molecular modeling protocol used here included determination of the binding pose, as well as the confirmation of its stability by the means of molecular dynamics (MD), and finally assessing the interaction energies by scoring functions. Molecular dynamics simulations allow for studying dynamic behavior of molecules over time in atomic detail. This pertains also to biomolecules and their complexes. Thus MD can be likened to a ‘computational microscope’ enabling to reliably peek at key biochemical processes such as protein folding, membrane transport or the conformational changes critical to protein function [35,36]. MD can be also used for assisting protein structure elucidation [37] or deciphering atomic details of drug–receptor interactions and binding pose prediction [38]. On the other hand, scoring functions are widely used for assessing the interaction energies in a high-throughput manner. Thanks to simplifications used for their construction, they are very fast, yet not very accurate. Nevertheless, even a moderately accurate ranking of potential drugs of abuse would have immense significance for regulatory purposes [39], and this is why scoring functions were checked for their ability to reproduce our experimental data.

Our aims determine the structure of the contribution, and so we first describe the results of the binding assays for 21 compounds (Figure 1), including two novel derivatives. Then the modeling of the fentanyl derivatives with the μOR is reported. We discuss binding modes and receptor characteristics as found in molecular dynamics simulations, focusing on how they could explain the affinity trends. Last, we recount our attempt to reproduce the binding data with popular scoring functions. 

## 2. Results and Discussion

### 2.1. Receptor Binding Determination

The studied set of 21 fentanyl derivatives (Figure 1) was tested for affinity towards the μOR in a competitive radioligand displacement assay. In order to ascertain the results’ uniformity, the assay was performed in one laboratory, with one protocol, using homogenate, reagents and materials of one batch, by a constant group of personnel, and in a few consecutive workdays. The results are presented in Table 1 as IC_50_. The obtained values span from 0.19 nM for carfentanil (**F15**) to 977.2 nM for 3″,4″-dimethoxyfentanyl (**F07**). For one derivative (3″,4″-dimethoxy-*para*-trifluoromethylfentanyl, **F08**), the IC_50_ value could not be established as the compound did not exhibit any detectable radioligand displacement at 1000 nM. The obtained results allow to reassess and mostly reconfirm the known structure–activity trends for the fentanyl family. We shall now discuss them with respect to several lines of structural variation seen in the derivatives under the study.

The parent fentanyl (**F01**) has IC_50_ = 1.23 nM. According to other results from our laboratory, this makes fentanyl as good a binder as peptidic biphalin (IC_50_ = 1.4 nM) [40], but better than morphine (IC_50_ = 4.02 nM). The replacement of the rigid piperidine core for acyclic aliphatic topographic equivalent brings about an almost 50 times larger IC_50_ value (**F02**, IC_50_ = 60.25 nM). Thus, the resulting *N*-[3-(methyl-phenethylamino)propyl]-*N*-phenyl propionamide (**F02**) seems also to be able to place properly the pharmacophoric elements in the receptor binding site, however only upon paying a relatively high entropic penalty which gives a decrease of binding affinity. To the best of our knowledge, the synthesis of **F02** was reported before [41], however no data as to its analgesic activity or opioid receptor affinity were disclosed. The here presented binding data are in line with pharmacological characteristics of another acyclic derivative 2,3-seco-fentanyl for which it was found that it is about 40 times weaker an analgesic than fentanyl [42]. 

Shortening of the N-phenethyl chain is unfavorable for binding. The removal of only one –CH_2_– group (*N*-benzyl derivative, **F03**) results in a nearly 400-fold drop in affinity (**F03**: IC_50_ = 489.7 nM). An exchange for N-thienmethyl group gives also a significantly worse binding (**F04**: IC_50_ = 245.5), however almost two times better compared to the derivative with the isosteric N-benzyl.

Regarding the substitutions at the *N*-phenethyl group, the introduction of the α-methyl yields a subnanomolar IC_50_ (**F05**: IC_50_ = 0.32 nM), while the β-hydroxy group gives a modest decrease of this value (**F06**: IC_50_ = 2.81 nM). If however the β-OH substitution is upgraded by a simultaneous introduction of a methyl group at the position 3 of piperidine (**F14**), a 10-fold improvement of IC_50_ value is noted. 

A dramatic drop is observed upon the 3″,4″-dimethoxy substitution at the N-chains’ phenyl (**F07**: IC_50_ = 977.2 nM) and the affinity is not restored with the simultaneous *para*-CF_3_ substitution at another aromatic ring (**F08**: IC_50_ > 1000 nM). To the best of our knowledge, **F07** and **F08** derivatives are reported for the first time. They seem to highlight adverse influence of polar groups at the *N*-phenethyl end. 

A similar, unfavorable effect, yet not such a spectacular one, is found at the opposite propionamide pole of the fentanyl structure. Here, the introduction of the hydroxy group at either ω-position (**F10**) or ω-1-position (**F11**) gives IC_50_ values of 97.7 nM and 489.0 nM, respectively. Contrarily, a modest apolar expansion with one additional –CH_2_– in a cyclopropyl group (**F09**) yields a subnanomolar IC_50_ value, and so the cyclopropylfentanyl (**F09**) binds to μOR approximately 1.6 times better than the parent fentanyl (**F01**). This result is also important due to the role cyclopropylfentanyl has in the ‘fentanyl crisis’. The compound synthesized in 1960s by the group of Janssen never entered the clinic, and was rather forgotten [43]. However, in recent years it has been associated with numerous cases of overdose deaths in the USA [44], and also found in seized drug samples in Europe [45]. Binding data for **F09** were unknown for many years, but while we were running our experiments, the DEA reported the compound to be a very strong μOR binder and agonist with EC_50_ better than that of fentanyl [44], which is in line with our results.

As to the phenyl ring of the propionanilide part, isosteric replacement in the *para*-position for the fluoro group is highly beneficial for binding, with IC_50_ value being 0.48 nM for **F12**, that is almost 2.6 times better than that for fentanyl. On the other hand, bulkier trifluoromethyl substituent in the *para*-position (**F13**) brings about a significant drop of μOR-binding as its IC_50_ value is 95.5 nM.

A very favorable influence on μOR affinity comes from the 4-carbomethoxy substitution at the piperidine ring. The resulting carfentanil (**F15**) is one of the strongest synthetic opioids known and it is the strongest μOR binder in our dataset, with IC_50_ value of 0.19 nM. Lofentanil (**F16**), which contains an additional 3-methyl group in the piperidine core, is of similar affinity (0.208 nM). Remifentanyl (**F17**), that is a carfentanil’s derivative with N-phenylethyl exchanged for N-(2-methoxycarbonylethyl) chain, also has a subnanomolar IC_50_ of 0.60 nM. Still, some decrease of **F17** affinity once again highlights the superiority of the N-phenethyl chain. Dramatically, its role is seen in the case of norcarfentanil (**F18**) whose IC_50_ value equals 295.1 nM. This result can be considered in a twofold aspect. On the one hand, 4-carbomethoxy substituent seems insufficient to provide very strong μOR-binding on its own. On the other hand however, anchoring obtained by this group is powerful enough to maintain relatively low IC_50_ values, even in the absence of the seemingly crucial pharmacophoric element as N-phenethyl. 

Among the 4-methoxymethyl derivatives in our set, sufentanil (**F21**) is the best μOR binder. Its IC_50_ value of 0.40 nM is not much worse than that of carfentanil (**F15**). The addition of a 3-methyl group at the piperidine core of sufentanil however yields a drop in the affinity to a similar level as parent fentanyl (**F20**: IC_50_ = 1.1 nM). An even greater decrease is found for alfentanil (**F19**), in which 4-methoxymethyl group in piperidine is accompanied by the exchange of N-phenethyl for *N*-{1-[2-(4-ethyl-5-oxo-4,5-dihydro-1*H*-1,2,3,4-tetrazol-1-yl)ethyl]. In our study, we found alfentanil’s IC_50_ to be 38.9 nM. It seems that this value is worse compared to fentanyl than what one could expect based on the previous results in the literature [46].

### 2.2. Modeling of Fentanyl Derivatives Bound to the μ-Opioid Receptor

In order to provide the structural basis behind the observed structure–affinity relationships, we have modeled the complexes of **F01**–**F21** (including the stereoisomers, where applicable) with μOR. Then the complexes were subject to short molecular dynamics simulations (three replicas of 50–80 ns length) so to determine stability of the obtained binding poses and to look for similarities and dissimilarities in the dynamic behavior of the complexes.

#### 2.2.1. Binding Modes

Previously we have proposed the binding mode for the parent fentanyl (**F01**) [47]. There is a widely held assumption that similar ligands ought to bind in a similar manner. Following it, we found the initial binding mode guess for compounds **F02**–**F21** by superimposing their structures over the fentanyl’s structure as found in the final snapshots of our simulations presented in the antecedent paper [47]. Such complexes were subject to minimizations so to remove strain possibly introduced by this specific manual docking procedure and ascertain even apportionment of water molecules in the binding site. Then the complexes were passed to regular molecular dynamics. 

**Figure 2:**
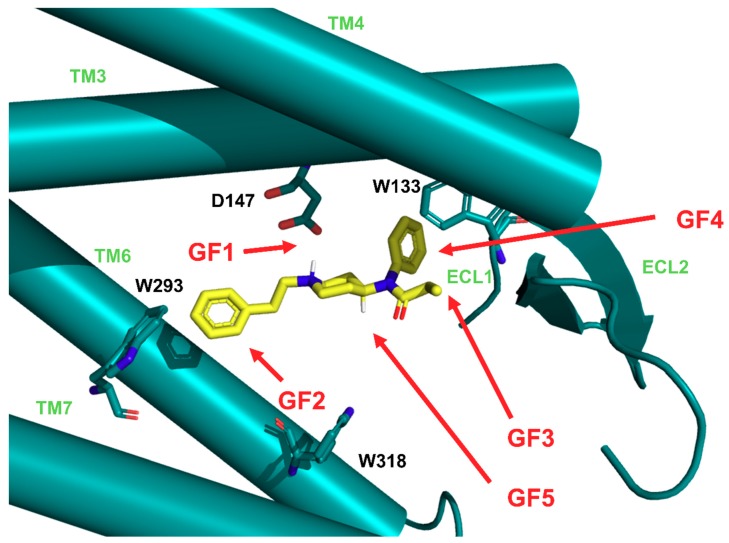
Fentanyl (in yellow) in the binding pocket of μ-opioid receptor (μOR, in light ‘Tiffany’ blue). The receptor is presented in a simplified and schematic manner with only several residues shown (labelled with black) and transmembrane helices (TM, labelled with light green) depicted as cylinders. Marked are the general features (GF, red arrows) of the binding mode common to the studied derivatives (see text for explanations).

In the case of majority of the studied derivatives, the ligand did not deviate much from the starting pose during the simulations. The exceptions were three compounds with 3*R*,4*S*-stereochemistry at the piperidine ring, for which a major rearrangement occurred: **F14RRS**, **F14SRS**, **F20RS** (however this remark does not apply to another 3*R*,4*S*-derivative: **F16**). For most of the compounds we arrived at a binding mode similar to the binding mode of the parent fentanyl. The general features (**GF**, Figure 2) of this binding mode are:

- **GF1.** ionic interaction between D147 and the protonable amine of the ligands’ piperidine,
- **GF2.** orientation of the N-chain towards the interior of the receptor,
- **GF3.** positioning of the other ligands’ pole towards the receptor outlet,
- **GF4.** locating the aromatic ring of the anilide in the subpocket formed by transmembrane helices (TM) TM3, TM4 and extracellular loops (ECL): ECL1 and ECL2,
- **GF5.** 4-axial substituent (if present) directed towards W318.

**Figure 3:**
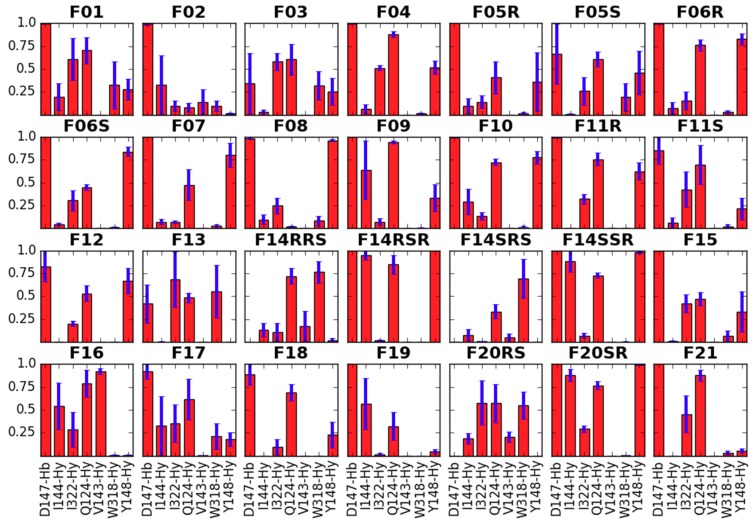
Interactions of the core piperidine with the receptor residues. Red bars represent the mean frequency of contacts with a particular residue, and blue bars show standard error of the mean. Data are collected from last 10 ns of production in 3 replicas. In *x*-axis label given is the residue’s name and number as well as type of the contact (Hb—hydrogen bond, Hy—hydrophobic, A—aromatic).

The first feature (**GF1**) is obviously expected for all good binders of μOR or more generally of any aminergic GPCR. In the past, site-directed mutagenesis experiments have shown the key importance of NH^+^···D147 interaction for affinity of several μOR agonists [48,49]. Such direct evidence is not known for the fentanyl family, however most ‘carba’-derivatives synthesized so far were rather poor μOR binders [50,51]. In simulations of the majority of the studied compounds this interaction is present (except for the suspected outliers **F14RRS**, **F14SRS**, **F20RS**). It is also very stable throughout the trajectories and in the last 10 ns it is populated for more than 99% of time for most derivatives (Figures SM-DIS-1 and SM-DIS-2 in Supplementary Materials). In a few instances, the breaking of the interaction occurs for some fraction of considered frames, however neither a pattern nor a relationship with the (diminished) experimental binding affinity can be recognized. Only for **F03** and **F13**, for which the interaction is absent in 67% and 41% of times, respectively, it can be said that this fits the diminished IC_50_ values for these derivatives. Apart from this key ionic interaction (**GF1**), the core piperidine of fentanyls interacts also with receptor residues: Y148, W318, Q124, I322 and I144 (Figure 3).

As to the second general feature (**GF2**) that is the positioning of the N-phenethyl (or another N-chain in the cases of several derivatives) towards the receptor interior, we are not aware of any direct experimental hint in its favor. If however one supposes that fentanyl’s N-phenethyl and this moiety in N-phenethylmorphine are counterparts, and at the same time N-phenethylmorphine takes the binding mode similar to that found for β-funaltrexamine in crystallographic structure 4DKL [52], then such positioning is very likely. 

**Figure 4:**
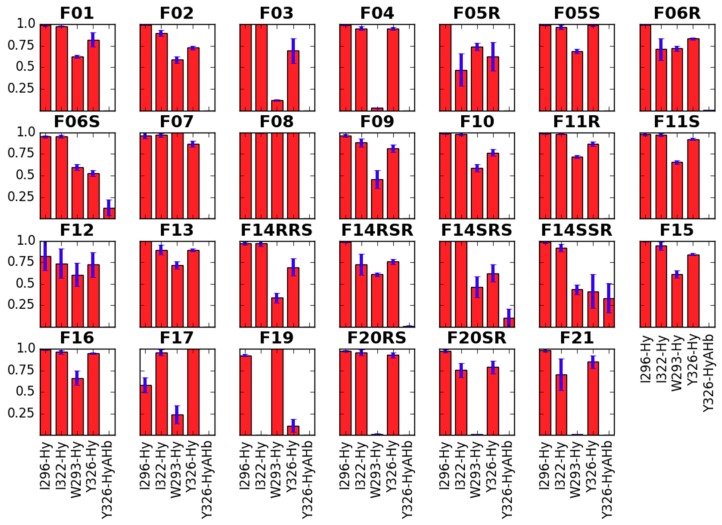
Interactions of the N-substituent with the receptor residues. Red bars represent the mean frequency of contacts with a particular residue, and blue bars show standard error of the mean. Data are collected from last 10 ns of production in 3 replicas. In *x*-axis label given is the residue’s name and number as well as type of the contact (Hb—hydrogen bond, Hy—hydrophobic, A—aromatic).

In our simulations, the N-chain forms several hydrophobic contacts with the residues at the binding site’s bottom: I296, I322, W293 and Y326 (Figure 4). The interaction with I296 is sampled for over 75% of the analyzed simulation time in almost all derivatives with the N-substituent, the exception being **F17**. I322 is also nearly always present as interaction partner for all derivatives but **F19**. This compound has on the other hand a 100% sampling of the contact with W293. It is also **F07** and **F08** that interact with W293 with 100% frequency. Among other derivatives, sampling of this interaction varies. It is significantly below 25% for derivatives with shorter N-substituents (**F03** or **F04**), and also for those bearing the N-thioethyl chain (**F20RS**, **F20SR** and **F21**). As to the Y326, majority of the studied compounds form the hydrophobic interaction with this residue approximately for more than 75% of the analyzed frames. A notable exception is **F19**.

**Figure 5:**
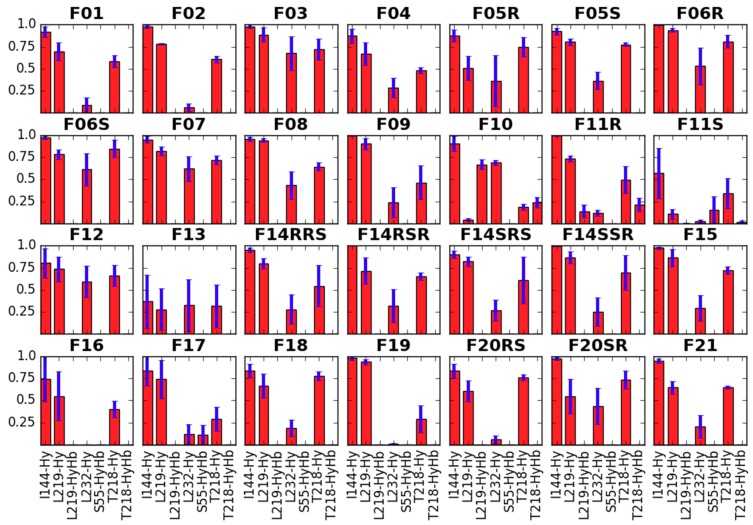
Interactions of the propanamide chain with the receptor residues. Red bars represent the mean frequency of contacts with a particular residue, and blue bars show standard error of the mean. Data are collected from last 10 ns of production in 3 replicas. In *x*-axis label given is the residue’s name and number as well as type of the contact (Hb—hydrogen bond, Hy—hydrophobic, A—aromatic).

Another ‘pole’ of the molecule—the propanamide chain is located up the binding pocket, towards the receptor outlet (**GF3**). Predominantly it forms hydrophobic interactions with I144 and L219 (Figure 5). For L232 and T218, the interaction frequencies vary with the derivative. The compounds which contain a hydroxy-function at this chain (**F10**, **F11R** and **F11S**) can also form hydrogen bonds with L219 (backbone), S55 (hydroxyl) and T218 (hydroxyl). Notably however, **F11R** and **F11S** do not hold H-bonds for more than 25% of the considered simulation time, while for **F10** hydrogen bond to L219 is present in slightly more than 50% of analyzed frames and, and to T218 in no more than 25% frames.

**Figure 6:**
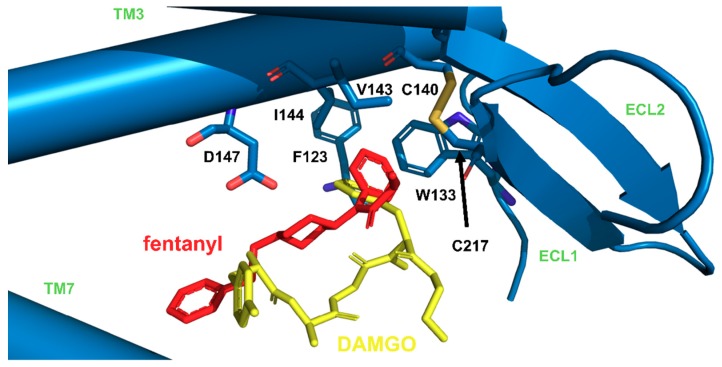
Imposition of fentanyl (red, our simulations) and DAMGO (yellow, PDB structure: 6DDF [53]) in μOR (blue), with focus on similar positioning of fentanyl anilide’s phenyl and phenyl of N-Me-Phe^4^ residue of DAMGO.

**Figure 7:**
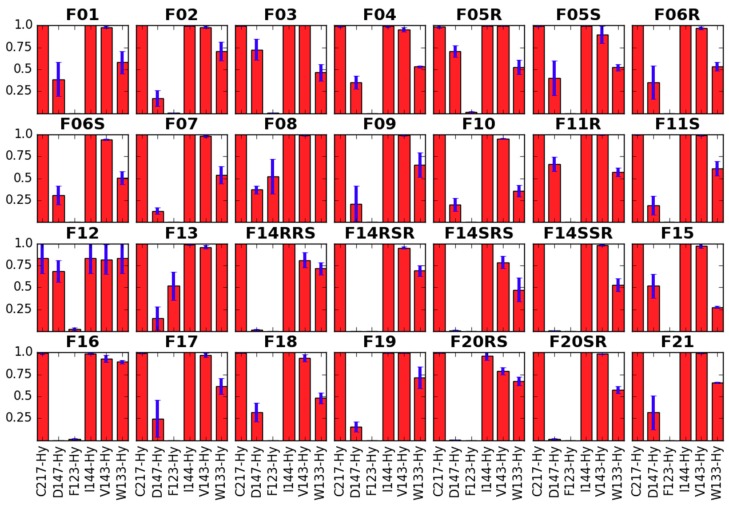
Interactions of the anilide’s aromatic ring with the receptor residues. Red bars represent the mean frequency of contacts with a particular residue, and blue bars show standard error of the mean. Data are collected from last 10 ns of production in 3 replicas. In *x*-axis label given is the residue’s name and number as well as type of the contact (Hb—hydrogen bond, Hy—hydrophobic, A—aromatic).

In our simulations, the aromatic ring of the propionanilide is located in a shallow ‘subpocket’ formed by TM3, TM4, ECL1 and ECL2 (**GF4**). This is a localization very close to the one occupied by the phenyl of DAMGO’s N-Me-Phe^4^ residue, as seen in recently published experimental structures of μOR (PDB accession codes: 6DDE and 6DDF; Figure 6 [53]). This ‘subpocket’ is confined by sidechains of C217, I144, V143 and W133 as well as by D147. It is these residues that interact with the anilide’s aromatic ring (Figure 7). Particularly frequent are contacts with C217, I144 and V143, while the interaction with W133 is sampled less often.

**Figure 8:**
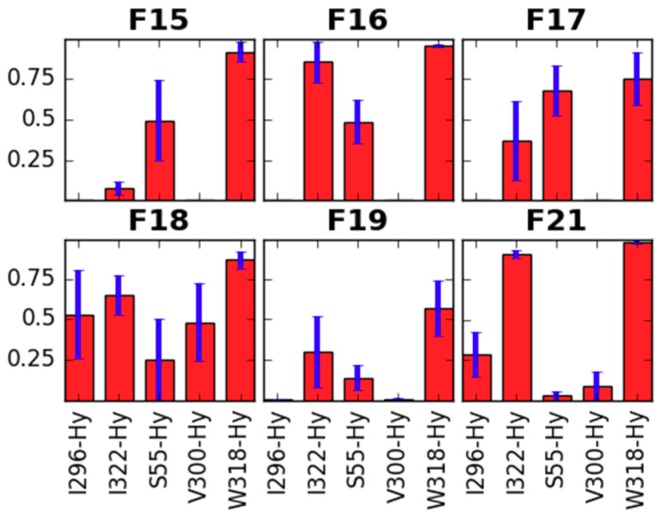
Interactions of the 4-axial substituent with the receptor residues. Red bars represent the mean frequency of contacts with a particular residue, and blue bars show standard error of the mean. Data are collected from last 10 ns of production in 3 replicas. In *x*-axis label given is the residue’s name and number as well as type of the contact (Hb—hydrogen bond, Hy—hydrophobic, A—aromatic).

The last general feature of the binding modes (**GF5**) is shared by those derivatives that have a 4-axial substituent (**F15**–**F19**, **F21**). The substituent is directed towards W318 and forms frequent interactions with this residue (Figure 8). Other frequent partners for this part of the studied molecules include residues: I322 and S55. Furthermore, in the case of **F21** and **F18**, it is also I296 that interacts with the 4-axial substituent, and for **F18**, we observe also some contacts with V300.

As to the outliers mentioned at the beginning of this section (**F14RRS**, **F14SRS**, **F20RS**), we assume that their positioning found in the simulations does not represent a true binding mode. It is a common opinion that all potent binders of μOR should have an ionic interaction between piperidine’s nitrogen and D147. In our simulations of μOR with **F14RRS**, **F14SRS**, **F20RS**, this interaction was disrupted and the piperidine was oriented upside down with the piperidine’s NH^+^ completely inaccessible for interactions with D147. While not necessarily false from the fundamental point of view, such a situation is highly unlikely in the light of the knowledge gathered on μOR/GPCR pharmacology. In particular, when we recall that **F14RRS** and **F14SRS** belong to the most potent fentanyl’s derivatives and most potent μOR binders at all [54,55]. With these objections, we rather do not comment on the results of these outlying simulations, nevertheless keeping them in the Figures for the sake of comprehensiveness.

#### 2.2.2. Binding Modes Compared to SAR Data

The presented binding mode is in our opinion able to rationally explain many of the trends observed in fentanyl’s SAR data, including the ones provided in the present contribution and those reported previously. Particularly interesting comments can be made about the N-substituent and anilide’s aromatic.

With regard to the N-substituent, our experimental data show decrease of binding with shortening of the N-chain (**F01** > **F04** and **F03**; **F15** >> **F18**). Consistently, the simulations show less apolar interactions of the N-chain in the case of shorter derivatives. On the other hand, a significant increase of the N-chain volume is rather unfavorable for binding. For instance, upon the introduction of 3″,4″-dimethoxy modification to the ring (**F07** and **F08**), the IC_50_ value drops dramatically. According to simulations, the methoxy substituents do not participate in hydrogen bonds, however, their presence increases the packing and the number of hydrophobic contacts (Figure 4). 

This increase in intermolecular interactions between the N-chain and the receptor can be reconciled with the experimental lowering of affinity, by recalling a so-called ‘55%-rule’, formulated in the field of supramolecular chemistry [56]. The rule states that the optimal situation for apolar binding occurs when 55% of the available host space is occupied by the guest. This reasoning has been successfully applied also for several cases of biological molecular recognition [57,58]. For our purposes, we resign from calculating of what precisely the optimal 55% of packing in μOR subsites is. This is because these ‘subsites’ are not well-confined, besides due to dynamic behavior of the ligand and binding site side chains, appropriate calculation must not be straightforward. The rule can be however translated into a qualitative description that both under- and overpacking is not favorable for binding (underpacking due to suboptimal number of interactions and overpacking due to entropically unfavorable rigidification of binding partners). This is what seems in fact present in our data. If we plot the pIC_50_ values against the N-chain volume (calculated as average over last 10 ns of simulations in three replicas, in order to take into account the flexibility of the chain, see Methods for explanations), the data can be approximately modeled by two linear curves, ascending and descending, suggesting that up to a certain optimum of substituent’s volume, the pIC_50_ improves, and beyond it declines (Figures SM-VOL-1 and SM-VOL-2). For 4-axially substituted derivatives, one can fit a quadratic curve, with perfect coefficient of correlation R = 0.99 (Figure SM-VOL-3). 

As to anilide’s aromatic, majority of the compounds in the present contribution contain a simple undecorated phenyl ring. However, **F12** as well as **F13** and **F08** have *para*-substituents (*p*F or *p*CF_3_). Experimental data order this small ‘subseries’ the following way: **F12** (*p*F) > **F01** (H) > **F13** (*p*CF_3_) >> **F08** (*p*CF3, and 3″,4″-diMeO). As described in Section 2.2.1, the aromatic ring is predicted to locate in an apolar subsite according to our simulations. The ring forms several hydrophobic contacts, and their frequency increases with *p*F substitution, but increases even further with the bulkier *p*CF_3_ substituent. This group is however so large that it provides full, unfavorable, packing of the subsite. In the case of **F13**, this overpacking can be partially relieved by a slight exsertion of the aromatic out of the subsite, accompanied however by the simultaneous loosening and breaking of the key ionic contact NH^+^···D147 (Figure 3; Figures SM-DIS-1 and SM-DIS-2). This is impossible in the case of **F08**, which apart from *p*CF_3_ substitution contains a voluminous modification (3″,4″-diMeO) at the phenyl of the N-phenethyl. The provided reasoning fits well with other experimental data on *para*-substituted fentanyl derivatives [59].

**Figure 9:**
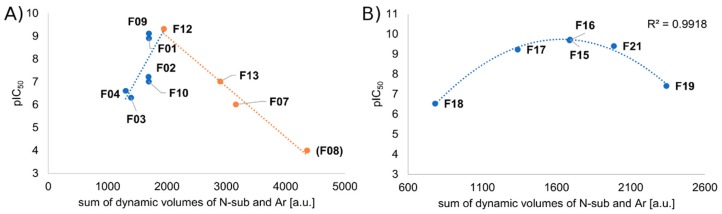
Plot of pIC_50_ against sum of dynamic volumes of N-substituent and anilide’s aromatic: **A**) without 4-axially substituted derivatives, **B**) for 4-axially substituted derivatives. Volume data are collected from last 10 ns of production in 3 replicas. Chiral compounds excluded. pIC_50_ value for **F08** (a point in brackets) arbitrary (experimental pIC_50_ < 6.0).

It seems that the volume/affinity relationships considered separately from the perspective of N-substituent and anilide’s aromatic, can be effectively joined together. Summing volumes of both these pharmacophoric elements into one variable and plotting it with the affinity data, gives a clear relationship between these two values, further supporting our qualitative reasoning provided above (Figure 9). Again the data can be modeled for 4-axially unsubstituted derivatives by two linear curves, ascending and descending, while for those bearing a 4-axial substituent, there is a quadratic curve that can be fitted with a perfect R = 0.99. Thus, in approximation, expanding the molecule volume by expanding either the N-substituent or anilide’s aromatic, or both, is favorable to a certain point. 

Another important trend observed in fentanyl’s structure–activity relationships is that many 4-axial substitutions are not only tolerated but also highly beneficial for binding. According to our simulations, 4-axial position of piperidine can have relatively large (long) substituents, as there is plenty of space for their accommodation (**F15**–**F19**, **F21**). The literature knows of fentanyl derivatives exhibiting strong μOR binding, with 4-axial substituents such as carbomethoxy (carfentanil **F15**), methoxymethyl (sufentanil **F21**), phenyl, pirymidynyl [60] and even larger ones are associated with not so pronounced decreases of affinity. The simulations do not seem however to point to any particular preference with respect to which substituent would be optimal.

As to the ω-modifications, ω-hydroxy (**F10**) and ω-1-hydroxy (**F11R** and **F11S**) groups are unfavorable to binding, since they do not form stable hydrogen bonds. On the other hand in the receptor there are some partners available for hydrogen bonding in these environs. Thus it is not surprising that some other derivatives with functions able to participate in H-bonding in this part of the fentanyl structure are not so bad binders. Such cases include derivatives where –C_2_H_5_ of the propanamide were replaced with –CH_2_OCH_3_ or furoyl [61,62]. Another ω-modification in our dataset, that is addition of –CH_2_– and cyclisation to yield a cyclopropyl substituent (**F09**), is favorable to binding by the virtue of increasing frequency of lipophilic contacts (Figure 5).

Here, we shall restrain from discussing the important issue of structural basis of stereochemical preferences in ohmefentanyl (or other 3-substituted derivatives) isomers. As stated in Section 2.2.1, we assume that we have not identified the binding modes of **F14RRS** and **F14SRS**.

#### 2.2.3. Receptor Characteristics from MD Simulations

As a next step in our analysis, we wanted to see whether the presence of particular derivatives is associated with some changes in receptor characteristics. We compare:

1. the RMSF of protein helices,
2. side chain dihedral angles of key residues,
3. the length of the so-called ‘3-7 lock’ and
4. the hydration of D114 and Y336.

All analyses are done on data from the last 10 ns of three simulations for each derivative.

##### RMSF of Protein Helices

The RMSF (root mean square fluctuation) of protein backbone or RMSF of the backbone of separate helices expresses numerically to what extent a given protein part deviates (fluctuates) over time. It thus gives some measure of system flexibility which is connected to receptor action. For example, Chan et al. analysis of protein (or protein’s helices) RMSFs derived from simulations of β_2_-adrenergic receptor with agonists, inverse agonists and antagonists, found statistically significant differences between the values obtained for these groups [63]. 

In simulations for our derivatives, the RMSF values of the protein backbone (Table SM-RMS-1, Figure SM-RMS-1) are on average 0.93 Å with a standard deviation of 0.05 Å. It means that the protein backbone RMSF does not provide a strong differentiation within the fentanyl family, as for the majority of cases the difference between the means for single derivatives is smaller than the intra-derivative differences. Indeed, both Kruskall–Wallis test and less conservative one-way ANOVA find that medians/means do not differ significantly at α = 0.05. The same applies to RMSFs of each single TM helix (Tables SM-RMS-2 to SM-RMS-8, Figures SM-RMS-2 to SM-RMS-8). We were also not able to find any structure- or affinity-based trend for these values (Table SM-RMS-9). Perhaps the only clear regularity is that in as much as five out of eight cases (H1, H2, H5, H6 and protein RMSFs) the highest mean RMSF is found for the **F08** which is the weakest binder and at the same time the most voluminous derivative in our set. 

##### Side Chain Dihedral Angles of Key Residues

Regarding the side chain dihedral angles of key residues we have considered the amino acids:

1. interacting with the fentanyl piperidine (or close to it: D147, Y148),
2. close to 4-axial substituent (W318, H319),
3. close to the N-substituent (M151, W293, H297, Y326),
4. close to anilide’s aromatic (Y133) or even
5. outside the binding pocket (Y336).

The distributions as well as time evolutions for these dihedrals are given in Supplementary Materials (Figures SM-DIH-1 to SM-DIH-40). Short descriptions are given in Table SM-DIH-1. For most of the analyzed dihedrals the values are stable during the analyzed periods and belong to a single cluster (Y148 X_1_, W318 X_1_, W318 X_2_, H319 X_1_, W293 X_1_, W293 X_2_, H297 X_1_, H297 X_2_, Y326 X_1_, W133 X_1_, W133 X_2_, Y336 X_1_). For a few, another minor cluster appears (D147 X_1_, H319 X_2_, M151 X_1_, Y326 X_1_, Y336 X_2_) or more clusters are present (D147 X_2_, Y148 X_2_, M151 X_1_). The analyzed dihedrals do not seem to differentiate the derivatives except for W318 X_2_, W293 X_2_ and W133 X_2_. Regarding the first of these, W318 X_2_, the mean value can be as different as −116.0º (**F06R**) and −93.2º (**F11R**). The order however does not seem to be straightforwardly related to structure of the derivatives. On the contrary, the remaining two dihedrals (W293 X_2_ and W133 X_2_) are clearly related to derivatives’ volume. 

**Figure 10:**
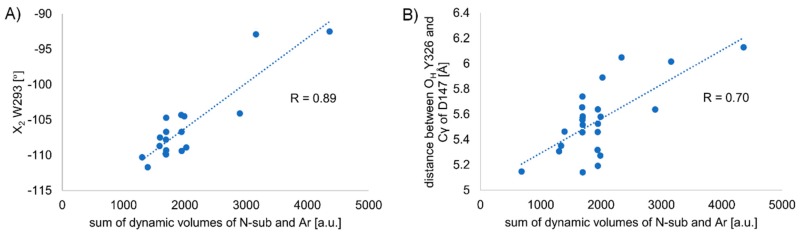
Correlation of volumes with receptor characteristics: **A**) mean W293 X_2_ dihedral measures against the sum of dynamic volumes of N-substituent and anilide’s aromatic. R = 0.89, *n* = 18. Excluded are 4-axially substituted derivatives as well as **F14** stereoisomers, **B**) mean distance between O_H_ Y326 and Cγ of D147 against the sum of dynamic volumes of N-substituent and anilide’s aromatic. R = 0.70, *n* = 24. Excluded are **F14RRS**, **F14SRS** and **F20RS** (suspected binding mode outliers).

**Figure 11:**
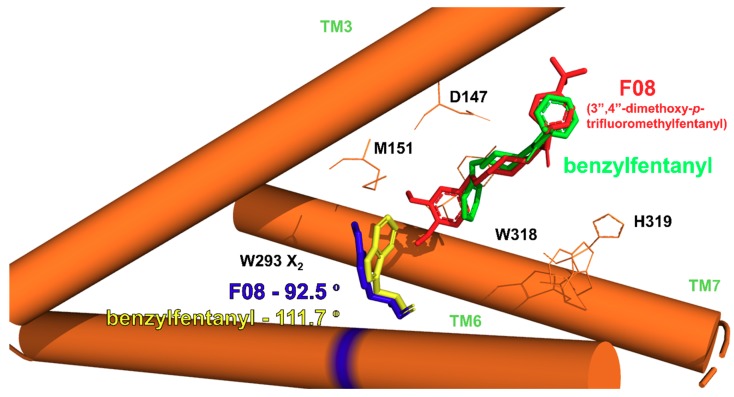
Average dihedral W293 X_2_ for simulations with **F08** (red ligand, blue W293) and benzylfentanyl (**F03**, green ligand, yellow W293) shown with the respective ligands and a simplified representation of μOR binding site.

In the case of W293 X_2_, the values vary between −92.0º (**F19**) and −111.7º (**F03**). There is a correlation between the dihedral measure and the sum of dynamic volumes of the N-substituent and the anilide’s aromatic (correlation coefficient R = 0.75, *n* = 28, Figure SM-VOL-4; upon exclusion of 4-axially substituted derivatives R = 0.89, *n* = 18, Figure 10A). The correlation states that the larger is the ligand the less negative is the value of the dihedral. Speaking structurally, it means that larger derivatives “push” the tryptophan ring to a conformation more perpendicular to TM6 axis, while for smaller derivatives the ring is slightly kinked into the binding cavity (Figure 11). The relationship between fentanyl core substituents and the W293 X_2_ dihedral value seems most interesting as the W293 is commonly known to be involved in the receptor activation processes. GPCRs activation includes a series of structural rearrangements of the hydrophobic residues in the core of the receptor that lead to major rearrangements of the helices enabling the binding of the intracellular partners, including G-proteins or β-arrestins. W293 is a residue that transmits communication between the μOR orthosteric binding site and the hydrophobic receptor core. In the past, it had been postulated that the residue functions as a rotameric micro-switch [64,65], and for many GPCRs mutating this residue (X6.48, where mostly X = W or F) affects signaling [66,67,68]. Interestingly, for a closely-related δ-opioid receptor, it has been found that a W6.48L mutation almost abolishes agonist-dependent β-arrestin recruitment, while having only a moderate impact on G-protein signaling [69]. It is then tempting to hypothesize based on the relationship we observe here and on the knowledge on the importance of W293 for signaling that manipulating the size of the core substituents (at least in the fentanyl family) may provide means to rationally influence the signaling efficacies. 

In the case of W133 X_2_, the spread of mean values is less pronounced and they vary between –95.2º (**F18**) and 104.3º (**F08**). Here again, we observe a correlation between the sum of substituents’ volumes and the dihedral value (R = 0.70, *n* = 28, Figure SM-VOL-5; upon exclusion of 4-axially substituted derivatives, R = 0.74, *n* = 18, Figure SM-VOL-6). For this dihedral, the relationship is inverse compared to the one just discussed above and the larger is the ligand the more negative is the dihedral value.

##### Length of the so-Called ‘3-7 lock’

Another receptor characteristic that had been considered important from the point of view of opioid receptor activation is the hydrogen bond between D147 and Y326, the so-called ‘3-7 lock’ [64,65,70]. As the hydrogen bosnd is present in both inactive [52] and active [53,71] experimental structures of μOR, the experimental data does not allow a simple reception of older hypotheses inferred by comparison with rhodopsin and from modeling that the breaking of this ‘lock’ participates (or even initiates) the μOR activation [65]. Furthermore, in our previous work [47] we observed the breaking of the lock in simulations with both the activated as well as inactive structure of the empty (unliganded) receptor. Still, we consider the distance between O_H_ Y326 and Cγ of D147 an informative descriptor of the binding site. The mean values for this characteristic differ in the range 5.14 to 6.13 Å, meaning that there is no hydrogen bond between the two residues. Notably again, we observe a relationship between the summed substituents volume and the value of this characteristic (R = 0.70, *n* = 24). Thus, in first approximation, the bulkier the substituents at the piperidine core, the larger the distance between O_H_ Y326 and Cγ of D147 (Figure 10B).

##### Hydration of D114 and Y336

The last element of our analysis of receptor dynamic characteristics is the hydration of two key residues connected to activation (D114) and intracellular interactions (Y336). The MD-derived hydration of these residues (or of their counterparts in other GPCRs) have been reported to be associated with receptor function and ligands’ activity [63,72,73,74]. We have monitored the hydration by counting the number of water molecules within 5.0 Å from any of the atom of a given residue. The distributions and time evolutions of these values are given in Figures SM-WAT-1 to SM-WAT-4, and the mean values in Tables SM-WAT-1 and SM-WAT-2. It seems that the hydration of these residues is not much influenced by the presence of particular derivative (no significant difference between the means in one-way ANOVA at α = 0.05).

### 2.3. Scoring

Having gathered a uniform experimental data on binding of the derivatives to μOR, we were curious if they can be reproduced computationally by 14 scoring functions used for molecular docking. Scoring is a simplified way of estimating the strength of interaction between ligands and proteins. It is widely used in docking since it is very fast but due to simplifications in most cases, it is also very inaccurate [75]. Most scoring functions can be relatively successful for screening purposes to choose probable binders out of a variety of chemotypes. Unfortunately, frequently they cannot properly order closely related compounds as to their binding affinity against a particular target [76]. 

**Figure 12:**
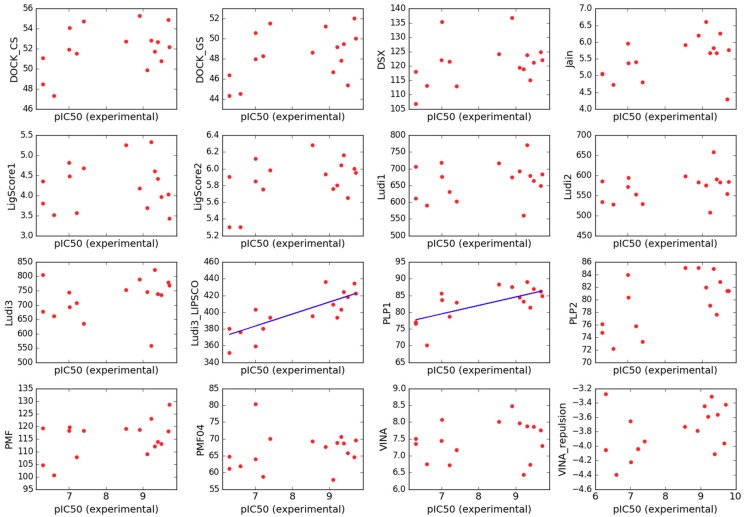
Plots of binding estimation by scoring functions (arbitrary units) against the experimental pIC_50_. The above plots include only the limited subset (without chiral compounds, **F07**, **F08** and **F18**) See Methods Section for scoring functions labels.

The same holds true for our dataset. None of the tested scoring functions were able to rank the derivatives so that the correlation coefficient between the experimental and predicted data was R > 0.30 (*n* = 28, Figure SM-SCO-1). Upon exclusion of chiral compounds (for which it is not straightforward to select proper experimental values as we have performed binding assays for enantiomeric mixtures), the correlation between experiment and prediction did not improve much (Figure SM-SCO-2). Particularly striking was the fact the most of the scorings estimated the poorest binder **F08** (IC_50_ > 1000 nM) as a very strong one, even as the best in the whole set. With excluding yet this derivative as well as **F07** and **F18** (which were frequent outliers in correlations experiment/prediction), we were able to arrive at R = 0.64 (*n* = 13, Figure 12) for correlation between PLP1 scoring and the experiment.

Interestingly, considering components constituting the scoring functions, we found that the *LUDI3_LIPSCO* element which describes the contribution to binding coming from lipophilic interactions, is by far the best estimator of the binding in this limited subset (R = 0.78, *n* = 13, Figure 12). Therefore we decided to reweight the components of LUDI3 scoring function equation so that it better fit the experimental data. An expression for reweighted function (*LUDI3_reweighted*) is given in Supplementary Information (Figure SM-SCO-3). The coefficient optimization obviously brought improvement in internal validation (R = 0.84, Figure SM-SCO-4), however the reweighted function (*LUDI3_reweighted*) failed in even very modest external validation tests. For example, we wanted to see if it would at least correctly classify several very strong μOR binders (morphine, biphalin, gu-biphalin, PZM21, endomorphine-1, endomorphine-2; IC_50_ obtained in our laboratory in 0.8–10 nM range) as of similar affinity to that of fentanyl, however, *LUDI3_reweighted* was not able to do so. 

Importantly to note, while we were working on this paper, an interesting paper appeared whose authors set themselves aims similar to ours. They wanted to see whether a scoring function could accurately reproduce experimental binding data [39]. This attempt included 23 opioids among which there were eight fentanyl derivatives, eight morphine derivatives and seven other μOR ligands of rather further structural similarity to fentanyl (‘fentanyl congeners’ as per authors’ term). By the means of Molecular Operating Environment suite (GBVI/WSA scoring function) [77], they docked and scored their set and compared it to the experimental data. The fentanyl-binding pose that they found is different than the one we report here. While the N-phenethyl chain is also directed towards the receptor interior as in our work, it interacts with H297 (aromatic stacking) and in our model the chain is closer to Y326. Further, contrarily to the results of ours, the authors found a strong correlation between the score and experimental binding constants for fentanyl derivatives and ‘congeners’. With their approach, it was possible to separate fentanyl derivatives into three strength classes (strong, moderate, weak binders) based on the scoring result. On the other hand, the method gave rather poor correlation for the group of morphine derivatives. Nevertheless, the workflow described in that paper holds some promise of practical utility for assisting in preliminary characterization of newly identified designer drugs.

## 3. Materials and Methods 

### 3.1. Fentanyl Derivatives

**Figure 13:**
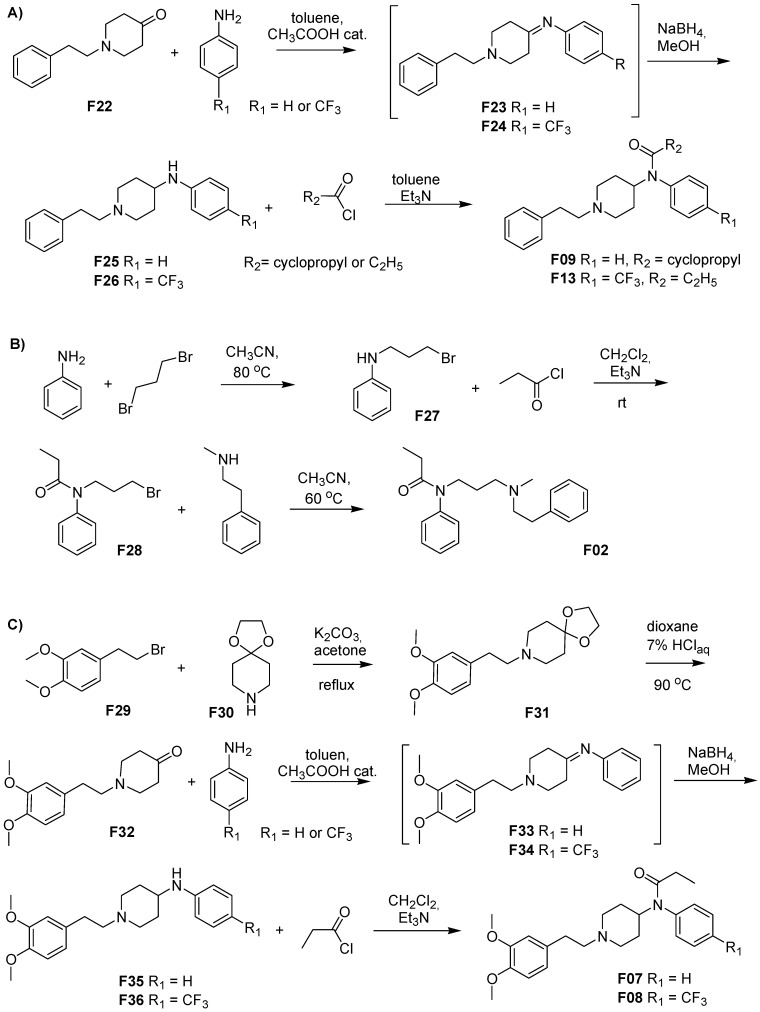
Syntheses of fentanyl derivatives **A**) **F09** and **F13**, **B**) **F02 C**) **F07** and **F08**.

Compounds **F01**, **F03**–**F06**, **F10**–**F12** and **F14**–**F21** were purchased from Toronto Research Chemicals, North York, ON, Canada and used without further processing. Compounds **F02**, **F07**, **F08**, **F09**, **F13** were synthesized at our facilities as presented in Figure 13. Among them, **F07** and **F08** have never been reported before, as per our knowledge. Synthetic details for derivatives **F02**, **F09** and **F13** are given in Supplementary Materials. Some of the steps reported therein include previously described procedures or data [41,43,59,78,79].

### 3.2. Chemistry

All reagents were purchased from commercial suppliers and used without further purification. The NMR spectra were recorded on a Bruker Avance spectrometer (Bruker, Karlsruhe, Germany) operating at 300 MHz for ^1^H-NMR and 75 MHz for ^13^C-NMR. The spectra were measured in CDCl_3_ and are given as δ values (in ppm) relative to TMS. Mass spectra were collected on LCT Micromass TOF HiRes apparatuses (Micromass UK Limited, Manchester, UK). Melting points were determined on a Melting Point Meter KSP1D (A. Krüss Optronic, Hamburg, Germany) and were uncorrected. TLC analyses were performed on silica gel plates (Merck Kiesegel GF_254_, Merck, Darmstadt, Germany) and visualized using UV light or iodine vapor. Column chromatography was carried out at atmospheric pressure using Silica Gel 60 (230–400 mesh, Merck, Darmstadt, Germany) using appropriate eluents.

### 3.3. Synthesis

#### 3.3.1. 8-[2-(3,4-Dimethoxyphenyl)ethyl]-1,4-dioxa-8-azaspiro[4.5]decane (**F31**)

The suspension of 1,4-dioxa-8-azaspiro[4.5]decane **F30** (0.219 g, 1.53 mmol, 1 equiv) and potassium carbonate (1.06 g, 7.65 mmol, 5 equiv) in acetone (15 mL) was stirred at 23–24 °C for 1 h. After this time 3,4-dimethoxyphenethyl bromide **F29** (0.40 g, 1.84 mmol, 1.2 equiv) was added and the resulting suspension was stirred at reflux for 4 h. The solvent was evaporated in vacuum and to residue CH_2_Cl_2_ (20 mL) and water (15 mL) were added. After phase separation the water phase was additionally extracted with CH_2_Cl_2_ (2 × 20 mL). The organic extract was dried over MgSO_4_, filtered and the solvent was removed in vacuo. The product was isolated by column chromatography on silica gel (CHCl_3_) as white solid (0.32 g, 68%). mp = 92–93 °C.

^1^H-NMR (300 MHz, CDCl3) δ 6.77 (m, 3H), 3.96 (bs, 4 H), 3.87 (s, 3H), 3.85 (s, 3H), 2.75 (m, 2H), 2.61 (m, 4H), 1.78 (t, *J* = 5.7 Hz, 2H). ^13^C-NMR (75 MHz, CDCl3) δ 148.8, 147.3, 133.1, 120.5, 112.0, 111.3, 107.2, 64.2, 60.3, 55.9, 55.8, 51.3, 34.9, 33.6. LR-MS (ESI): 308.3 (M + H)^+^.

#### 3.3.2. 1-(3,4-Dimethoxyphenethyl)-piperidin-4-one (**F32**)

To a stirred at 23–24 °C solution of **F31** (0.230 g, 0.748 mmol, 1 equiv) in dioxane (1.5 mL) distilled water (6 mL) and concentrated hydrochloric acid (1.5 mL) were added and the resulting solution was heated at 90 °C for 4.5 h. After cooling to reaction mixture water (8 mL) was added, alkalized with solid potassium carbonate and extracted with chloroform (3 × 20 mL). The organic extract was dried over MgSO_4_, filtered and the solvent was removed in vacuo. The product was isolated by column chromatography on silica gel (CHCl_3_) as colorless solid (0.18 g, 91%). mp = 80–81 °C (lit. mp = 76–78.5 °C [80])

^1^H-NMR (300 MHz, CDCl3) δ 6.73 (m, 3H), 3.87 (s, 3H), 3.85 (s, 3H), 2.79 (m, 6H), 2.70 (m, 2H), 2.47 (t, *J* = 6.0 Hz, 2H). ^13^C-NMR (75 MHz, CDCl3) δ 208.9, 148.9, 147.4, 132. 6, 120.5, 112.0, 111.3, 59.4, 55.9, 55.8, 53.1, 41.2, 33.7. LR-MS (ESI): 264.3 (M + H)^+^.

#### 3.3.3. *N*-(1-Phenethyl-4-piperidinyl)-(3,4-dimethoxyphenyl)amine (**F35)**

**F35** was prepared as reported in Ref. [78]. A solution of 1-(3,4-dimethoxyphenethyl)-piperidin-4-one **F32** (0.270 g, 1.02 mmol, 1 equiv), aniline (0.094 mL, 1.02 mmol, 1 equiv) and acetic acid (1 drop) in toluene (12 mL) was heated under reflux with a Dean-Stark apparatus for 24 h. The toluene was removed in vacuo and the residue was used for the next step without further purification. 

The crude imine **F33** was dissolved in methanol (10 mL) and NaBH_4_ (0.078 g, 2.05 mmol, 2 equiv) was added gradually at 23 °C to the stirred solution during 10 min. The obtained solution was stirred for 1 h at 60 °C. After evaporation of the solvent, water (5 mL) was added and the mixture was extracted with chloroform (3 × 15 mL). The organic extract was dried over MgSO_4_, filtered and the solvent was removed in vacuo. The product **F35** was isolated by column chromatography on silica gel (CHCl_3_:MeOH, 0–1% MeOH) as solidifying oil (0.28 g, 81%). 

^1^H-NMR (300 MHz, CDCl3) δ 7.16 (t, *J* = 7.8 Hz, 2H), 6.77 (m, 3H), 6.68 (t, *J* = 7.5 Hz, 1H), 6.60 (d, *J* = 7.8 Hz, 2H), 3.87 (s, 3H), 3.85 (s, 3H), 3.48 (bs, 1H), 3.32 (m, 1H), 2.96 (d, *J* = 11.7 Hz, 2H), 2.76 (m, 2H), 2.59 (m, 2H), 2.20 (t, *J* = 11.4 Hz, 2H), 2.09 (d, *J* = 12.6 Hz, 2H), 1.51 (m, 2H). ^13^C-NMR (75 MHz, CDCl3) δ 148.8, 147.3, 147.1, 133.0, 129.3, 120.5, 117.2, 113.2, 112.0, 111.3, 60.8, 55.9, 55.8, 52.5, 49.9, 33.5, 32.6. LR-MS (ESI): 341.4 (M + H)^+^.

#### 3.3.4. 3″,4″-Dimethoxyfentanyl, *N*-(1-Phenethyl-4-piperidinyl)-*N*-(3,4-dimethoxyphenyl)-propionamide (**F07**)

To a stirred at 23–24 °C solution of **F35** (0.144 g, 0.423 mmol, 1 equiv) and triethylamine (0.065 mL, 0.465 mmol, 1.1 equiv) in dichloromethane (8 mL) solution of propanoic acid chloride (0.041 mL, 0.465 mmol, 1.1 equiv) in dichloromethane (1 mL) was added and the resulting solution was additionally stirred for 30 min. After this time, CH_2_Cl_2_ (10 mL) and water (4 mL) were added, the mixture was shaken and the phases were separated. The organic extract was dried over MgSO_4_, filtered and the solvent was removed in vacuo. The product **F07** was isolated by column chromatography on silica gel (CHCl_3_) as white solid (0.130 g, 78%). mp = 78–79 °C.

^1^H-NMR (300 MHz, CDCl3) δ 7.38 (m, 3H), 7.08 (m, 2H), 6.77 (m, 1H), 6.69 (m, 2H), 4.70 (tt, *J* = 12.3, 3.9 Hz, 1H), 3.84 (s, 3H), 3.82 (s, 3H), 2.99 (d, *J* = 11.7 Hz, 2H), 2.67 (m, 2H), 2.52 (m, 2H), 2.16 (t, *J* = 11.4 Hz, 2H), 1.93 (q, *J* = 7.5 Hz, 2H), 1.81 (d, *J* = 12.0 Hz, 2H), 1.43 (m, 2H), 1.02 (t, *J* = 7.5 Hz, 2H). ^13^C-NMR (75 MHz, CDCl3) δ 173.5, 148.8, 147.3, 138.8, 132.9, 130.43, 129.3, 128.2, 120.4, 112.0, 111.2, 60.5, 55.9, 55.8, 53.1, 52.1, 33.4, 30.5, 29.7, 28.5, 9.6. LR-MS (ESI): 397.5 (M + H)^+^.

#### 3.3.5. *N*-(1-Phenethyl-4-piperidinyl)-(4-trifluoromethylphenyl)amine (**F36**)

**F36** was prepared as reported in Ref. [78]. A solution of 1-(3,4-dimethoxyphenethyl)-piperidin-4-one **F32** (0.270 g, 1.02 mmol, 1 equiv), trifluoromethylaniline (0.129 mL, 1.02 mmol, 1 equiv) and acetic acid (1 drop) in toluene (12 mL) was heated under reflux with a Dean-Stark apparatus for 24 h. The toluene was removed in vacuo and the residue was used for the next step without further purification. 

The crude imine **F34** was dissolved in methanol (10 mL) and NaBH_4_ (0.078 g, 2.05 mmol, 2 equiv) was added gradually at 23 °C to the stirred solution during 10 min. The obtained solution was stirred for 1 h at 60 °C. After evaporation of the solvent, water (5 mL) was added and the mixture was extracted with chloroform (3 × 15 mL). The organic extract was dried over MgSO_4_, filtered and the solvent was removed in vacuo. The product **F36** was isolated by column chromatography on silica gel (CHCl_3_:MeOH, 0%–2% MeOH) as colorless oil (0.26 g, 62%). 

^1^H-NMR (300 MHz, CDCl3) δ 7.39 (d, *J* = 9.0 Hz, 2H), 6.80 (m, 1H), 6.75 (m, 2H), 6.58 (d, *J* = 9.0 Hz, 2H), 3.87 (s, 3H), 3.86 (s, 3H), 3.35 (m, 1H), 2.97 (d, *J* = 12.0 Hz, 2H), 2.77 (m, 2H), 2.61 (m, 2H), 2.22 (t, *J* = 12.0 Hz, 2H), 2.08 (d, *J* = 12.0 Hz, 2H), 1.53 (m, 2H). ^13^C-NMR (75 MHz, CDCl3) δ 149.5, 148.8, 147.4, 132.9, 126.6 (q, *J* = 3.0 Hz), 123.2, 118.5 (q, *J* = 33.0 Hz), 112.1, 112.05, 111.3, 60.7, 55.9, 55.8, 52.3, 49.6, 33.5, 32.2. LR-MS (ESI): 409.3 (M + H)^+^.

#### 3.3.6. 3″,4″-Dimethoxy-*para*-trifluoromethylfentanyl, *N*-(1-Phenethyl-4-piperidinyl)-*N*-(4-trifluoromethylphenyl)-propionamide (**F08**)

To a stirred at 60 °C solution of **F36** (0.120 g, 0.294 mmol, 1 equiv) and triethylamine (0.049 mL, 0.352 mmol, 1.2 equiv) in toluene (6 mL) solution of propanoic acid chloride (0.031 mL, 0.352 mmol, 1.2 equiv) in toluene (1 mL) was added and the resulting solution was additionally stirred for 30 min. After this time, toluene (8 mL) and water (4 mL) were added, the mixture was shaken and the phases were separated. The organic extract was dried over MgSO4, filtered and the solvent was removed in vacuo. The product **F08** was isolated by column chromatography on silica gel (CHCl_3_:Me)H, 0%–2% MeOH) as white solid (0.104 g, 76%). mp = 102–103 °C.

^1^H-NMR (300 MHz, CDCl3) δ 7.68 (d, *J* = 9.0 Hz, 2H), 7.23 (t, *J* = 9.0 Hz, 2H), 6.76 (m, 1H), 6.69 (m, 2H), 4.72 (t, *J* = 12.0 Hz, 1H), 3.84 (s, 3H), 3.83 (s, 3H), 3.00 (d, *J* = 12.0 Hz, 2H), 2.68 (m, 2H), 2.51 (m, 2H), 2.16 (t, *J* = 12.0, 2.1 Hz, 2H), 1.92 (q, *J* = 9.0 Hz, 2H), 1.82 (d, *J* = 12.0 Hz, 2H), 1.38 (m, 2H), 1.04 (t, *J* = 9.0 Hz, 2H). ^13^C-NMR (75 MHz, CDCl3) δ 172.9, 148.8, 147.3, 142.3, 132.8, 131.0, 126.5 (q, *J* = 3.7 Hz), 125.5, 120.4, 117.4, 112.0, 111.2, 60.5, 55. 9, 55.7, 53.0, 52.3, 33.4, 30.7, 28.7, 9.5. LR-MS (ESI): 465.4 (M + H)^+^.

### 3.4. Binding Assays

The binding affinity of compounds **F01**–**F21** for μOR was determined in competitive radioligand binding assays in rat brain homogenates [40]. The homogenates were prepared as follows. Brain tissue, obtained from rats euthanized by decapitation (cerebellum removed), was homogenized in a hand glass homogenizer and suspended in Tris-HCl (pH 7.4). The preparation was centrifuged (at 23,000 rpm, 4 °C for 15 min) and the supernatant was disposed. The remaining precipitate was resuspended in Tris-HCl buffer and incubated at 25 °C for 30 min, after which the centrifugation and resuspension were repeated again. 

The so-obtained membrane fractions (containing opioid receptors) were incubated at 25 °C for 60 min in the presence of 0.5 nM [^3^H]DAMGO (a radioligand specific for μOR) bought from PerkinElmer, USA, and the increasing concentrations of the assayed compounds (3.0 × 10^−10^ up to 10^−5^ M, each concentration in duplicate). The assay buffer was made of 50 mM Tris-HCl (pH 7.4), bovine serum albumin (BSA, 0.1 mg/mL), bacitracin (50 μg/mL), bestatin (30 μM), captopril (10 μM) and phenylmethane sulfonyl fluoride (PMSF, 30 μg/mL) in a total volume of 1 mL. Additionally, for measuring non-specific binding, 10 μM naltrexone was added. 

After the incubation, a rapid filtration with a M-24 Cell Harvester (Brandel, Biomedical Research & Development Laboratories, Inc., Gaithersburg, MD, USA) through GF/B Whatman glass fiber strips was done. The filters were soaked with 0.5% PEI just before harvesting so that the extent of non-specific binding be minimized. After placing filter discs separately in 24-well plates, Optiphase Supermix scintillation solution (Perkin Elmer, Inc., Waltham, MA, USA) was added to each well. Radioactivity was measured in a scintillation counter MicroBeta LS, Trilux (Perkin Elmer, Inc., Waltham, MA, USA). The experiments were performed in duplicate.

### 3.5. Molecular Modeling

#### 3.5.1. Receptor and Ligand Preparation

For the purposes of molecular modeling, complexes of studied fentanyl derivatives (**F01**–**F21**) with μOR were built. The protein structure was taken from our previous work [47]. It was a processed 5C1M (active-state μOR crystal) [71] structure after 2.1 μs of full-atom molecular dynamics simulations with fentanyl in the binding site (receptor embedded in lipid bilayer). Originally, the protein structure was downloaded from the OPM (Orientation of Proteins in Membranes) database [81] that contains structures pre-oriented with respect to the membrane normal. The protein included neither the nanobody nor crystallographic adjuvants found in the original 5C1M PDB entry [71]. A few missing atoms were added automatically and unnatural amino acid cysteine-s-acetamide (YCM) found in position 57 was manually changed to cysteine. 

This protein structure served as a template for fentanyls-μOR complexes. The complexes were formed by imposing structures of **F02**–**F21** over the respective fentanyl **F01** atoms in the template. Such initial complexes were subjected to minimization so as to remove possible strain resulting from manual docking and to ascertain even apportionment of water molecules in the binding site. Regarding the ligand structures, the piperidine N-atoms were protonated as expected at physiological pH values. In order to obtain force field parameters for the ligands, they were submitted to ParamChem service that produces parameters compatible with CHARMM force-field (CHARMM CGenFF approach [82]). 

In case of chiral compounds, enantiomers were included in modeling as follows: (a) for α- and β- and ω-1-positions both R and S isomers, (b) for isomerism connected to two chiral centers at positions 3 and 4 of the piperidine: only *cis*-isomers.

The crystal structure for the modeling is of murine origin, while our experimental data were acquired with rat brain preparations and fentanyl derivatives are widely used in human medicine. However, as the sequence identity of human/murine/rat μOR is very high, and the singular sequence differences are rather conservative and remote from the binding site, we have assumed they should have no impact on simulations and scoring results. In the Supplementary Materials (SM-SEQ section) we provide the sequences’ alignment and a comment thereon.

#### 3.5.2. Molecular Dynamics

The MD simulations were performed in GROMACS 5.1.2 [83]. The systems subject to dynamics contained ligands, μOR protein, POPC membrane (145 lipid molecules), water (about 13,000 water molecules, TIP3P), as well as Na^+^ and Cl^−^ ions that were added so as to obtain 0.154 M concentration. For the protein, lipids, water, and ions, CHARMM 36 force field was used, while for ligands CHARMM CGenFF. The complexes built were subject to minimization and equilibration, after which the production step (NPT ensemble, 303.15 K, integration step = 2 fs, cut-off scheme Verlet, Nose-Hoover thermostat, Parrinello–Rahman barostat, LINCS H-bonds constraints) followed. The production was run so to obtain at least 50 ns of equilibrated trajectory.

#### 3.5.3. Analysis of Data from Molecular Dynamics

For the purposes of MD data analysis, we have created a snapshot pool for each derivative containing 3000 snapshots. They were extracted from last 10 ns of three production runs, 100 snapshots per 1 ns. The snapshot pool was subject to further analyses, that is to calculation of receptor/ligand characteristics, dynamic volume or scoring.

The characteristics computed on the snapshot pools included:

- root-mean-square-deviation (RMSD) of protein backbone atoms with a 0.0 ns structure taken as reference; RMSDs were calculated for separate helices, or for all them together; the residues considered for each helix are: 72-92 (H1), 104-128 (H2), 139-169 (H3), 185-204 (H4), 227-260 (H5), 275-304 (H6), 314-338 (H7);
- distance between ligands’ protonable nitrogen and Cγ atom of D147;
- X_1_ and X_2_ dihedral angles of: W133, D147, Y148, W293, H297, W318, H319, Y326, W336;
- distance between Cγ atom of D147 and O_H_ atom of Y326;
- hydration of D114 and Y336 residues, understood as a number of water molecules found within 5.0 Å of any atom of a given residue.

These characteristics were gathered by the means of GROMACS utilities [83] (RMSD, distances, dihedrals) or by our in-house scripts (hydration).

For each considered property, we provide its time evolutions in the Supplementary Materials. It is also there that we give for each derivative distributions of the properties’ values in the snapshot pool. For comparative purposes, the properties were averaged (arithmetic means for distances, RMSDs, number of water molecules; circular means for dihedral angle values). RMSDs and hydrations were subject to the Kruskall–Wallis test and one-way ANOVA analysis (at α = 0.05), using in-house Python scripts based on *numpy* and *scipy* libraries.

#### 3.5.4. Volume Calculations

For the purposes of volume calculations, ligand geometries were extracted from the MD snapshot pool (100 snapshots per 1 ns, from the final 10 ns of three replicas). The ligands were fragmented into five parts: piperidine, N-chain, anilide’s aromatic ring, propanamide. The fragments were subject to a single point quantum mechanical calculation at the HF/6-31G* with PCM implicit solvent model, in Gaussian 09 [84]. Electronic spatial extent (ESE) value (in atomic units) was gathered for each snapshot and the averaged. The average ESE was taken as a measure of ‘dynamic volume’ of a given element. Calculation for many snapshots, followed by averaging, was undertaken in order to account for rotational flexibility and this is why we call the average ESE a ‘dynamic volume’.

#### 3.5.5. Scoring

The snapshots of MD trajectories (snapshot pool) for each derivative were scored using 14 different scoring functions. The structures were taken directly from MD trajectories and after removal of water molecules, they were subject to default processing (with default parameters) in particular programs. 

The following scoring functions were used:

- Jain scoring function (Jain) [85],
- Piecewise Linear Potential (PLP1 and PLP2) [86],
- Potential of Mean Force (PMF and PMF04) [87,88],
- LigScore scoring function (LigScore1 and LigScore2) [89],
- Ludi scoring function (Ludi1, Ludi2 and Ludi3) [90],
- VINA scoring function (VINA) [91],
- DSX scoring function (DSX) [92],
- DOCK continuous and grid scores (DOCK_CS and DOCK_GS) [93].

The scoring preceded with any required preparatory procedures (grid calculations, atom typing etc.) was performed using Accelrys Discovery Studio suite (Jain, PLP1, PLP2, PMF, PMF04, LigScore1, LigScore2, Ludi1, Ludi2, Ludi3) [94], AutoDock VINA (VINA) [91], DSX standalone program (DSX) [92], or DOCK 6.8 (DOCK_CS and DOCK_GS) [93]. For each scoring function, the optimal score was collected and used for further analyses. The optimal here means maximum or minimum depending on the type of scoring function. Analyses with averaged scores were also performed but as they gave qualitatively similar results they are not discussed in the paper.

#### 3.5.6. Regression Analysis and Molecular Graphics

Regression analysis was performed using in-house scripts built in Python scripts, based on *numpy* and *scipy* libraries, as well as in Microsoft Excel. Coefficient optimization of LUDI scoring function was done in Microsoft Excel. Molecular graphics were prepared in PyMol 2.1.1 (open source project maintained by Schrödinger, LLC, New York, NY, USA), [95]. 

## 4. Conclusions

To sum up, we analyzed interactions of 21 fentanyl derivatives with μOR by the means of experimental and theoretical methods. First, a uniform assessment of fentanyls’ binding to μOR was performed with competitive radioligand binding assay. Two of the studied derivatives have never been published before, and the binding affinity data are also novel for one compound previously known. In general, our binding assay results confirm the structure–activity trends reported before. However thanks to uniform conditions, our dataset is placed on a uniform scale what supports and facilitates comparisons.

Molecular modeling performed provides us with hypothetical structural basis behind the observed trends. Based on the results of molecular dynamics we propose that the binding modes of the studied derivatives share five common General Features (**GFs**): **GF1**) ionic interaction between D147 and the protonable amine of the ligands’ piperidine moiety, **GF2**) orientation of the N-chain towards the receptor interior, **GF3**) positioning of the other ligands’ pole towards the receptor outlet, **GF4**) locating the aromatic ring of the anilide in the subpocket formed by TM3, TM4, ECL1 and ECL2, **GF5**) positioning of the 4-axial substituent (if present) towards W318. Apart from a single ionic interaction (**GF1**), most contacts with the receptor are hydrophobic. Consistently with the apolar character of the binding, it seems that there is a relationship between the volume of the N-chain and/or anilide’s aromatic ring and the binding affinity. The relationships appear to be nonlinear, with optima for medium substituents and decreasing of affinity for both smaller and larger ones. According to molecular dynamics, smaller N-chains participate in a lower number of interactions (underpacking and hence lower binding affinity), while for the larger ones, the number of contacts can increase, however it should result in larger entropic penalty due to rigidification of interacting partners (overpacking).

Furthermore, molecular dynamics of fentanyls-μOR complexes reveal several interesting relationships between receptor characteristics and the volumes of the fentanyls’ N-chain and anilide’s aromatic ring. For instance there is a linear correlation between the volumes and average dihedral angles of W293 and W133 from simulations. In particular, the link between ligands’ volume and the W293 X_2_ dihedral seems most interesting, as this residue is commonly known to be involved in the activation processes. Another receptor characteristic dependent of the ligands’ size is the distance between the O_H_ atom of Y326 and the Cγ atom of D147. On the other hand, we found neither statistically significant differences in RMSF measures among the derivatives nor any affinity-/structure-based correlations with these measures. No such differences and no correlations could be established for the hydration of D114 or Y336.

We have also performed an attempt to reproduce our experimental binding data with 14 popular scoring functions. None of the tested functions performed in a satisfactory manner (correlation coefficient between predicted and observed data R < 0.30, *n* = 28). Only after excluding a few points, we were able to reach correlation of R = 0.64 (*n* = 13) for PLP1 or R = 0.78 (*n* = 13) for the LUDI3_LIPSCO component. Reweighting the components of the LUDI3 scoring function yielded an improvement in the internal validation, however an attempt to apply the reweighted equation to score a few good μOR binders other than fentanyl derivatives resulted in a very poor performance. Thus, the tested scoring functions leave a lot to be desired, at least in the case of fentanyls-μOR binding. Promisingly, another recent contribution [39] gives more hope in this realm.

## Figures and Tables

**Table 1:** IC_50_ values from competitive radioligand displacement assay for the derivatives under the study.

Cmpd No	Name	IC_50_^1^	SD ^2^
**fentanyl and acyclic derivative**			
**F01**	fentanyl	1.23	0.14
**F02**	*N*-[3-(Methyl-phenethylamino)propyl]-*N*-phenyl propionamide	60.25	3.51
**shorter N-chain**			
**F03**	benzylfentanyl	489.7	28.6
**F04**	thienylfentanyl	245.5	12.9
**substitution at the N-chain**			
**F05** ^3^	α-methylfentanyl	0.32	0.01
**F06** ^3^	β-hydroxyfentanyl	2.81	0.13
**substitution at the ring of the *N*-phenethyl**			
**F07**	3″,4″-dimethoxyfentanyl	977.2	43.11
**F08**	3″,4″-dimethoxy-*para*-trifluoromethylfentanyl	>1000.0	-
**variations at the propionamide chain**			
**F09**	cyclopropylfentanyl	0.77	0.04
**F10**	ω-hydroxyfentanyl	97.7	5.80
**F11** ^3^	ω-1-hydroxyfentanyl	489.0	40.59
***para*-substitution**			
**F12**	*para*-fluorofentanyl	0.48	0.03
**F13**	*para*-trifluoromethylfentanyl	95.5	6.2
**ohmefentanyl (β-OH, 3-Me)**			
**F14** ^3^	ohmefentanyl	0.27	0.03
**4-substitution**			
**F15**	carfentanil	0.19	0.01
**F16**	lofentanil	0.21	0.01
**F17**	remifentanil	0.60	0.08
**F18**	norcarfentanil	295.1	1.3
**F19**	alfentanil	38.9	2.8
**N-thioethyl**			
**F20** ^3^	3-methylothiofentanyl	1.10	0.10
**F21**	sufentanil	0.40	0.03

^1^ half maximal inhibitory concentration in nM, average of two independent experiments run in two replicas, ^2^ standard deviation, ^3^ tested as enantiomeric mixture.

## Supplementary Materials

Supplementary Materials (SM) are available online, SM file is divided into sections that contain data pertaining to particular analysis subjects. Items in each section are independently numbered. The sections are: **SM-SYN** (synthetic details for compounds **F02, F09** and **F13**) **SM-DIS** (time evolutions and distributions of distances derived from MD, 4 Figures), **SM-VOL** (correlations of volumes with affinity data, as well as with dihedral angle values, 6 Figures), **SM-RMS** (root-mean-square deviations of protein backbone, as well as of single helices; distributions, time evolutions and means; 8 Tables and 9 Figures), **SM-DIH** (dihedral angles derived from MD, summary, time evolutions and distributions, 1 Table and 40 Figures), **SM-WAT** (hydration derived from MD, time evolutions, distributions and means, 2 Tables and 4 Figures), **SM-SCO** (scoring results; correlations of predicted vs. observed values, expression for reweighted scoring function, 1 Table and 4 Figures) and **SM-SEQ** (sequence alignment of human, rat and murine μOR).
