# Supplementary material for: Fentanyl Family at the Mu-Opioid Receptor: Uniform Assessment of Binding and Computational Analysis
Source: Molecules. 2019 Feb 19;24(4):740. doi: 10.3390/molecules24040740 (PMC6412969; doi:10.3390/molecules24040740)
Supplement: Supplementary file 1 [file molecules-24-00740-s001.pdf]

## Supplementary Materials to:

# Fentanyl family at the mu-opioid receptor: uniform assessment of binding and computational analysis.

by

Piotr F. J. Lipiński,* Piotr Kosson, Joanna Matalińska, Piotr Roszkowski, Zbigniew Czarnocki, Małgorzata Jarończyk, Aleksandra Misicka, Jan Cz. Dobrowolski, Joanna Sadlej*

**Corresponding authors:** Piotr F. J. Lipiński: plipinski@imdik.pan.pl; Joanna Sadlej: j.sadlej@uksw.edu.pl

## Table of contents

**SM-SYN: syntheses of F02, F09 and F13**

**Procedure SM-SYN-1.** Cyclopropylfentanyl, Cyclopropanecarboxylic acid (1-phenethyl-4-piperidinyl)phenyl amide (F09)
**Procedure SM-SYN-2.** N-(1-phenethyl-4-piperidinyl)-(4-trifluoromethylphenyl)amine (F26)
**Procedure SM-SYN-3.** *para*-Trifluoromethylfentanyl, N-(1-phenethyl-4-piperidinyl)-N-(4-trifluoromethylphenyl)-propionamide (F13)
**Procedure SM-SYN-4.** N-(3-bromopropyl)-N-phenyl propionamide (F28)
**Procedure SM-SYN-5.** N-[3-(methyl-phenethylamino)propyl]-N-phenyl propionamide (F02)
**Listing SM-SYN-1.** References for synthetic part in Supplementary Materials.

**SM-DIS: distances**

**Figure SM-DIS-1**. Distributions of the distance between Cgamma of D147 and ligands' piperidine nitrogen.
**Figure SM-DIS-2**. Time evolution of the distance between Cgamma of D147 and ligands' piperidine nitrogen.
**Figure SM-DIS-3**. Distributions of the distance between Cgamma of D147 and phenolic O of Y326.
**Figure SM-DIS-4**. Time evolution of the distance between Cgamma of D147 and phenolic O of Y326.

**SM-VOL: volumes**

**Figure SM-VOL-1.** pIC50 values plotted against the dynamic volume of N-substituent for all compounds except for F14RRS, F14SRS and F20RS.
**Figure SM-VOL-2.** pIC50 values plotted against the dynamic volume of N-substituent.
**Figure SM-VOL-3.** pIC50 values plotted against the dynamic volume of N-substituent for 4-axially substituted derivatives.
**Figure SM-VOL-4.** Correlation of mean X2 W293 dihedral measures with the sum of dynamic volumes of N-substituent and anilide's aromatic.
**Figure SM-VOL-5.** Correlation of mean X2 W133 dihedral measures with the sum of dynamic volumes of N-substituent and anilide's aromatic.
**Figure SM-VOL-6.** Correlation of mean X2 W133 dihedral measures with the sum of dynamic volumes of N-substituent and anilide's aromatic.

**SM-RMS: RMSD measures**

**Figure SM-RMS-1.** Root mean square deviation of receptor protein backbone over time.
**Table SM-RMS-1.** Root mean square fluctuations of receptor protein backbone
**Figure SM-RMS-2.** Root mean square deviation of TM1 helix backbone over time.
**Table SM-RMS-2.** Root mean square fluctuations of TM1 helix backbone.
**Figure SM-RMS-3.** Root mean square deviation of TM2 helix backbone over time.
**Table SM-RMS-3.** Root mean square fluctuations of TM2 helix backbone.
**Figure SM-RMS-4.** Root mean square deviation of TM3 helix backbone over time.
**Table SM-RMS-4.** Root mean square fluctuations of TM3 helix backbone.
**Figure SM-RMS-5.** Root mean square deviation of TM4 helix backbone over time.
**Table SM-RMS-5.** Root mean square fluctuations of TM4 helix backbone.
**Figure SM-RMS-6.** Root mean square deviation of TM5 helix backbone over time.
**Table SM-RMS-6.** Root mean square fluctuations of TM5 helix backbone.
**Figure SM-RMS-7.** Root mean square deviation of TM6 helix backbone over time.
**Table SM-RMS-7.** Root mean square fluctuations of TM6 helix backbone.
**Figure SM-RMS-8.** Root mean square deviation of TM7 helix backbone over time.
**Table SM-RMS-8.** Root mean square fluctuations of TM7 helix backbone.
**Figure SM-RMS-9.** RMSF mean values.

**SM-DIH: dihedral angles**

**Table SM-DIH-1.** Summary of monitored dihedral angles.
**Figure SM-DIH-1.** Distributions of X1 dihedral angle values of D147.
**Figure SM-DIH-2.** Time evolution of X1 dihedral angle of D147.
**Figure SM-DIH-3.** Distributions of X2 dihedral angle values of D147.
**Figure SM-DIH-4.** Time evolution of X2 dihedral angle of D147.
**Figure SM-DIH-5.** Distributions of X1 dihedral angle values of Y148.
**Figure SM-DIH-6.** Time evolution of X1 dihedral angle of Y148.
**Figure SM-DIH-7.** Distributions of X2 dihedral angle values of Y148.
**Figure SM-DIH-8.** Time evolution of X2 dihedral angle of Y148.
**Figure SM-DIH-9.** Distributions of X1 dihedral angle values of W318.
**Figure SM-DIH-10.** Time evolution of X1 dihedral angle of W318.
**Figure SM-DIH-11.** Distributions of X2 dihedral angle values of W318.
**Figure SM-DIH-12.** Time evolution of X2 dihedral angle of W318.
**Figure SM-DIH-13.** Distributions of X1 dihedral angle values of H319.
**Figure SM-DIH-14.** Time evolution of X1 dihedral angle of H319.
**Figure SM-DIH-15.** Distributions of X2 dihedral angle values of H319.
**Figure SM-DIH-16.** Time evolution of X2 dihedral angle of H319.
**Figure SM-DIH-17.** Distributions of X1 dihedral angle values of M151.
**Figure SM-DIH-18.** Time evolution of X1 dihedral angle of M151.
**Figure SM-DIH-19.** Distributions of X2 dihedral angle values of M151.
**Figure SM-DIH-20.** Time evolution of X2 dihedral angle of M151.
**Figure SM-DIH-21.** Distributions of X1 dihedral angle values of W293.
**Figure SM-DIH-22.** Time evolution of X1 dihedral angle of W293.
**Figure SM-DIH-23.** Distributions of X2 dihedral angle values of W293.
**Figure SM-DIH-24.** Time evolution of X2 dihedral angle of W293.
**Figure SM-DIH-25.** Distributions of X1 dihedral angle values of H297.
**Figure SM-DIH-26.** Time evolution of X1 dihedral angle of H297.
**Figure SM-DIH-27.** Distributions of X2 dihedral angle values of H297.
**Figure SM-DIH-28.** Time evolution of X2 dihedral angle of H297.
**Figure SM-DIH-29.** Distributions of X1 dihedral angle values of Y326.
**Figure SM-DIH-30.** Time evolution of X1 dihedral angle of Y326.
**Figure SM-DIH-31.** Distributions of X2 dihedral angle values of Y326.
**Figure SM-DIH-32.** Time evolution of X2 dihedral angle of Y326.
**Figure SM-DIH-33.** Distributions of X1 dihedral angle values of W133.
**Figure SM-DIH-34.** Time evolution of X1 dihedral angle of W133.
**Figure SM-DIH-35.** Distributions of X2 dihedral angle values of W133.
**Figure SM-DIH-36.** Time evolution of X2 dihedral angle of W133.
**Figure SM-DIH-37.** Distributions of X1 dihedral angle values of Y336.
**Figure SM-DIH-38.** Time evolution of X1 dihedral angle of Y336.
**Figure SM-DIH-39.** Distributions of X2 dihedral angle values of Y336.
**Figure SM-DIH-40.** Time evolution of X2 dihedral angle of Y336.

**SM-WAT: hydration**

**Figure SM-WAT-1.** Distributions of the number of water molecules within 5.0 angstroms from Y336
**Table SM-WAT-1.** Mean number of water molecules within 5.0 angstroms from D114.
**Figure SM-WAT-2.** Number of waters in proximity (5.0 angstroms) of D114 over time.
**Figure SM-WAT-3.** Distributions of the number of water molecules within 5.0 angstroms from Y336
**Table SM-WAT-2.** Mean number of water molecules within 5.0 angstroms from Y336.
**Figure SM-WAT-4.** Number of waters in proximity (5.0 angstroms) of Y336 over time.

| SM-SCO: scoring |
| --- |
| **Table SM-SCO-1.** Optimal scoring values for the tested derivatives.<br>**Figure SM-SCO-1.** Plots of binding estimation by scoring functions (arbitrary units) against the experimental pIC50.<br>**Figure SM-SCO-2.** Plots of binding estimation by scoring functions (arbitrary units) against the experimental pIC50.<br>**Figure SM-SCO-3.** Expression for reweighted LUDI3 scoring function.<br>**Figure SM-SCO-4.** Plots of binding estimation by reweighted LUDI3 scoring function against the experimental pIC50. Internal validation. |

| SM-SEQ: sequence alignment |
| --- |
| **Sequence SM-SEQ-1.** Sequence alignment of human, rat and murine μOR. |

**Procedure SM-SYN-1. Cyclopropylfentanyl, Cyclopropanecarboxylic acid (1-phenethyl-4-piperidinyl)phenyl amide (F09)**

The compound **F25** was prepared according to literature.[1]

To a stirred at 23-24 ºC solution of **F25** (0.280g, 0.998 mmol, 1 equiv) and triethylamine (0.180 mL, 1.297 mmol, 1.3 equiv) in anhydrous toluene (10 mL) solution of cyclopropanecarbonyl chloride (0.0907 mL, 0.998 mmol, 1 equiv) in toluene (1 mL) was added and the resulting solution was stirred for additional 30 min. After this time toluene (20 mL) and water (8 mL) were added, mixture was shaken and phases were separated. The organic extract was dried over $MgSO_4$, filtered and the solvent was removed in vacuo. The product **F09** was isolated by column chromatography on silica gel ($CHCl_3$) as white solid (0.320 g, 92%). mp = 122-123 ºC (lit.[2] mp = 119.5-120.4 ºC).

$^1$H NMR (300 MHz, CDCl3) δ 7.38 (m, 3H), 7.25 (m, 3H), 7.16 (m, 5H), 4.67 (tt, $J$ = 11.7, 3.9 Hz, 1H), 3.00 (m, 2H), 2.73 (m, 2H), 2.53 (m, 2H), 2.15 (dt, $J$ = 12.0, 2.4 Hz, 2H), 1.81 (m, 2H), 1.47 (dq, $J$ = 12.3, 3.9 Hz, 2H), 1.09 (m, 1H), 0.97 (m, 2H), 0.54 (m, 2H). $^{13}$C NMR (75 MHz, CDCl3) δ 173.2, 140.3, 138.9, 130. 8, 129.2, 128.6, 128.4, 128.1, 126.0, 60.5, 53.2, 52.5, 33.9, 30.6, 13.2, 8.4. LR-MS (ESI +): 349.2 $(M + H)^+$.

**Procedure SM-SYN-2. N-(1-phenethyl-4-piperidinyl)-(4-trifluoromethylphenyl)amine (F26)**[1]

A solution of 1-phenethyl-piperidin-4-one **F22** (0.50 g, 2.46 mmol, 1 equiv), 4-trifluoromethylaniline (0.225 mL, 2.46 mmol, 1 equiv) and acetic acid (2 drops) in toluene (20 mL) was heated under reflux with a Dean-Stark apparatus for 24 h. The toluene was removed in vacuo and the residue was used for the next step without further purification.

The crude imine **F24** was dissolved in methanol (10 mL) and $NaBH_4$ (0.186 g, 4.92 mmol, 2 equiv) was added gradually at 23 ºC to the stirred solution during 10 min. Obtained solution was stirred for 1.5 h at 60 ºC. After evaporation of the solvent water (5 mL) was added and the mixture was extracted with chloroform (3x15 mL). The organic extract was dried over $MgSO_4$, filtered and the solvent was removed in vacuo. The product **F26** was isolated by column chromatography on silica gel ($CHCl_3$:MeOH, 0-1% MeOH) as colourless oil (0.51 g, 74%).

$^{1}H$ NMR (300 MHz, CDCl3) δ 7.38 (d, $J$ = 8.4 Hz, 2H), 7.29 (m, 2H), 7.20 (m, 3H), 6.57 (d, $J$ = 8.4 Hz, 2H), 3.88 (d, $J$ = 7.8 Hz, 1H), 3.34 (m, 1H), 2.96 (m, 2H), 2.61 (m, 2H), 2.21 (m, 2H), 2.05 (m, 2H), 1.52 (m, 2H). $^{13}C$ NMR (75 MHz, CDCl3) δ 149.5, 140.3, 128.9, 128.4, 126.6 (q, $J$ = 3.0 Hz), 126.0, 125.0 (q, $J$ = 268.5 Hz), 118.4 (q, $J$ = 32.2 Hz), 112.1, 60.5, 52.3, 49.6, 33.9, 32.3. LR-MS (ESI +): 349.3 $(M + H)^{+}$.

**Procedure SM-SYN-3. *para*-Trifluoromethylfentanyl, N-(1-phenethyl-4-piperidinyl)-N-(4-trifluoromethylphenyl)-propionamide (F13)**

To a stirred at 23-24 ºC solution of **F26** (0.270g, 0.775 mmol, 1 equiv) and triethylamine (0.140 mL, 1.007 mmol, 1.3 equiv) in anhydrous toluene (10 mL) solution of propanoic acid chloride (0.0677 mL, 0.775 mmol, 1 equiv) in anhydrous toluene (1 mL) was added and the resulting solution was stirred at 70 ºC for 3 h. After this time toluene (20 mL) and water (8 mL) were added, mixture was shaken and phases were separated. The organic extract was dried over $MgSO_4$, filtered and the solvent was removed in vacuo. The product **F13** was isolated by column chromatography on silica gel ($CHCl_3$) as white solid (0.197 g, 63%). mp = 121-122 ºC (lit.[3] mp = 237.0 ºC for HCl salt).

$^{1}H$ NMR (300 MHz, CDCl3) δ 7.88 (d, $J$ = 8.4 Hz, 2H), 7.21 (m, 7H), 4.71 (t, $J$ = 11.7 Hz, 1H), 3.00 (d, $J$ = 11.7 Hz, 2H), 2.72 (m, 2H), 2.53 (m, 2H), 2.16 (dt, $J$ = 12.0, 2.1 Hz, 2H), 1.92 (q, $J$ = 7.2 Hz, 2H), 1.82 (m, 2H), 1.38 (dq, $J$ = 12.0, 3.9 Hz, 2H), 1.04 (t, $J$ = 7.5 Hz, 2H). $^{13}C$ NMR (75 MHz, CDCl3) δ 172.90, 142.25, 140.14, 131.02, 130.62 (q, $J$ = 33.0 Hz), 128.59, 128.37, 126.47 (q, $J$ = 3.7 Hz), 126.03, 123.67 (q, $J$ = 270.0 Hz), 60.40, 52.98, 52.29, 33.83, 30.64, 28.72, 9.49. LR-MS (ESI +): 427.2 $(M + Na)^{+}$.

**Procedure SM-SYN-4. N-(3-bromopropyl)-N-phenyl propionamide (F28)**

The compound **F27** was prepared according to literature.[4]

To a stirred at 23-24 ºC solution of **F27** (0.250g, 1.177 mmol, 1 equiv) and triethylamine (0.140 mL, 1.007 mmol, 1.05 equiv) in $CH_2Cl_2$ (10 mL) solution of propanoic acid chloride (0.102 mL, 1.17 mmol, 1 equiv) in $CH_2Cl_2$ (1 mL) was added and the resulting solution was stirred additionally for 30 min. After this time $CH_2Cl_2$ (10 mL) and water (10 mL) were added, mixture was shaken and phases were separated. The organic extract was dried over $MgSO_4$, filtered and the solvent was removed in vacuo. The product **F28** was isolated by column chromatography on silica gel ($CH_2Cl_2$) as colourless oil (0.280 g, 88%).

$^{1}$H NMR (300 MHz, CDCl3) δ 7.40 (m, 3H), 7.15 (m, 2H), 3.82 (t, *J* = 7.2 Hz, 2H), 3.40 (t, *J* = 6.9 Hz, 2H), 2.12 (qv, *J* = 7.5 Hz, 2H), 2.05 (q, *J* = 7.5 Hz, 2H), 1.04 (t, *J* = 7.5 Hz, 2H). $^{13}$C NMR (75 MHz, CDCl3) δ 173.9, 142.5, 129.8, 128.1, 128.0, 48.2, 31.2, 30.5, 27.8, 9.5. LR-MS (ESI +): 270.2, 272.2 $(M + H)^+$.

**Procedure SM-SYN-5. N-[3-(methyl-phenethylamino)propyl]-N-phenyl propionamide (F02)**

The solution of N-(3-bromopropyl)-N-phenyl propionamide (0.28g, 1.04 mmol, 1 equiv) and N-methyl-phenethylamine (0.301 mL, 2.08 mmol, 2 equiv) in acetonitrile (16 mL) was stirred at 60 °C for 2 h. After this time water (15 mL) was added and the resulting mixture was extracted with diethyl ether (1x40 mL and 2x20 mL). The organic extract was dried over $MgSO_4$, filtered and the solvent was removed in vacuo. The product **F02** was isolated by column chromatography on silica gel (hexane:diethyl ether, 0-100% $Et_2O$) as colorless oil (0.150 g, 44%). (lit.[5] mp = 99.0-101.0 ºC for HCl salt).

$^{1}$H NMR (300 MHz, CDCl3) δ 7.37 (m, 3H), 7.26 (m, 2H), 7.16 (m, 5H), 3.72 (t, *J* = 7.5 Hz, 2H), 2.71 (m, 2H), 2.54 (m, 2H), 2.40 (t, *J* = 7.5 Hz, 2H), 2.24 (s, 3H), 2.02 (q, *J* = 7.5 Hz, 2H), 1.71 (qv, *J* = 7.5 Hz, 2H), 1.04 (t, *J* = 7.5 Hz, 2H). $^{13}$C NMR (75 MHz, CDCl3) δ 173.5, 142.7, 140.4, 129.6, 128.6, 128.2, 128.2, 127.7, 125.8, 59.5, 54.9, 47.5, 42.0, 33.8, 27.8, 25.8, 9.6. LR-MS (ESI +): 325.4 $(M + H)^+$.

**Listing SM-SYN-1. References for synthetic part in Supplementary Materials.**

1. Vardanyan, R.; Vijay, G.; Nichol, G. S.; Liu, L.; Kumarasinghe, I.; Davis, P.; Vanderah, T.; Porreca, F.; Lai, J.; Hruby, V. J. Synthesis and investigations of double-pharmacophore ligands for treatment of chronic and neuropathic pain *Bioorg. Med. Chem.* **2009**,*17*, 5044–5053.

2. Patent FR 1517671, 1961, N.V. Research Laboratorium Dr. C. Janssen.

3. Casy, A.F.; Huckstep, M.R. Structure-Activity Studies of Fentanyl *J. Pharm. Pharmacol.* **1988**, 40:605-608.

4. Thansandote, P; Raemy, M.; Rudolph, A.; Lautens, M. Synthesis of Benzannulated N-Heterocycles by a Palladium-Catalyzed C-C/C-N Coupling of Bromoalkylamines *Org. Lett.* **2007**, *9*, 25, 5255-5258.

5. Wright, Jr., W.B.; Brabander, H.J.; Hardy, Jr., R.A. Synthetic Analgesics. III. Basic Anilides and Carbanilates Containing the Phenalkyl Moiety *J. Org. Chem.* **1961**, *26*, 485-490.

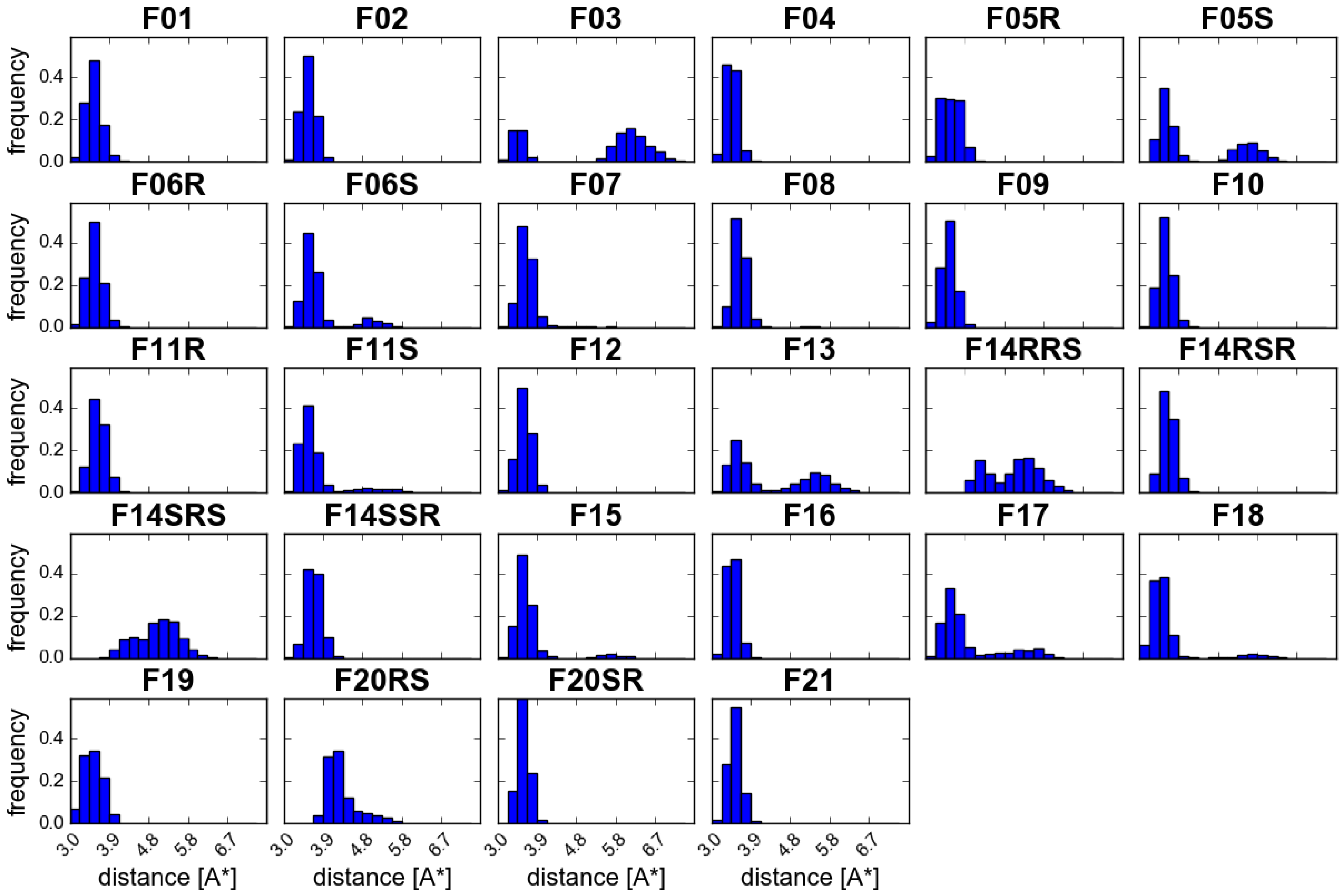


**Figure SM-DIS-1.** Distributions of the distance between Cgamma of D147 and ligands' piperidine nitrogen.
*Data are collected from last 10ns of production in 3 replicas.*

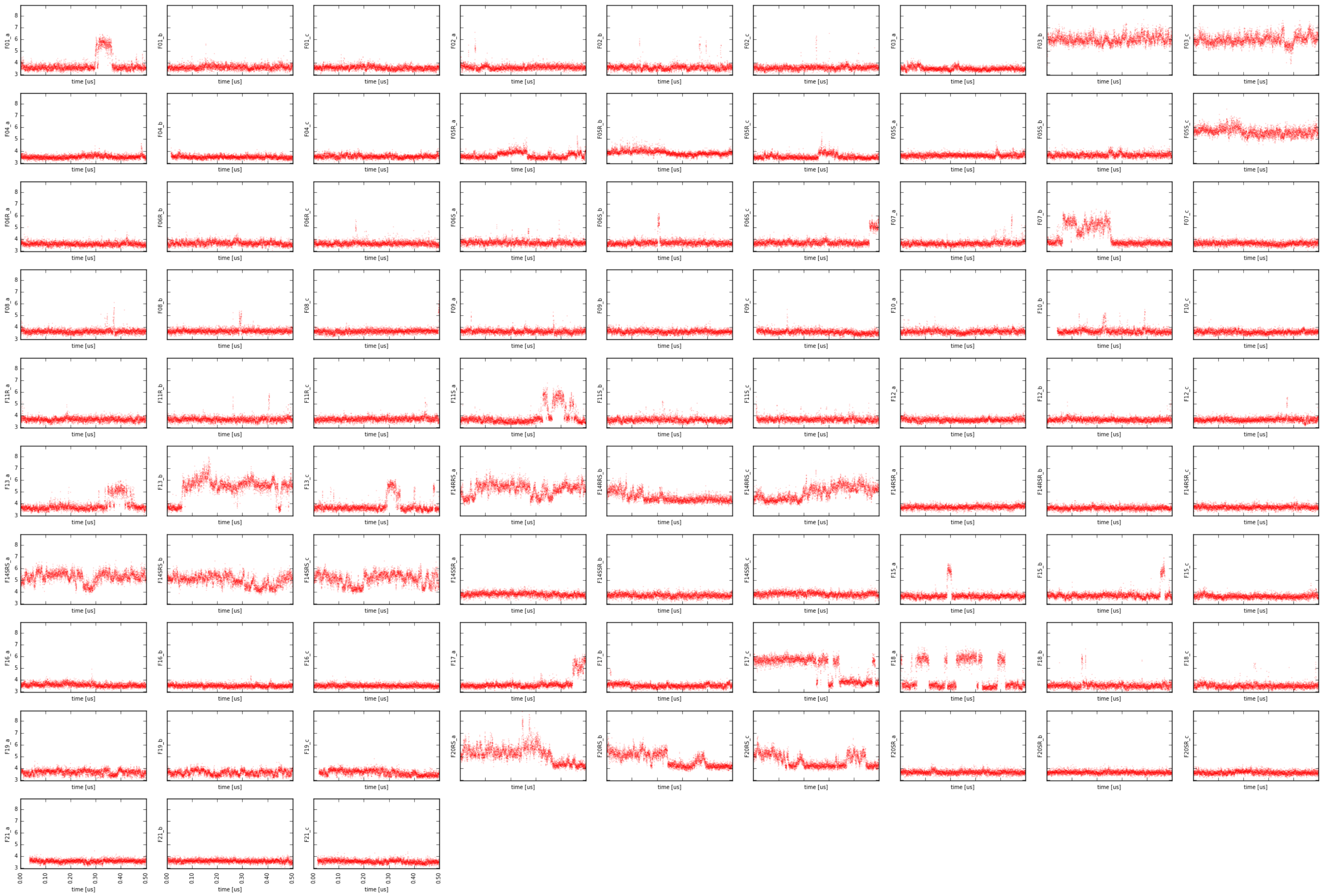

**Figure SM-DIS-2.** Time evolution of the distance between Cgamma of D147 and ligands' piperidine nitrogen. *Y-values are given in angstroms.*

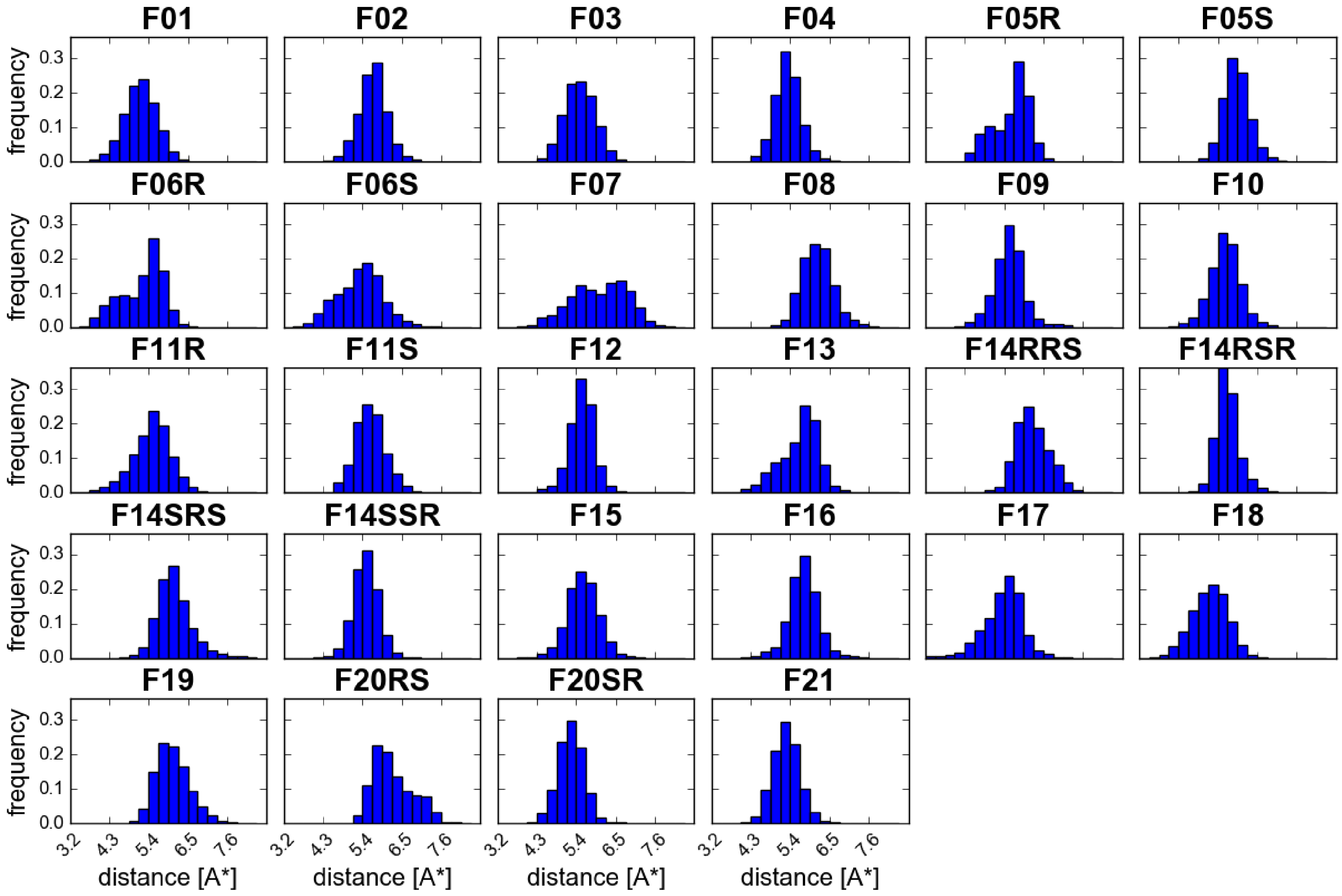


**Figure SM-DIS-3.** Distributions of the distance between Cgamma of D147 and phenolic O of Y326.
*Data are collected from last 10ns of production in 3 replicas.*

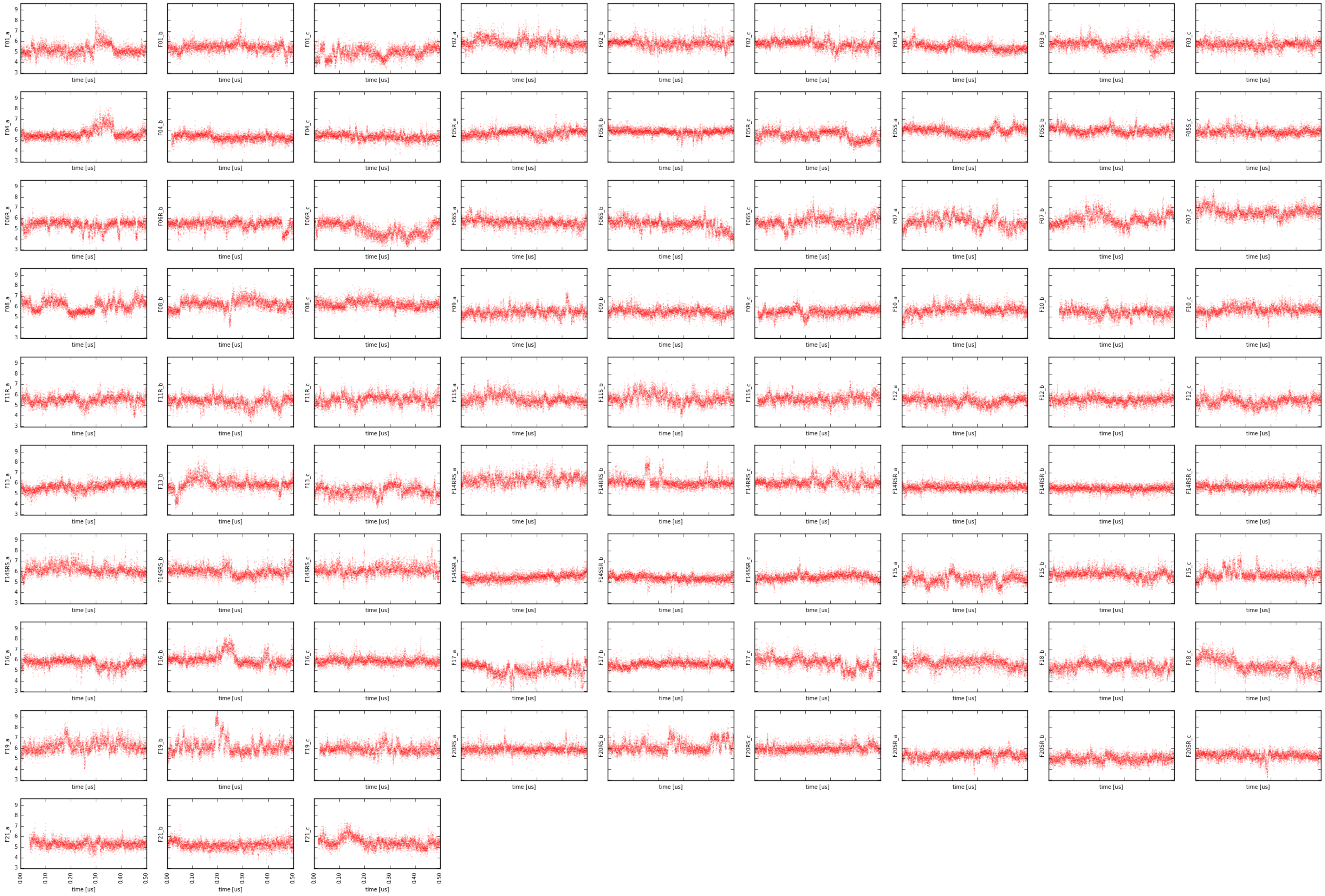

**Figure SM-DIS-4.** Time evolution of the distance between Cgamma of D147 and phenolic O of Y326.
*Y-values are given in angstroms.*

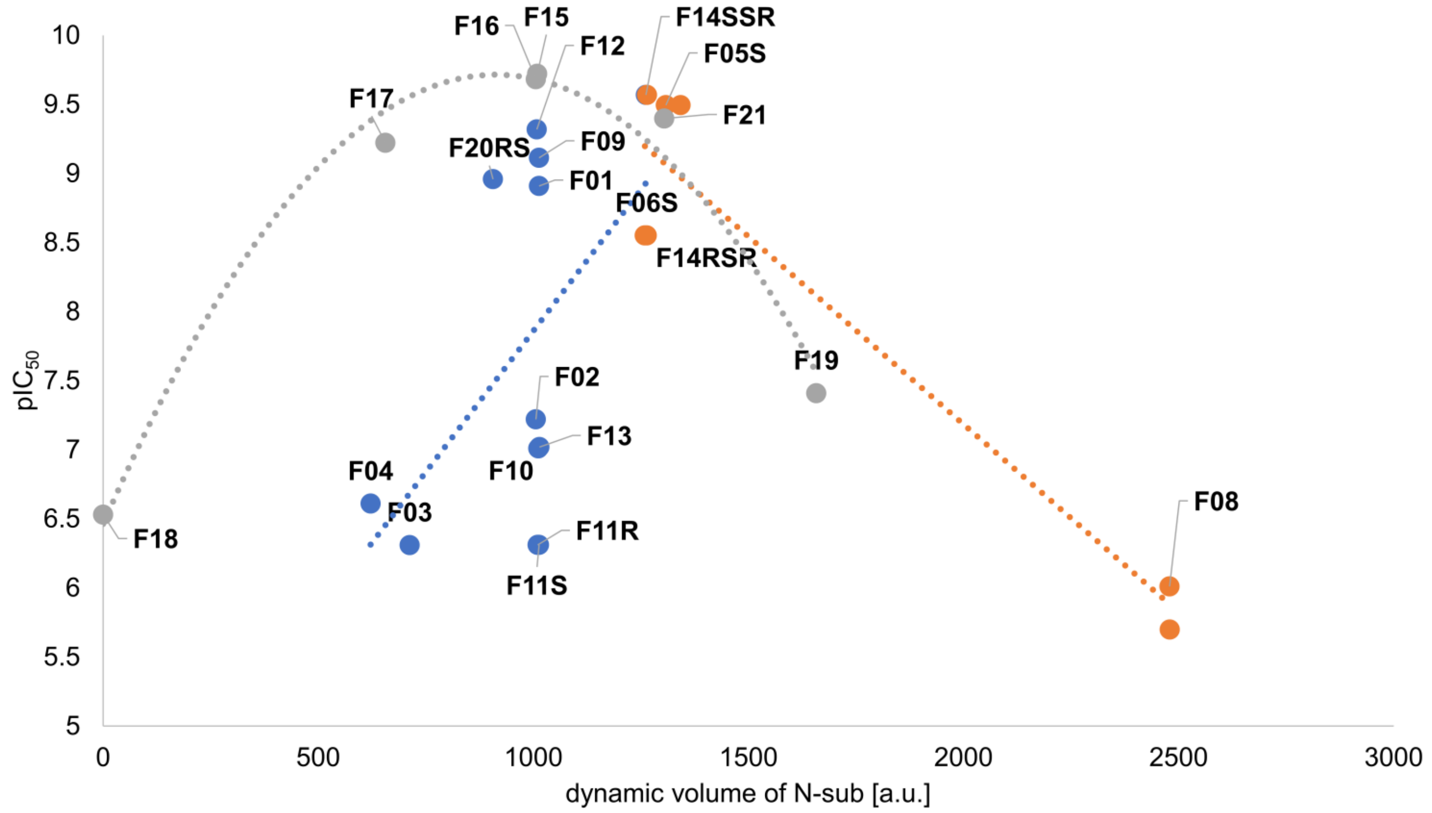


**Figure SM-VOL-1.** pIC50 values plotted against the dynamic volume of N-substituent for all compounds except for F14RRS, F14SRS and F20RS. *Volume data are collected from last 10ns of production in 3 replicas.*

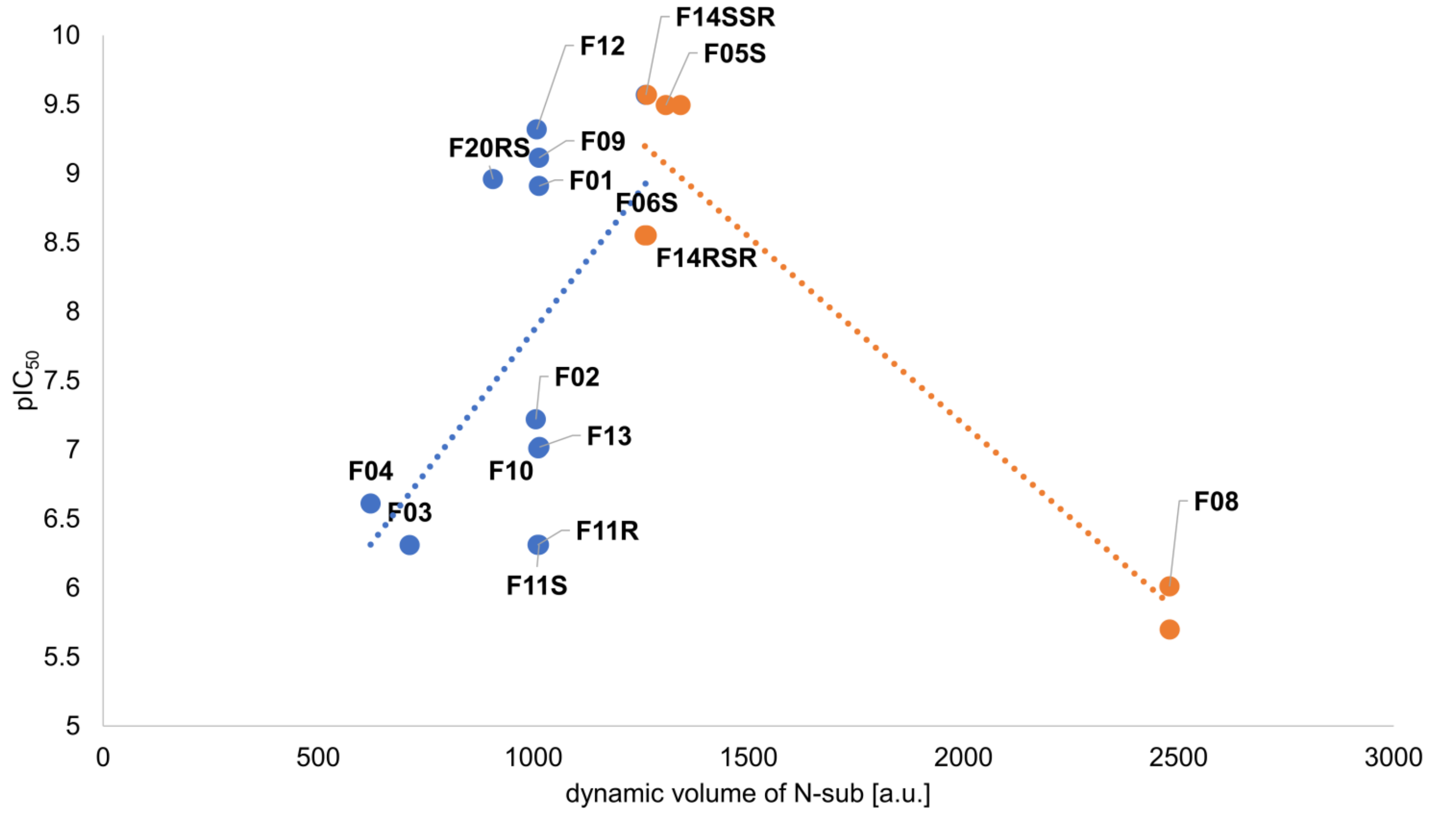


**Figure SM-VOL-2.** pIC50 values plotted against the dynamic volume of N-substituent.
*4-axially substituted derivatives not shown. Neither F14RRS, F14SRS and F20RS. Volume data are collected from last 10ns of production in 3 replicas.*

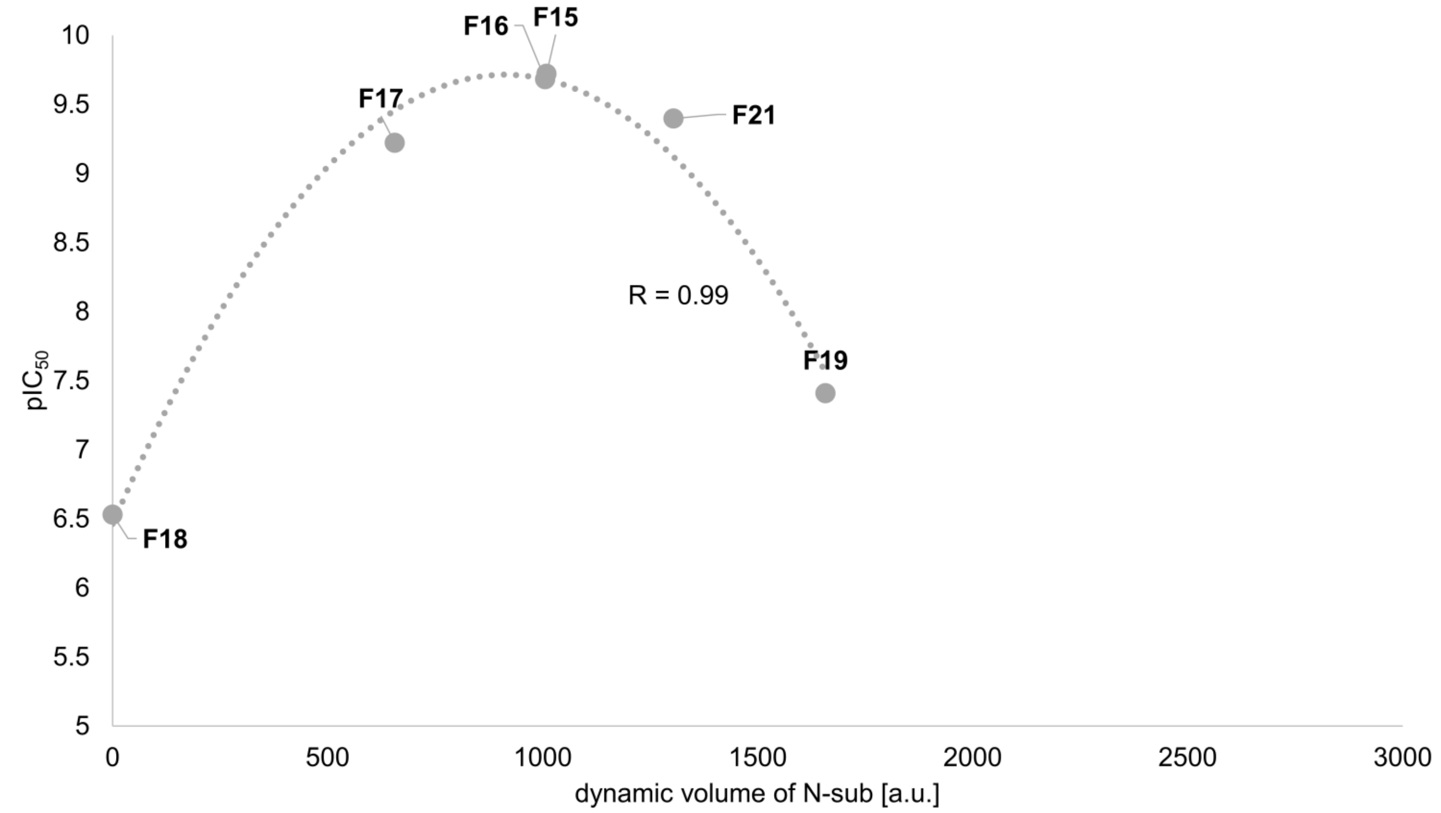


**Figure SM-VOL-3.** pIC50 values plotted against the dynamic volume of N-substituent for 4-axially substituted derivatives. *Volume data are collected from last 10ns of production in 3 replicas.*

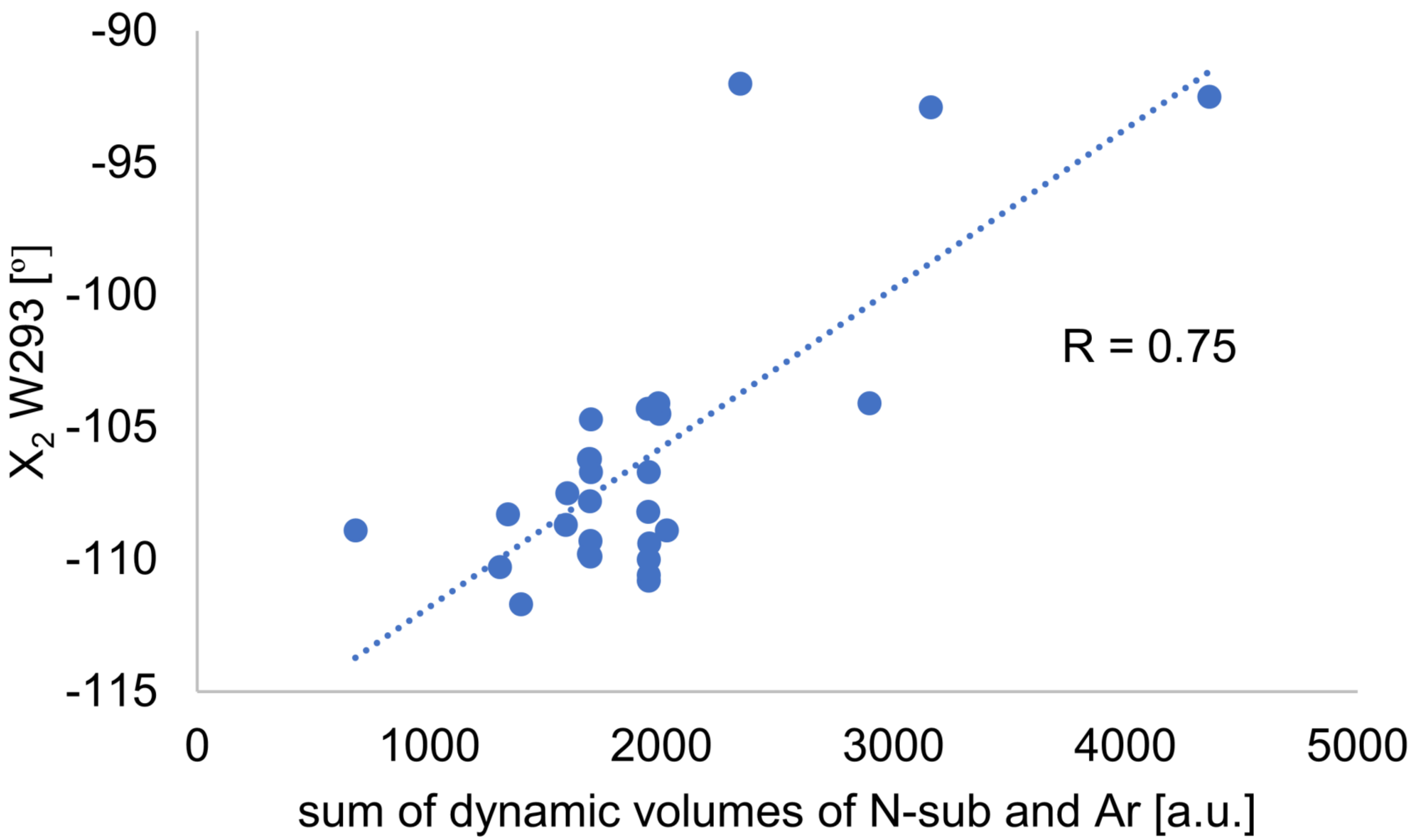


**Figure SM-VOL-4.** Correlation of mean X2 W293 dihedral measures with the sum of dynamic volumes of N-substituent and anilide's aromatic. *R = 0.75, n = 28. Volume data are collected from last 10ns of production in 3 replicas. Mean dihedrals are calculated from last 10ns of production in 3 replicas.*

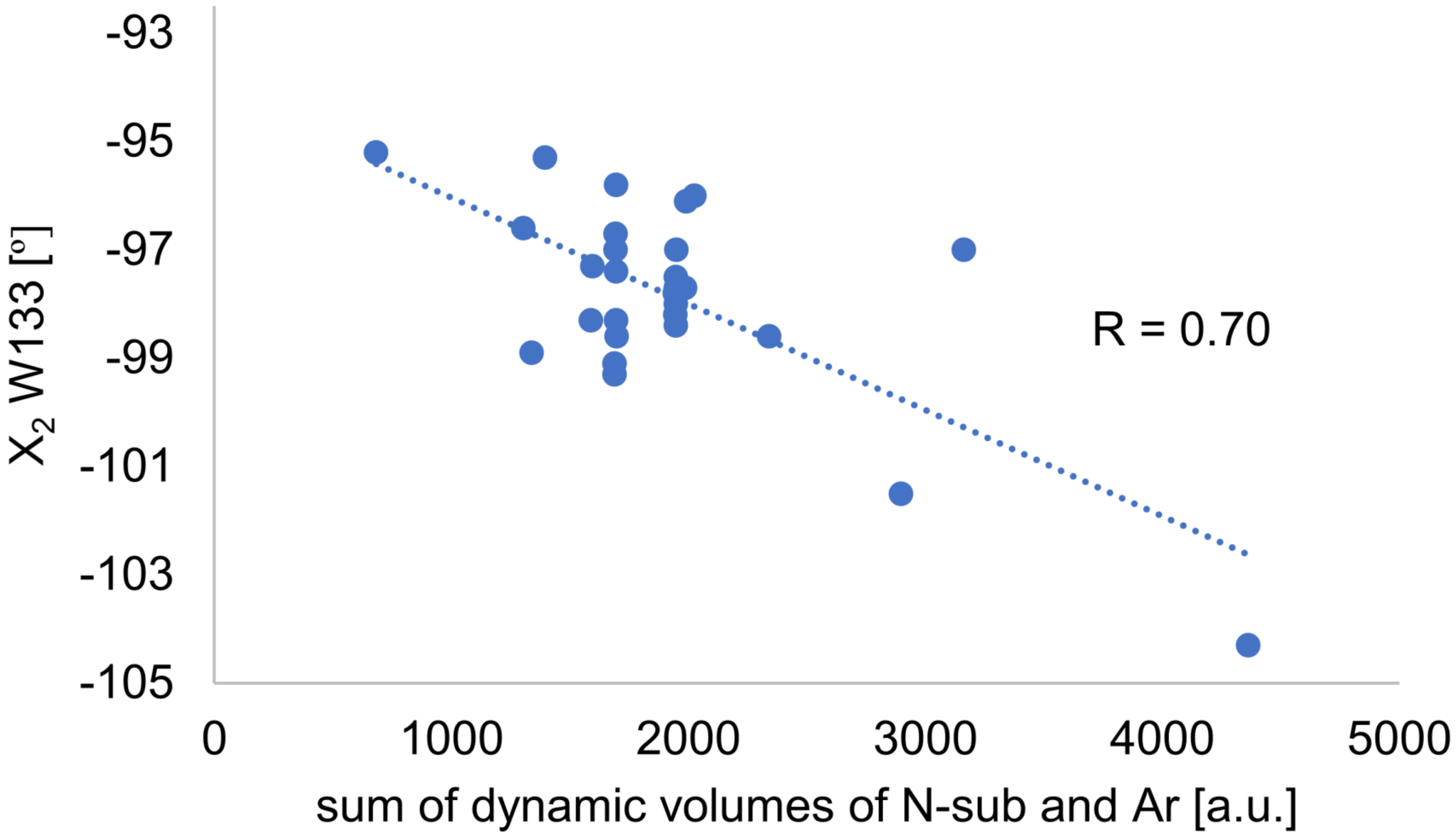


**Figure SM-VOL-5.** Correlation of mean X2 W133 dihedral measures with the sum of dynamic volumes of N-substituent and anilide's aromatic. *R = 0.70, n = 28. Volume data are collected from last 10ns of production in 3 replicas. Mean dihedrals are calculated from last 10ns of production in 3 replicas.*

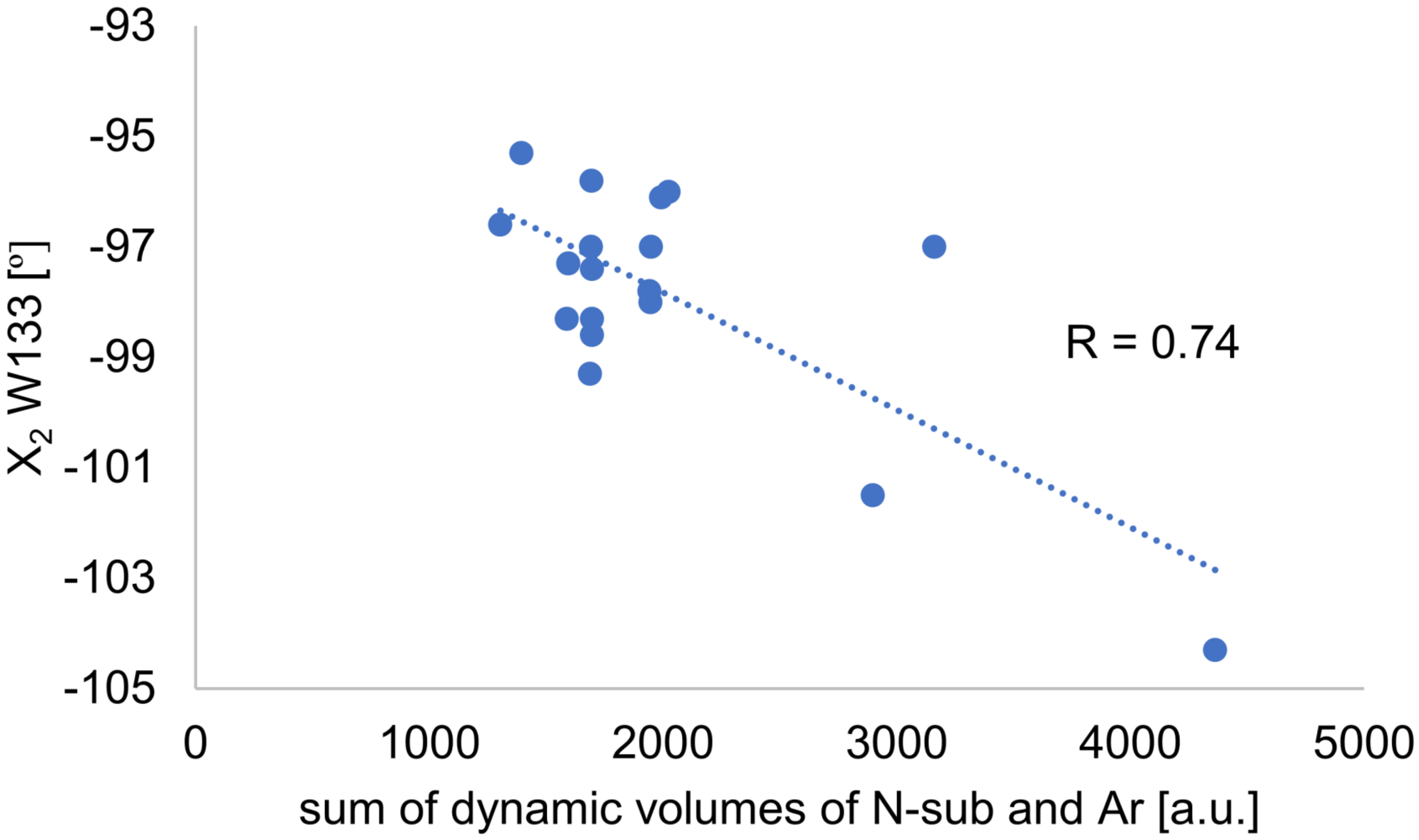


**Figure SM-VOL-6.** Correlation of mean X2 W133 dihedral measures with the sum of dynamic volumes of N-substituent and anilide's aromatic. *R = 0.74, n = 18. Volume data are collected from last 10ns of production in 3 replicas. Mean dihedrals are calculated from last 10ns of production in 3 replicas.*

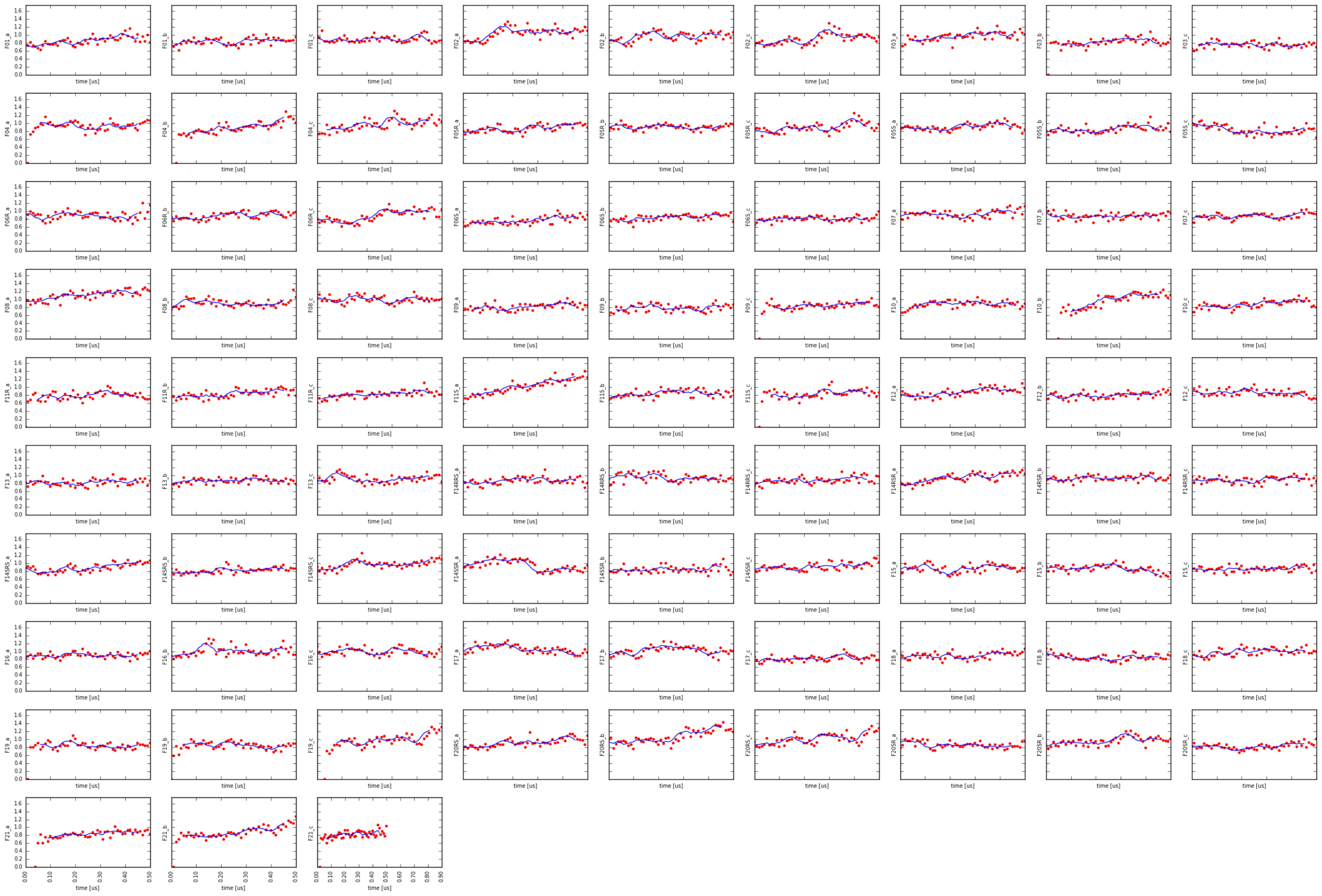

**Figure SM-RMS-1.** Root mean square deviation of receptor protein backbone over time.

*Y-values are given in angstroms. For RMSD calculation taken are only backbone atoms of residues 72-92 (H1), 104-128 (H2), 139-169 (H3), 185-204 (H4), 227-260 (H5), 275-304 (H6), 314-338 (H7).*

**Table SM-RMS-1.** Root mean square fluctuations of receptor protein backbone

*Calculated as a mean RMSD over last 10 ns of simulations in 3 replicas. For RMSD calculation taken are only backbone atoms of residues 72-92 (H1), 104-128 (H2), 139-169 (H3), 185-204 (H4), 227-260 (H5), 275-304 (H6), 314-338 (H7).*

| **derivative** | **replica** | | | | | | | |
|---|---|---|---|---|---|---|---|---|
| | **a** | | **b** | | **c** | | | |
| | **mean** | **sem** | **mean** | **sem** | **mean** | **sem** | **MEAN** | **SEM** |
| **F01** | 0.95 | 0.04 | 0.87 | 0.01 | 0.93 | 0.04 | 0.92 | 0.02 |
| **F02** | 1.09 | 0.03 | 1.02 | 0.02 | 0.96 | 0.02 | 1.02 | 0.04 |
| **F03** | 1.01 | 0.03 | 0.85 | 0.03 | 0.76 | 0.01 | 0.87 | 0.07 |
| **F04** | 0.96 | 0.03 | 1.07 | 0.04 | 1.05 | 0.03 | 1.03 | 0.03 |
| **F05R** | 0.96 | 0.01 | 0.91 | 0.02 | 0.98 | 0.04 | 0.95 | 0.02 |
| **F05S** | 0.94 | 0.04 | 0.92 | 0.04 | 0.84 | 0.03 | 0.9 | 0.03 |
| **F06R** | 0.87 | 0.05 | 0.94 | 0.02 | 0.98 | 0.03 | 0.93 | 0.03 |
| **F06S** | 0.84 | 0.02 | 0.91 | 0.02 | 0.83 | 0.02 | 0.86 | 0.03 |
| **F07** | 1 | 0.03 | 0.9 | 0.02 | 0.94 | 0.02 | 0.95 | 0.03 |
| **F08** | 1.2 | 0.03 | 0.93 | 0.04 | 0.99 | 0.01 | 1.04 | 0.08 |
| **F09** | 0.88 | 0.03 | 0.82 | 0.03 | 0.91 | 0.02 | 0.87 | 0.03 |
| **F10** | 0.88 | 0.02 | 1.11 | 0.02 | 0.95 | 0.03 | 0.98 | 0.07 |
| **F11R** | 0.78 | 0.02 | 0.91 | 0.03 | 0.89 | 0.03 | 0.86 | 0.04 |
| **F11S** | 1.23 | 0.03 | 0.82 | 0.02 | 0.88 | 0.02 | 0.98 | 0.13 |
| **F12** | 0.93 | 0.03 | 0.85 | 0.02 | 0.8 | 0.02 | 0.86 | 0.04 |
| **F13** | 0.82 | 0.02 | 0.86 | 0.01 | 0.97 | 0.02 | 0.88 | 0.04 |
| **F14RRS** | 0.86 | 0.03 | 0.9 | 0.02 | 0.92 | 0.03 | 0.89 | 0.02 |
| **F14RSR** | 1.05 | 0.02 | 0.94 | 0.03 | 0.92 | 0.03 | 0.97 | 0.04 |
| **F14SRS** | 1.01 | 0.01 | 0.88 | 0.02 | 1.04 | 0.03 | 0.98 | 0.05 |
| **F14SSR** | 0.85 | 0.02 | 0.9 | 0.04 | 0.99 | 0.03 | 0.91 | 0.04 |
| **F15** | 0.89 | 0.02 | 0.77 | 0.03 | 0.91 | 0.02 | 0.86 | 0.04 |
| **F16** | 0.9 | 0.02 | 1.02 | 0.04 | 0.95 | 0.02 | 0.96 | 0.03 |
| **F17** | 0.96 | 0.02 | 0.96 | 0.03 | 0.82 | 0.02 | 0.91 | 0.05 |
| **F18** | 0.96 | 0.02 | 0.86 | 0.02 | 1.01 | 0.02 | 0.94 | 0.04 |
| **F19** | 0.83 | 0.02 | 0.81 | 0.03 | 1.11 | 0.05 | 0.92 | 0.1 |
| **F20RS** | 1.02 | 0.03 | 1.28 | 0.03 | 1.1 | 0.06 | 1.13 | 0.08 |
| **F20SR** | 0.83 | 0.02 | 1 | 0.02 | 0.89 | 0.02 | 0.91 | 0.05 |
| **F21** | 0.9 | 0.02 | 1.01 | 0.04 | 0.86 | 0.03 | 0.92 | 0.04 |

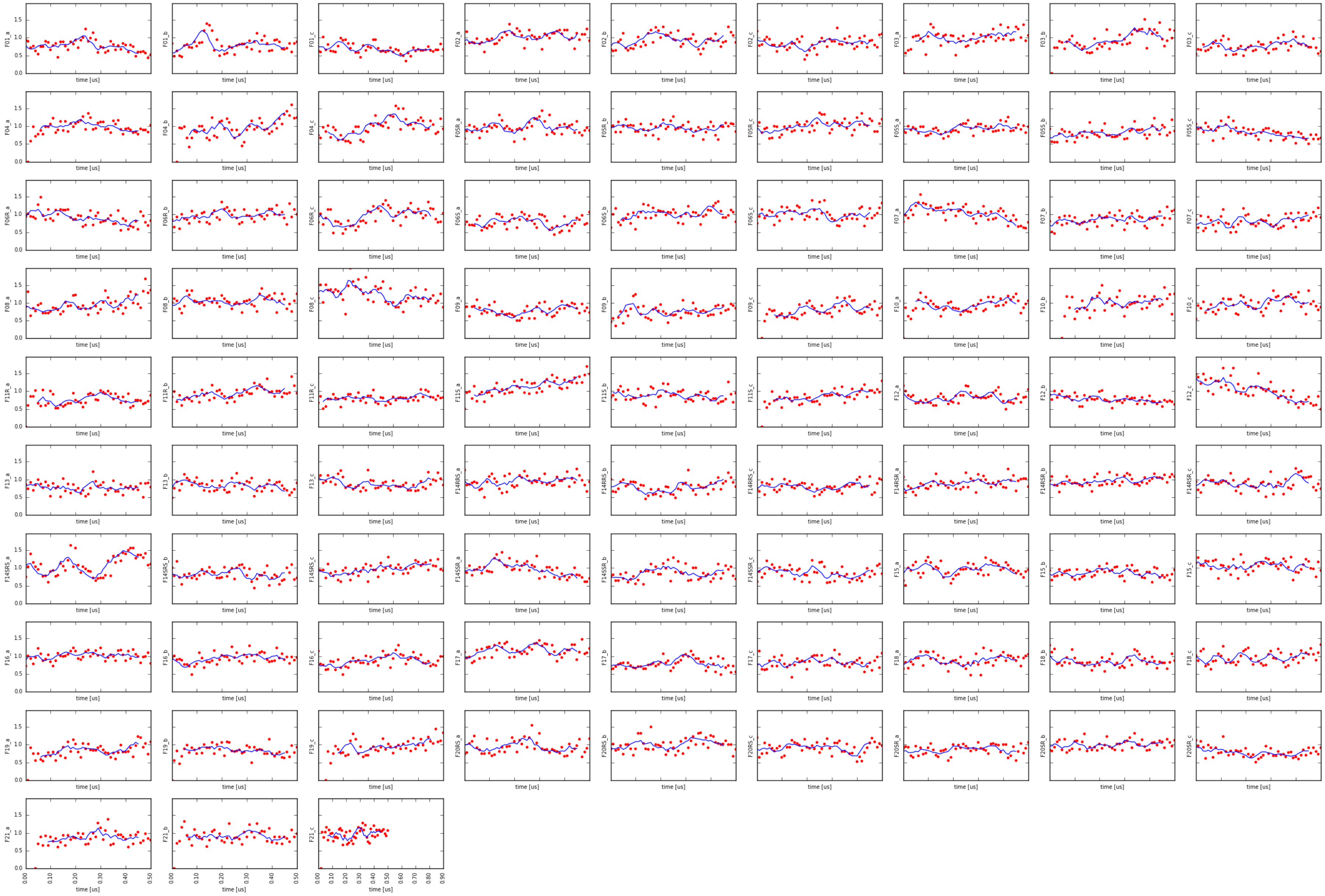

**Figure SM-RMS-2.** Root mean square deviation of TM1 helix backbone over time.

*Y-values are given in angstroms. For RMSD calculation taken are only backbone atoms of residues 72-92 (H1).*

**Table SM-RMS-2.** Root mean square fluctuations of TM1 helix backbone.

*Calculated as a mean RMSD over last 10 ns of simulations in 3 replicas. For RMSD calculation taken are only backbone atoms of residues 72-92 (H1).*

| derivative | replica | | | | | | | |
|---|---|---|---|---|---|---|---|---|
| | a | | b | | c | | | |
| | mean | sem | mean | sem | mean | sem | MEAN | SEM |
| F01 | 0.6 | 0.04 | 0.79 | 0.04 | 0.68 | 0.02 | 0.69 | 0.06 |
| F02 | 1.04 | 0.06 | 1 | 0.06 | 0.9 | 0.04 | 0.98 | 0.04 |
| F03 | 1.12 | 0.06 | 1.09 | 0.07 | 0.8 | 0.05 | 1 | 0.1 |
| F04 | 0.87 | 0.02 | 1.24 | 0.07 | 1.02 | 0.06 | 1.04 | 0.11 |
| F05R | 0.93 | 0.04 | 0.95 | 0.05 | 1.06 | 0.05 | 0.98 | 0.04 |
| F05S | 0.99 | 0.05 | 0.96 | 0.06 | 0.69 | 0.03 | 0.88 | 0.1 |
| F06R | 0.76 | 0.06 | 1.04 | 0.06 | 1 | 0.06 | 0.93 | 0.09 |
| F06S | 0.76 | 0.04 | 1.11 | 0.06 | 1.02 | 0.06 | 0.96 | 0.1 |
| F07 | 0.77 | 0.05 | 0.95 | 0.05 | 0.97 | 0.04 | 0.9 | 0.06 |
| F08 | 1.21 | 0.08 | 1 | 0.07 | 1.12 | 0.05 | 1.11 | 0.06 |
| F09 | 0.89 | 0.03 | 0.85 | 0.04 | 0.82 | 0.04 | 0.85 | 0.02 |
| F10 | 1.04 | 0.06 | 1.07 | 0.07 | 0.99 | 0.07 | 1.03 | 0.02 |
| F11R | 0.7 | 0.04 | 1.01 | 0.06 | 0.89 | 0.04 | 0.87 | 0.09 |
| F11S | 1.33 | 0.06 | 0.81 | 0.06 | 1.01 | 0.03 | 1.05 | 0.15 |
| F12 | 0.74 | 0.04 | 0.69 | 0.02 | 0.69 | 0.04 | 0.71 | 0.02 |
| F13 | 0.78 | 0.04 | 0.77 | 0.04 | 0.97 | 0.05 | 0.84 | 0.07 |
| F14RRS | 0.99 | 0.07 | 0.94 | 0.05 | 0.85 | 0.06 | 0.93 | 0.04 |
| F14RSR | 0.99 | 0.06 | 1.07 | 0.02 | 1.01 | 0.06 | 1.02 | 0.02 |
| F14SRS | 1.38 | 0.05 | 0.78 | 0.06 | 1.09 | 0.03 | 1.08 | 0.17 |
| F14SSR | 0.75 | 0.04 | 0.89 | 0.05 | 0.87 | 0.05 | 0.84 | 0.04 |
| F15 | 0.93 | 0.04 | 0.79 | 0.05 | 1.03 | 0.05 | 0.92 | 0.07 |
| F16 | 1.02 | 0.04 | 0.97 | 0.04 | 0.85 | 0.04 | 0.95 | 0.05 |
| F17 | 1.19 | 0.06 | 0.72 | 0.06 | 0.78 | 0.05 | 0.9 | 0.15 |
| F18 | 0.93 | 0.03 | 0.84 | 0.04 | 1.04 | 0.04 | 0.94 | 0.06 |
| F19 | 0.94 | 0.06 | 0.75 | 0.04 | 1.1 | 0.06 | 0.93 | 0.1 |
| F20RS | 0.87 | 0.05 | 1.02 | 0.05 | 0.91 | 0.07 | 0.93 | 0.04 |
| F20SR | 0.81 | 0.06 | 1.04 | 0.03 | 0.78 | 0.03 | 0.88 | 0.08 |
| F21 | 0.84 | 0.05 | 0.85 | 0.04 | 1.01 | 0.03 | 0.9 | 0.06 |

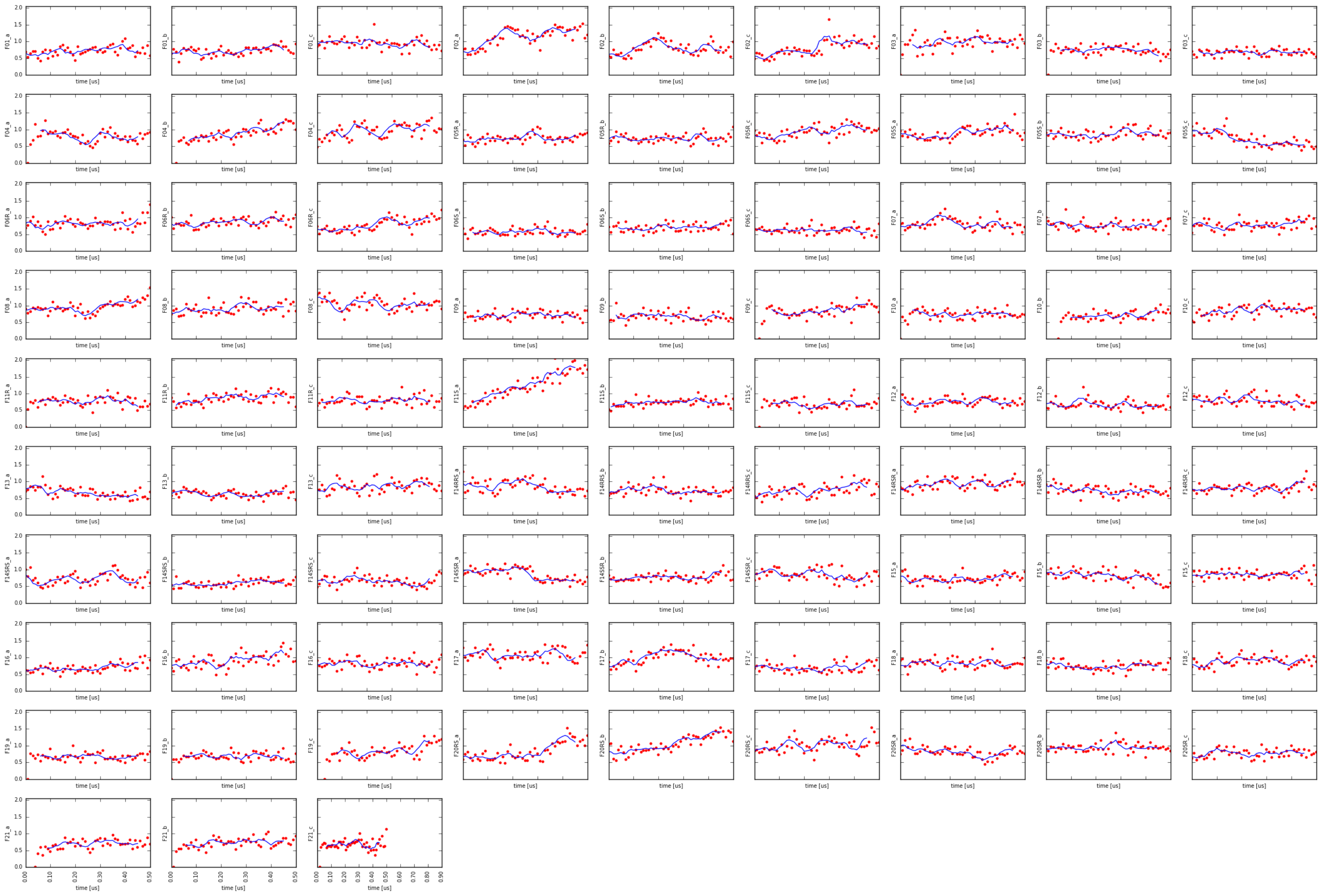


**Figure SM-RMS-3.** Root mean square deviation of TM2 helix backbone over time.

*Y-values are given in angstroms. For RMSD calculation taken are only backbone atoms of residues 104-128 (H2).*

**Table SM-RMS-3.** Root mean square fluctuations of TM2 helix backbone.
*Calculated as a mean RMSD over last 10 ns of simulations in 3 replicas. For RMSD calculation taken are only backbone atoms of residues 104-128 (H2).*

| **derivative** | **replica** | | | | | | | |
|---|---|---|---|---|---|---|---|---|
| | **a** | | **b** | | **c** | | | |
| | **mean** | **sem** | **mean** | **sem** | **mean** | **sem** | **MEAN** | **SEM** |
| **F01** | 0.74 | 0.04 | 0.82 | 0.04 | 0.92 | 0.05 | 0.83 | 0.05 |
| **F02** | 1.3 | 0.05 | 0.79 | 0.06 | 0.95 | 0.04 | 1.01 | 0.15 |
| **F03** | 0.99 | 0.04 | 0.67 | 0.05 | 0.67 | 0.02 | 0.78 | 0.11 |
| **F04** | 0.74 | 0.04 | 1.13 | 0.05 | 1.06 | 0.07 | 0.98 | 0.12 |
| **F05R** | 0.77 | 0.03 | 0.7 | 0.04 | 1.03 | 0.03 | 0.83 | 0.1 |
| **F05S** | 1 | 0.07 | 0.89 | 0.04 | 0.56 | 0.04 | 0.82 | 0.13 |
| **F06R** | 0.85 | 0.07 | 0.94 | 0.04 | 0.95 | 0.03 | 0.91 | 0.03 |
| **F06S** | 0.54 | 0.02 | 0.76 | 0.03 | 0.56 | 0.04 | 0.62 | 0.07 |
| **F07** | 0.72 | 0.04 | 0.79 | 0.04 | 0.88 | 0.04 | 0.8 | 0.05 |
| **F08** | 1.15 | 0.04 | 0.98 | 0.05 | 1.05 | 0.03 | 1.06 | 0.05 |
| **F09** | 0.71 | 0.04 | 0.66 | 0.04 | 1.03 | 0.03 | 0.8 | 0.12 |
| **F10** | 0.74 | 0.04 | 0.81 | 0.04 | 0.88 | 0.03 | 0.81 | 0.04 |
| **F11R** | 0.72 | 0.05 | 0.93 | 0.05 | 0.81 | 0.06 | 0.82 | 0.06 |
| **F11S** | 1.74 | 0.05 | 0.72 | 0.04 | 0.65 | 0.02 | 1.04 | 0.35 |
| **F12** | 0.71 | 0.03 | 0.64 | 0.04 | 0.72 | 0.05 | 0.69 | 0.03 |
| **F13** | 0.56 | 0.05 | 0.69 | 0.03 | 0.91 | 0.04 | 0.72 | 0.1 |
| **F14RRS** | 0.71 | 0.03 | 0.68 | 0.03 | 0.87 | 0.06 | 0.75 | 0.06 |
| **F14RSR** | 0.99 | 0.04 | 0.7 | 0.03 | 0.94 | 0.06 | 0.88 | 0.09 |
| **F14SRS** | 0.66 | 0.05 | 0.62 | 0.02 | 0.63 | 0.05 | 0.64 | 0.01 |
| **F14SSR** | 0.67 | 0.04 | 0.86 | 0.05 | 0.74 | 0.06 | 0.76 | 0.06 |
| **F15** | 0.83 | 0.03 | 0.65 | 0.06 | 0.89 | 0.06 | 0.79 | 0.07 |
| **F16** | 0.8 | 0.05 | 1.09 | 0.07 | 0.79 | 0.04 | 0.89 | 0.1 |
| **F17** | 1.03 | 0.05 | 0.96 | 0.04 | 0.78 | 0.05 | 0.92 | 0.07 |
| **F18** | 0.77 | 0.02 | 0.77 | 0.03 | 0.89 | 0.04 | 0.81 | 0.04 |
| **F19** | 0.68 | 0.02 | 0.64 | 0.04 | 1.05 | 0.08 | 0.79 | 0.13 |
| **F20RS** | 1.2 | 0.06 | 1.39 | 0.03 | 1.06 | 0.09 | 1.22 | 0.1 |
| **F20SR** | 0.83 | 0.03 | 0.95 | 0.02 | 0.8 | 0.03 | 0.86 | 0.05 |
| **F21** | 0.71 | 0.04 | 0.74 | 0.03 | 0.64 | 0.06 | 0.7 | 0.03 |

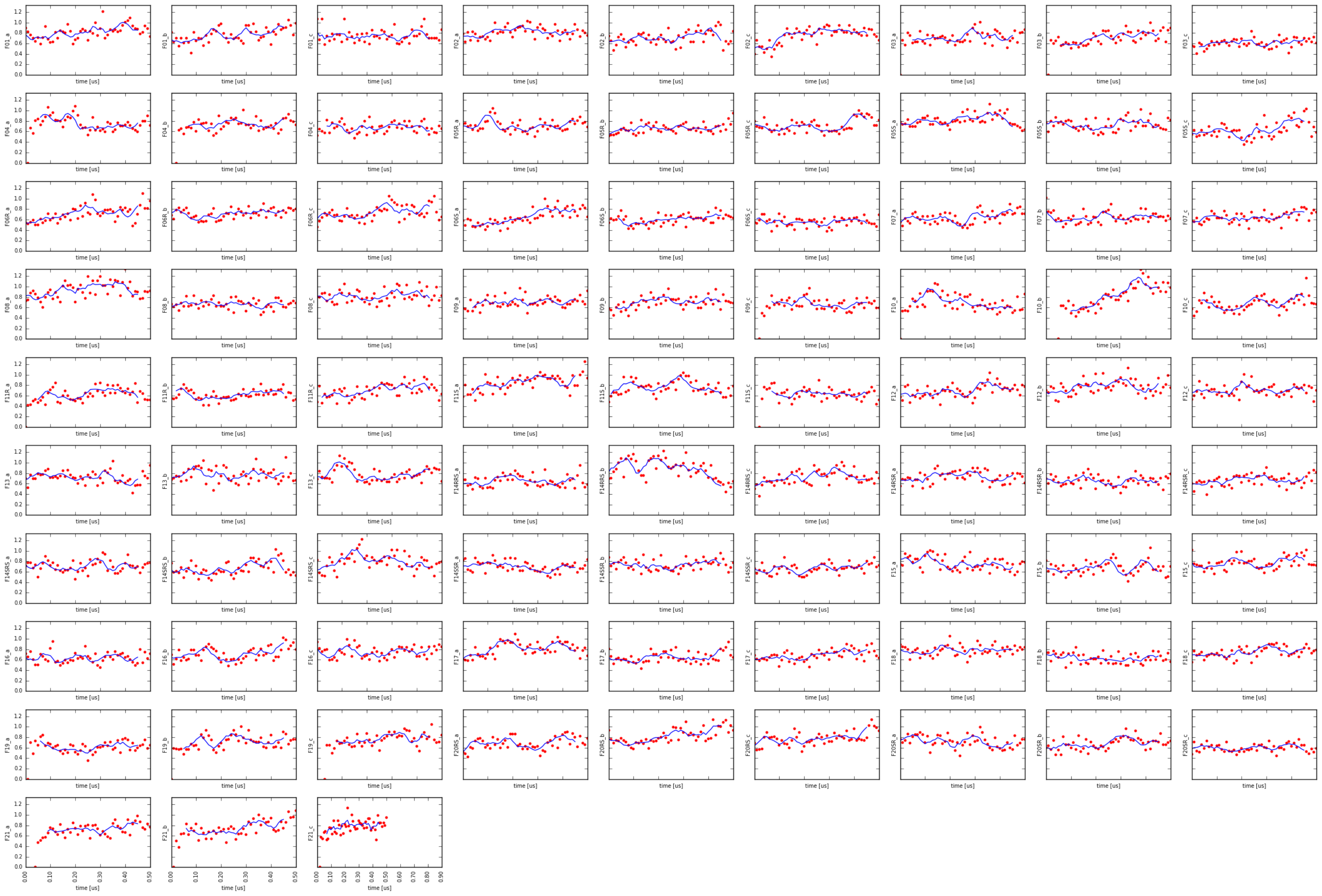


**Figure SM-RMS-4.** Root mean square deviation of TM3 helix backbone over time.

*Y-values are given in angstroms. For RMSD calculation taken are only backbone atoms of residues 139-169 (H3).*

**Table SM-RMS-4.** Root mean square fluctuations of TM3 helix backbone.
*Calculated as a mean RMSD over last 10 ns of simulations in 3 replicas. For RMSD calculation taken are only backbone atoms of residues 139-169 (H3).*

| derivative | replica | | | | | | | |
|---|---|---|---|---|---|---|---|---|
| | a | | b | | c | | | |
| | mean | sem | mean | sem | mean | sem | MEAN | SEM |
| F01 | 0.92 | 0.03 | 0.89 | 0.03 | 0.78 | 0.04 | 0.86 | 0.04 |
| F02 | 0.81 | 0.03 | 0.76 | 0.06 | 0.8 | 0.02 | 0.79 | 0.02 |
| F03 | 0.7 | 0.03 | 0.8 | 0.04 | 0.67 | 0.02 | 0.72 | 0.04 |
| F04 | 0.73 | 0.03 | 0.76 | 0.04 | 0.67 | 0.03 | 0.72 | 0.03 |
| F05R | 0.76 | 0.03 | 0.68 | 0.03 | 0.88 | 0.03 | 0.77 | 0.06 |
| F05S | 0.78 | 0.04 | 0.74 | 0.04 | 0.79 | 0.06 | 0.77 | 0.02 |
| F06R | 0.76 | 0.06 | 0.76 | 0.02 | 0.78 | 0.05 | 0.77 | 0.01 |
| F06S | 0.79 | 0.03 | 0.66 | 0.03 | 0.56 | 0.02 | 0.67 | 0.07 |
| F07 | 0.76 | 0.02 | 0.65 | 0.02 | 0.74 | 0.03 | 0.72 | 0.03 |
| F08 | 0.9 | 0.03 | 0.68 | 0.03 | 0.86 | 0.05 | 0.81 | 0.07 |
| F09 | 0.71 | 0.02 | 0.74 | 0.04 | 0.69 | 0.02 | 0.71 | 0.01 |
| F10 | 0.62 | 0.03 | 1 | 0.03 | 0.79 | 0.05 | 0.8 | 0.11 |
| F11R | 0.63 | 0.04 | 0.65 | 0.03 | 0.76 | 0.04 | 0.68 | 0.04 |
| F11S | 0.89 | 0.07 | 0.67 | 0.03 | 0.6 | 0.03 | 0.72 | 0.09 |
| F12 | 0.73 | 0.02 | 0.77 | 0.04 | 0.73 | 0.03 | 0.74 | 0.01 |
| F13 | 0.64 | 0.04 | 0.76 | 0.05 | 0.83 | 0.03 | 0.74 | 0.06 |
| F14RRS | 0.67 | 0.05 | 0.64 | 0.05 | 0.69 | 0.02 | 0.67 | 0.01 |
| F14RSR | 0.7 | 0.03 | 0.65 | 0.03 | 0.68 | 0.03 | 0.68 | 0.01 |
| F14SRS | 0.72 | 0.03 | 0.74 | 0.06 | 0.74 | 0.03 | 0.73 | 0.01 |
| F14SSR | 0.7 | 0.02 | 0.77 | 0.04 | 0.72 | 0.03 | 0.73 | 0.02 |
| F15 | 0.69 | 0.04 | 0.69 | 0.06 | 0.84 | 0.04 | 0.74 | 0.05 |
| F16 | 0.62 | 0.03 | 0.81 | 0.05 | 0.77 | 0.03 | 0.73 | 0.06 |
| F17 | 0.82 | 0.03 | 0.72 | 0.04 | 0.74 | 0.04 | 0.76 | 0.03 |
| F18 | 0.8 | 0.02 | 0.66 | 0.02 | 0.77 | 0.02 | 0.74 | 0.04 |
| F19 | 0.64 | 0.03 | 0.72 | 0.04 | 0.78 | 0.05 | 0.71 | 0.04 |
| F20RS | 0.77 | 0.04 | 0.99 | 0.04 | 0.88 | 0.05 | 0.88 | 0.06 |
| F20SR | 0.66 | 0.03 | 0.7 | 0.03 | 0.61 | 0.03 | 0.66 | 0.03 |
| F21 | 0.82 | 0.04 | 0.84 | 0.04 | 0.8 | 0.04 | 0.82 | 0.01 |

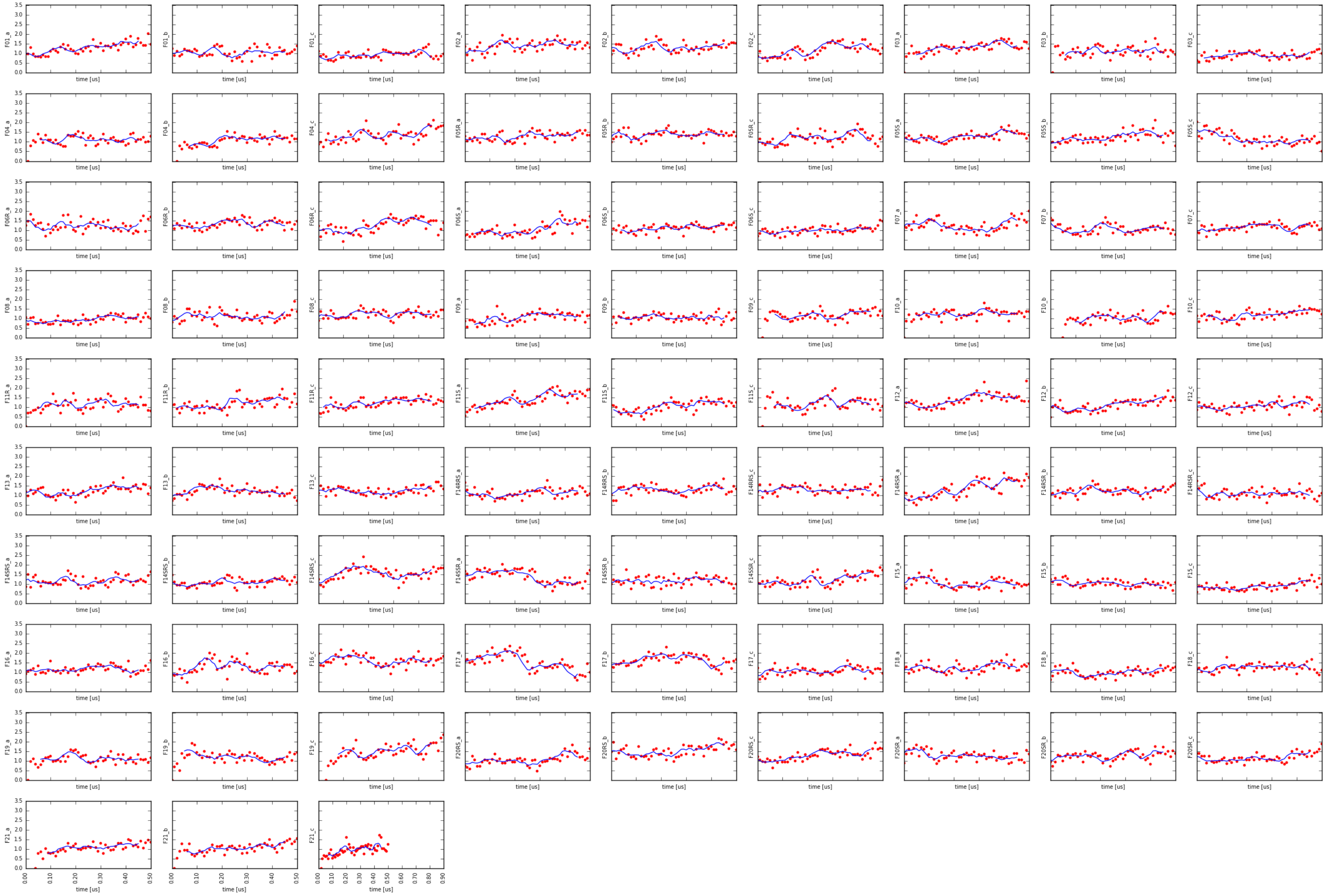

**Figure SM-RMS-5.** Root mean square deviation of TM4 helix backbone over time.

*Y-values are given in angstroms. For RMSD calculation taken are only backbone atoms of residues 185-204 (H4).*

**Table SM-RMS-5.** Root mean square fluctuations of TM4 helix backbone.
*Calculated as a mean RMSD over last 10 ns of simulations in 3 replicas. For RMSD calculation taken are only backbone atoms of residues 185-204 (H4).*

| derivative | replica | | | | | | | |
|---|---|---|---|---|---|---|---|---|
| | a | | b | | c | | | |
| | mean | sem | mean | sem | mean | sem | MEAN | SEM |
| **F01** | 1.59 | 0.08 | 1.07 | 0.04 | 1.01 | 0.09 | 1.22 | 0.18 |
| **F02** | 1.43 | 0.05 | 1.45 | 0.04 | 1.34 | 0.08 | 1.41 | 0.03 |
| **F03** | 1.46 | 0.06 | 1.18 | 0.09 | 0.96 | 0.06 | 1.2 | 0.14 |
| **F04** | 1.14 | 0.07 | 1.23 | 0.05 | 1.68 | 0.1 | 1.35 | 0.17 |
| **F05R** | 1.4 | 0.06 | 1.42 | 0.03 | 1.25 | 0.1 | 1.36 | 0.05 |
| **F05S** | 1.48 | 0.06 | 1.46 | 0.11 | 1.09 | 0.05 | 1.34 | 0.13 |
| **F06R** | 1.19 | 0.11 | 1.35 | 0.07 | 1.36 | 0.11 | 1.3 | 0.06 |
| **F06S** | 1.34 | 0.08 | 1.23 | 0.05 | 1.16 | 0.06 | 1.24 | 0.05 |
| **F07** | 1.53 | 0.09 | 1.11 | 0.05 | 1.24 | 0.04 | 1.29 | 0.12 |
| **F08** | 1.07 | 0.04 | 1.18 | 0.11 | 1.24 | 0.05 | 1.16 | 0.05 |
| **F09** | 1.14 | 0.06 | 1.08 | 0.09 | 1.39 | 0.05 | 1.2 | 0.09 |
| **F10** | 1.24 | 0.05 | 1.19 | 0.09 | 1.45 | 0.04 | 1.29 | 0.08 |
| **F11R** | 1.13 | 0.06 | 1.39 | 0.11 | 1.36 | 0.06 | 1.29 | 0.08 |
| **F11S** | 1.64 | 0.07 | 1.24 | 0.05 | 1.21 | 0.08 | 1.36 | 0.14 |
| **F12** | 1.55 | 0.11 | 1.44 | 0.07 | 1.19 | 0.08 | 1.39 | 0.11 |
| **F13** | 1.4 | 0.06 | 1.13 | 0.05 | 1.45 | 0.07 | 1.33 | 0.1 |
| **F14RRS** | 1.2 | 0.06 | 1.43 | 0.08 | 1.28 | 0.07 | 1.3 | 0.07 |
| **F14RSR** | 1.82 | 0.06 | 1.34 | 0.04 | 1.09 | 0.1 | 1.42 | 0.21 |
| **F14SRS** | 1.2 | 0.06 | 1.23 | 0.04 | 1.62 | 0.07 | 1.35 | 0.14 |
| **F14SSR** | 1.09 | 0.08 | 1.17 | 0.07 | 1.54 | 0.07 | 1.27 | 0.14 |
| **F15** | 1 | 0.04 | 0.93 | 0.05 | 1.15 | 0.06 | 1.03 | 0.06 |
| **F16** | 1.1 | 0.08 | 1.29 | 0.06 | 1.59 | 0.05 | 1.33 | 0.14 |
| **F17** | 1.02 | 0.11 | 1.35 | 0.09 | 1.1 | 0.06 | 1.16 | 0.1 |
| **F18** | 1.38 | 0.09 | 1.13 | 0.06 | 1.25 | 0.09 | 1.25 | 0.07 |
| **F19** | 1.05 | 0.05 | 1.11 | 0.06 | 1.67 | 0.12 | 1.28 | 0.2 |
| **F20RS** | 1.33 | 0.08 | 1.84 | 0.07 | 1.45 | 0.09 | 1.54 | 0.15 |
| **F20SR** | 1.14 | 0.08 | 1.46 | 0.06 | 1.4 | 0.04 | 1.33 | 0.1 |
| **F21** | 1.31 | 0.06 | 1.25 | 0.07 | 1.14 | 0.1 | 1.23 | 0.05 |

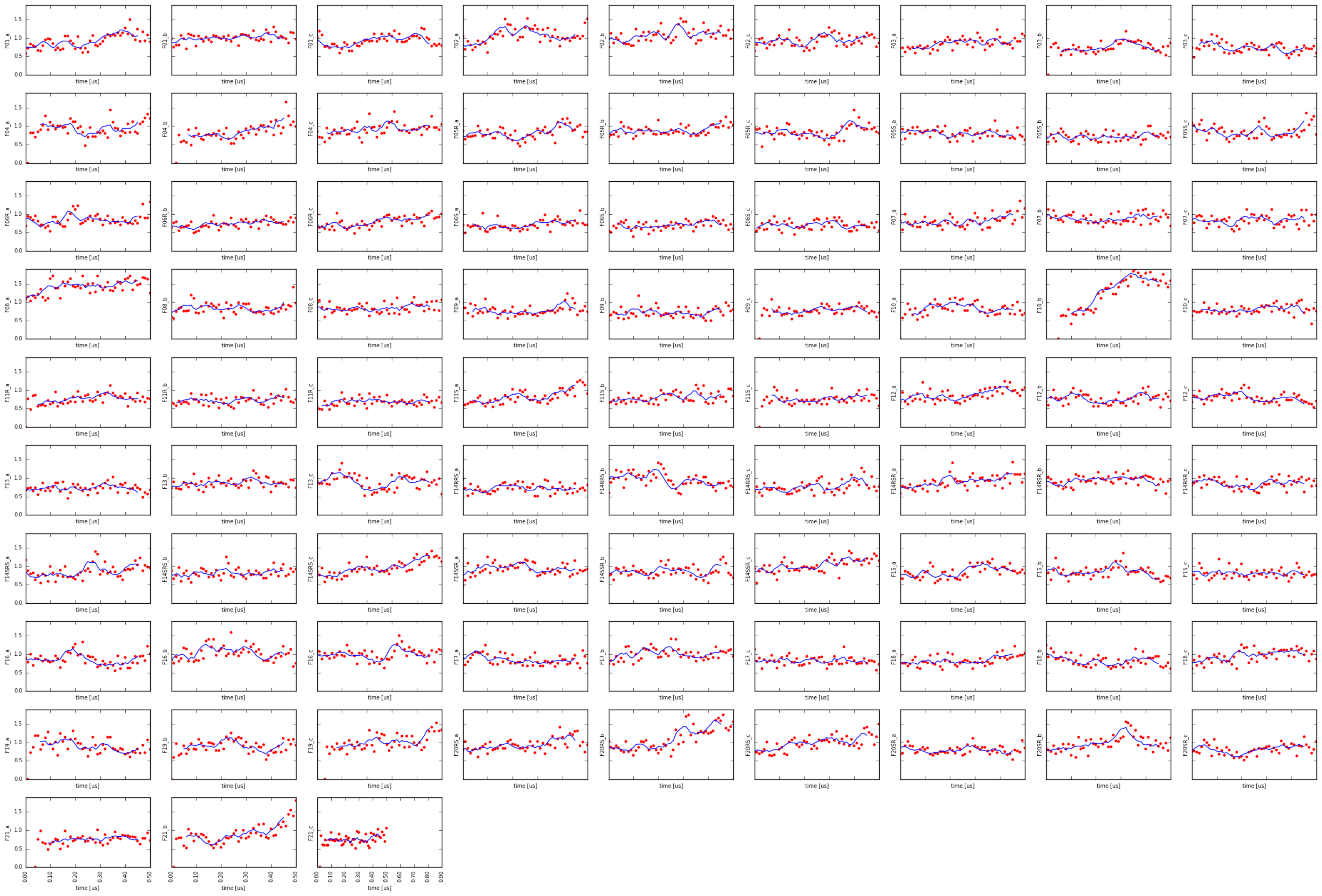


**Figure SM-RMS-6.** Root mean square deviation of TM5 helix backbone over time.

*Y-values are given in angstroms. For RMSD calculation taken are only backbone atoms of residues 227-260 (H5).*

**Table SM-RMS-6.** Root mean square fluctuations of TM5 helix backbone.
*Calculated as a mean RMSD over last 10 ns of simulations in 3 replicas. For RMSD calculation taken are only backbone atoms of residues 227-260 (H5).*

| derivative | replica | | | | | | | |
|---|---|---|---|---|---|---|---|---|
| | a | | b | | c | | | |
| | mean | sem | mean | sem | mean | sem | MEAN | SEM |
| **F01** | **1.11** | **0.06** | **1.08** | **0.04** | **0.97** | **0.06** | **1.05** | **0.04** |
| **F02** | 1.05 | 0.05 | 1.11 | 0.04 | 0.99 | 0.04 | 1.05 | 0.03 |
| **F03** | 0.9 | 0.04 | 0.71 | 0.04 | 0.7 | 0.03 | 0.77 | 0.07 |
| **F04** | 1.04 | 0.06 | 1.09 | 0.09 | 0.97 | 0.04 | 1.03 | 0.03 |
| **F05R** | 0.97 | 0.05 | 1.05 | 0.03 | 0.98 | 0.04 | 1 | 0.03 |
| **F05S** | 0.75 | 0.03 | 0.8 | 0.04 | 0.99 | 0.07 | 0.85 | 0.07 |
| **F06R** | 0.87 | 0.06 | 0.79 | 0.02 | 0.91 | 0.04 | 0.86 | 0.04 |
| **F06S** | 0.79 | 0.04 | 0.76 | 0.04 | 0.69 | 0.03 | 0.75 | 0.03 |
| **F07** | 0.98 | 0.06 | 0.93 | 0.04 | 0.87 | 0.06 | 0.93 | 0.03 |
| **F08** | 1.57 | 0.04 | 0.89 | 0.07 | 0.89 | 0.04 | 1.12 | 0.23 |
| **F09** | 0.92 | 0.06 | 0.78 | 0.05 | 0.81 | 0.04 | 0.84 | 0.04 |
| **F10** | 0.81 | 0.04 | 1.58 | 0.05 | 0.8 | 0.07 | 1.06 | 0.26 |
| **F11R** | 0.75 | 0.03 | 0.78 | 0.04 | 0.71 | 0.04 | 0.75 | 0.02 |
| **F11S** | 1.06 | 0.05 | 0.86 | 0.05 | 0.85 | 0.04 | 0.92 | 0.07 |
| **F12** | 1.02 | 0.04 | 0.82 | 0.05 | 0.72 | 0.04 | 0.85 | 0.09 |
| **F13** | 0.66 | 0.03 | 0.87 | 0.02 | 0.92 | 0.05 | 0.82 | 0.08 |
| **F14RRS** | 0.69 | 0.03 | 0.81 | 0.03 | 0.91 | 0.07 | 0.8 | 0.06 |
| **F14RSR** | 1.04 | 0.07 | 0.88 | 0.05 | 0.9 | 0.04 | 0.94 | 0.05 |
| **F14SRS** | 1.01 | 0.04 | 0.89 | 0.04 | 1.23 | 0.04 | 1.04 | 0.1 |
| **F14SSR** | 0.91 | 0.05 | 0.94 | 0.06 | 1.19 | 0.03 | 1.01 | 0.09 |
| **F15** | 0.92 | 0.03 | 0.73 | 0.04 | 0.84 | 0.04 | 0.83 | 0.06 |
| **F16** | 0.85 | 0.06 | 0.96 | 0.06 | 0.98 | 0.04 | 0.93 | 0.04 |
| **F17** | 0.84 | 0.05 | 1.05 | 0.03 | 0.77 | 0.03 | 0.89 | 0.08 |
| **F18** | 0.98 | 0.04 | 0.82 | 0.04 | 1.05 | 0.04 | 0.95 | 0.07 |
| **F19** | 0.78 | 0.05 | 0.9 | 0.05 | 1.17 | 0.09 | 0.95 | 0.12 |
| **F20RS** | 1.06 | 0.07 | 1.48 | 0.06 | 1.12 | 0.07 | 1.22 | 0.13 |
| **F20SR** | 0.76 | 0.05 | 0.92 | 0.04 | 0.87 | 0.05 | 0.85 | 0.05 |
| **F21** | 0.79 | 0.03 | 1.19 | 0.08 | 0.85 | 0.04 | 0.94 | 0.12 |

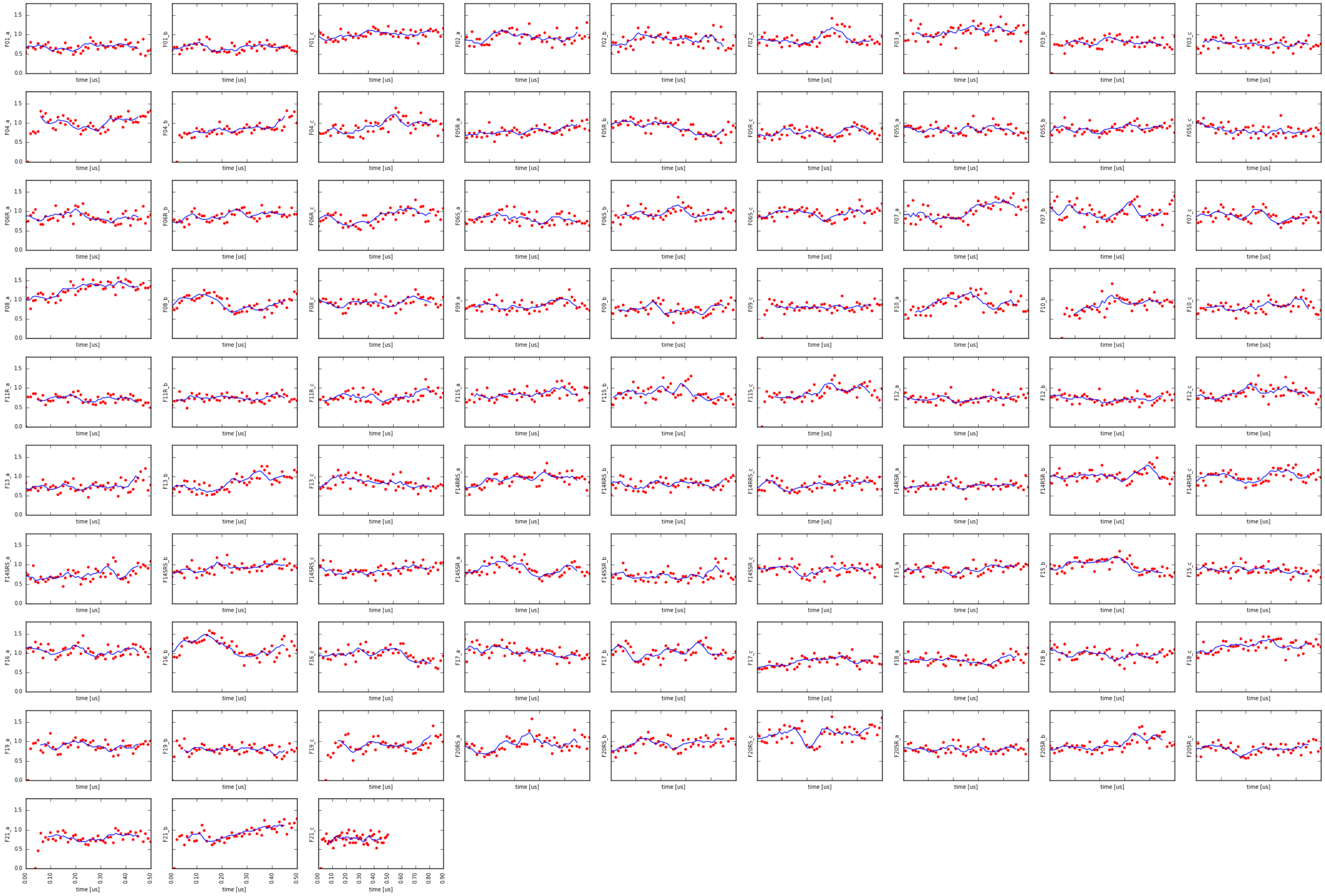

**Figure SM-RMS-7.** Root mean square deviation of TM6 helix backbone over time.
*Y-values are given in angstroms. For RMSD calculation taken are only backbone atoms of residues 275-304 (H6).*

**Table SM-RMS-7.** Root mean square fluctuations of TM6 helix backbone.

*Calculated as a mean RMSD over last 10 ns of simulations in 3 replicas. For RMSD calculation taken are only backbone atoms of residues 275-304 (H6).*

| derivative | replica | | | | | | | |
|---|---|---|---|---|---|---|---|---|
| | a | | b | | c | | | |
| | mean | sem | mean | sem | mean | sem | MEAN | SEM |
| F01 | 0.67 | 0.05 | 0.65 | 0.02 | 1.06 | 0.02 | 0.79 | 0.13 |
| F02 | 0.97 | 0.06 | 0.82 | 0.08 | 0.81 | 0.02 | 0.87 | 0.05 |
| F03 | 1.1 | 0.05 | 0.75 | 0.03 | 0.76 | 0.03 | 0.87 | 0.12 |
| F04 | 1.14 | 0.04 | 1.05 | 0.07 | 1 | 0.05 | 1.06 | 0.04 |
| F05R | 0.94 | 0.03 | 0.75 | 0.06 | 0.78 | 0.03 | 0.82 | 0.06 |
| F05S | 0.78 | 0.04 | 0.92 | 0.03 | 0.78 | 0.03 | 0.83 | 0.05 |
| F06R | 0.84 | 0.05 | 0.95 | 0.03 | 0.94 | 0.05 | 0.91 | 0.04 |
| F06S | 0.75 | 0.03 | 0.96 | 0.03 | 1.02 | 0.05 | 0.91 | 0.08 |
| F07 | 1.19 | 0.06 | 0.99 | 0.06 | 0.83 | 0.03 | 1 | 0.1 |
| F08 | 1.36 | 0.03 | 0.95 | 0.04 | 0.96 | 0.04 | 1.09 | 0.14 |
| F09 | 0.92 | 0.05 | 0.85 | 0.04 | 0.85 | 0.03 | 0.87 | 0.02 |
| F10 | 0.91 | 0.06 | 0.96 | 0.03 | 0.89 | 0.07 | 0.92 | 0.02 |
| F11R | 0.68 | 0.03 | 0.78 | 0.03 | 0.92 | 0.05 | 0.79 | 0.07 |
| F11S | 0.9 | 0.05 | 0.71 | 0.03 | 0.96 | 0.06 | 0.86 | 0.08 |
| F12 | 0.78 | 0.03 | 0.72 | 0.04 | 0.83 | 0.04 | 0.78 | 0.03 |
| F13 | 0.84 | 0.08 | 0.98 | 0.03 | 0.75 | 0.03 | 0.86 | 0.07 |
| F14RRS | 0.95 | 0.05 | 0.82 | 0.05 | 0.84 | 0.04 | 0.87 | 0.04 |
| F14RSR | 0.79 | 0.04 | 1.07 | 0.07 | 1 | 0.04 | 0.95 | 0.08 |
| F14SRS | 0.86 | 0.05 | 1 | 0.03 | 0.94 | 0.04 | 0.93 | 0.04 |
| F14SSR | 0.91 | 0.04 | 0.85 | 0.07 | 0.9 | 0.04 | 0.89 | 0.02 |
| F15 | 0.96 | 0.02 | 0.81 | 0.03 | 0.79 | 0.03 | 0.85 | 0.05 |
| F16 | 1.08 | 0.04 | 1.11 | 0.08 | 0.76 | 0.03 | 0.98 | 0.11 |
| F17 | 0.92 | 0.03 | 0.93 | 0.03 | 0.78 | 0.03 | 0.88 | 0.05 |
| F18 | 0.9 | 0.04 | 0.96 | 0.03 | 1.18 | 0.05 | 1.01 | 0.09 |
| F19 | 0.88 | 0.03 | 0.72 | 0.04 | 1.03 | 0.06 | 0.88 | 0.09 |
| F20RS | 0.96 | 0.06 | 1.04 | 0.05 | 1.26 | 0.05 | 1.09 | 0.09 |
| F20SR | 0.82 | 0.02 | 1.07 | 0.05 | 0.88 | 0.05 | 0.92 | 0.08 |
| F21 | 0.85 | 0.03 | 1.09 | 0.04 | 0.73 | 0.04 | 0.89 | 0.11 |

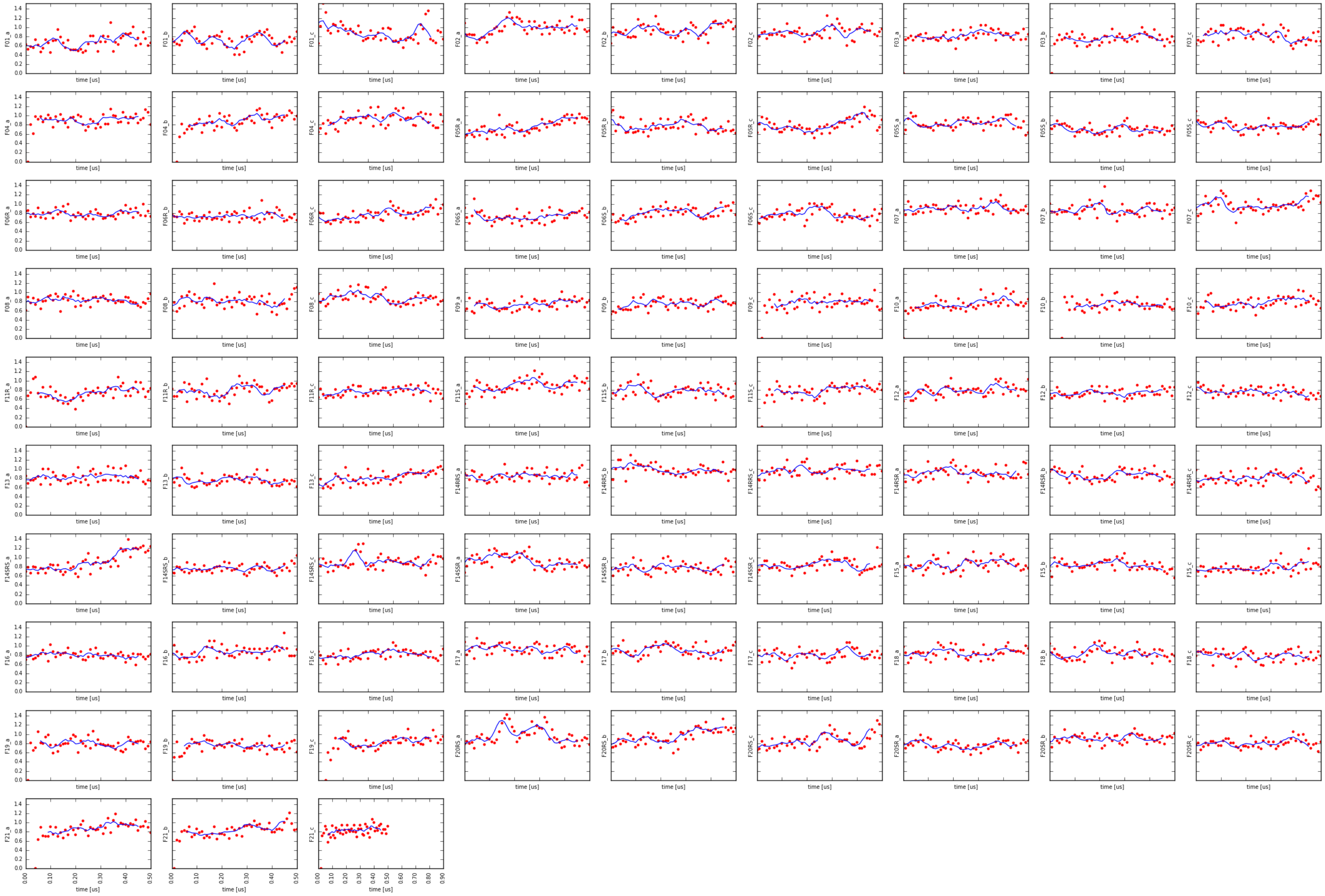

**Figure SM-RMS-8.** Root mean square deviation of TM7 helix backbone over time.

*Y-values are given in angstroms. For RMSD calculation taken are only backbone atoms of residues 314-338 (H7).*

**Table SM-RMS-8.** Root mean square fluctuations of TM7 helix backbone.

*Calculated as a mean RMSD over last 10 ns of simulations in 3 replicas. For RMSD calculation taken are only backbone atoms of residues 314-338 (H7).*

| **derivative** | **replica** | | | | | | | |
|---|---|---|---|---|---|---|---|---|
| | **a** | | **b** | | **c** | | | |
| | **mean** | **sem** | **mean** | **sem** | **mean** | **sem** | **MEAN** | **SEM** |
| **F01** | 0.78 | 0.05 | 0.66 | 0.03 | 0.96 | 0.08 | 0.8 | 0.09 |
| **F02** | 1.02 | 0.04 | 1.08 | 0.02 | 0.94 | 0.03 | 1.01 | 0.04 |
| **F03** | 0.82 | 0.03 | 0.76 | 0.03 | 0.75 | 0.03 | 0.78 | 0.02 |
| **F04** | 0.97 | 0.03 | 0.99 | 0.02 | 0.91 | 0.04 | 0.96 | 0.02 |
| **F05R** | 0.95 | 0.02 | 0.7 | 0.03 | 0.96 | 0.06 | 0.87 | 0.09 |
| **F05S** | 0.85 | 0.05 | 0.69 | 0.02 | 0.81 | 0.02 | 0.78 | 0.05 |
| **F06R** | 0.83 | 0.03 | 0.76 | 0.03 | 0.89 | 0.03 | 0.83 | 0.04 |
| **F06S** | 0.79 | 0.02 | 0.89 | 0.04 | 0.71 | 0.03 | 0.8 | 0.05 |
| **F07** | 0.88 | 0.02 | 0.87 | 0.03 | 1.09 | 0.05 | 0.95 | 0.07 |
| **F08** | 0.78 | 0.02 | 0.78 | 0.06 | 0.85 | 0.02 | 0.8 | 0.02 |
| **F09** | 0.8 | 0.02 | 0.8 | 0.03 | 0.8 | 0.04 | 0.8 | 0 |
| **F10** | 0.83 | 0.05 | 0.71 | 0.02 | 0.86 | 0.04 | 0.8 | 0.05 |
| **F11R** | 0.8 | 0.04 | 0.86 | 0.02 | 0.78 | 0.04 | 0.81 | 0.02 |
| **F11S** | 0.96 | 0.03 | 0.73 | 0.04 | 0.85 | 0.03 | 0.85 | 0.07 |
| **F12** | 0.83 | 0.03 | 0.76 | 0.03 | 0.69 | 0.02 | 0.76 | 0.04 |
| **F13** | 0.8 | 0.03 | 0.71 | 0.02 | 0.94 | 0.02 | 0.82 | 0.07 |
| **F14RRS** | 0.85 | 0.05 | 0.95 | 0.03 | 0.97 | 0.03 | 0.92 | 0.04 |
| **F14RSR** | 0.94 | 0.05 | 0.85 | 0.04 | 0.8 | 0.05 | 0.86 | 0.04 |
| **F14SRS** | 1.19 | 0.03 | 0.77 | 0.03 | 0.88 | 0.05 | 0.95 | 0.13 |
| **F14SSR** | 0.86 | 0.02 | 0.78 | 0.04 | 0.79 | 0.06 | 0.81 | 0.03 |
| **F15** | 0.85 | 0.03 | 0.76 | 0.03 | 0.86 | 0.05 | 0.82 | 0.03 |
| **F16** | 0.75 | 0.03 | 0.92 | 0.05 | 0.79 | 0.02 | 0.82 | 0.05 |
| **F17** | 0.91 | 0.04 | 0.86 | 0.03 | 0.78 | 0.02 | 0.85 | 0.04 |
| **F18** | 0.93 | 0.03 | 0.79 | 0.04 | 0.79 | 0.03 | 0.84 | 0.05 |
| **F19** | 0.82 | 0.02 | 0.74 | 0.03 | 0.88 | 0.03 | 0.81 | 0.04 |
| **F20RS** | 0.88 | 0.03 | 1.11 | 0.03 | 0.95 | 0.08 | 0.98 | 0.07 |
| **F20SR** | 0.79 | 0.03 | 0.89 | 0.02 | 0.82 | 0.04 | 0.83 | 0.03 |
| **F21** | 0.94 | 0.02 | 0.94 | 0.05 | 0.85 | 0.02 | 0.91 | 0.03 |

| | H1 | H2 | H3 | H4 | H5 | H6 | H7 | prot |
|---|---|---|---|---|---|---|---|---|
| F01 | 0.69 | 0.83 | 0.86 | 1.22 | 1.05 | 0.79 | 0.80 | 0.92 |
| F02 | 0.98 | 1.01 | 0.79 | 1.41 | 1.05 | 0.87 | 1.01 | 1.02 |
| F03 | 1.00 | 0.78 | 0.72 | 1.20 | 0.77 | 0.87 | 0.78 | 0.87 |
| F04 | 1.04 | 0.98 | 0.72 | 1.35 | 1.03 | 1.06 | 0.96 | 1.03 |
| F05R | 0.98 | 0.83 | 0.77 | 1.36 | 1.00 | 0.82 | 0.87 | 0.95 |
| F05S | 0.88 | 0.82 | 0.77 | 1.34 | 0.85 | 0.83 | 0.78 | 0.90 |
| F06R | 0.93 | 0.91 | 0.77 | 1.30 | 0.86 | 0.91 | 0.83 | 0.93 |
| F06S | 0.96 | 0.62 | 0.67 | 1.24 | 0.75 | 0.91 | 0.80 | 0.86 |
| F07 | 0.90 | 0.80 | 0.72 | 1.29 | 0.93 | 1.00 | 0.95 | 0.95 |
| F08 | 1.11 | 1.06 | 0.81 | 1.16 | 1.12 | 1.09 | 0.80 | 1.04 |
| F09 | 0.85 | 0.80 | 0.71 | 1.20 | 0.84 | 0.87 | 0.80 | 0.87 |
| F10 | 1.03 | 0.81 | 0.80 | 1.29 | 1.06 | 0.92 | 0.80 | 0.98 |
| F11R | 0.87 | 0.82 | 0.68 | 1.29 | 0.75 | 0.79 | 0.81 | 0.86 |
| F11S | 1.05 | 1.04 | 0.72 | 1.36 | 0.92 | 0.86 | 0.85 | 0.98 |
| F12 | 0.71 | 0.69 | 0.74 | 1.39 | 0.85 | 0.78 | 0.76 | 0.86 |
| F13 | 0.84 | 0.72 | 0.74 | 1.33 | 0.82 | 0.86 | 0.82 | 0.88 |
| | | | | | | | | |
| F14RSR | 1.02 | 0.88 | 0.68 | 1.42 | 0.94 | 0.95 | 0.86 | 0.97 |
| | | | | | | | | |
| F14SSR | 0.84 | 0.76 | 0.73 | 1.27 | 1.01 | 0.89 | 0.81 | 0.91 |
| F15 | 0.92 | 0.79 | 0.74 | 1.03 | 0.83 | 0.85 | 0.82 | 0.86 |
| F16 | 0.95 | 0.89 | 0.73 | 1.33 | 0.93 | 0.98 | 0.82 | 0.96 |
| F17 | 0.90 | 0.92 | 0.76 | 1.16 | 0.89 | 0.88 | 0.85 | 0.91 |
| F18 | 0.94 | 0.81 | 0.74 | 1.25 | 0.95 | 1.01 | 0.84 | 0.94 |
| F19 | 0.93 | 0.79 | 0.71 | 1.28 | 0.95 | 0.88 | 0.81 | 0.92 |
| | | | | | | | | |
| F20SR | 0.88 | 0.86 | 0.66 | 1.33 | 0.85 | 0.92 | 0.83 | 0.91 |
| F21 | 0.90 | 0.70 | 0.82 | 1.23 | 0.94 | 0.89 | 0.91 | 0.92 |
| MEAN | 0.92 | 0.84 | 0.74 | 1.28 | 0.92 | 0.90 | 0.84 | 0.93 |
| STD | 0.10 | 0.11 | 0.05 | 0.09 | 0.10 | 0.08 | 0.06 | 0.05 |

**Figure SM-RMS-9.** RMSF mean values.

*Given are means from 3 simulations. H1-H7: backbone RMSF of helices. Prot: backbone RMSF of the protein. Colouring according to the order in a given category: from red (low values) to green (high values).*

**Table SM-DIH-1.** Summary of monitored dihedral angles.

| Residue | Dihedral | Value | Figures | Comment |
|---|---|---|---|---|
| **Interacting with (or close to) piperidine** | | | | |
| D147 | $X_1$ | *dominant cluster*: -166.9; *minor cluster* (**F01**, **F03**, **F04**, **F09**, **F11S**, **F17**): -92.2 | SM-DIH-1 and SM-DIH-2 | stable, transient switches to the minor cluster in several derivatives |
| | $X_2$ | several clusters | SM-DIH-3 and SM-DIH-4 | no clear preference among the derivatives |
| Y148 | $X_1$ | -72.5 | SM-DIH-5 and SM-DIH-6 | stable in all derivatives |
| | $X_2$ | several clusters | SM-DIH-7 and SM-DIH-8 | symmetrical rotamer exchanges |
| **Close to 4-axial substituent ("under" piperidine)** | | | | |
| W318 | $X_1$ | 175.9 | SM-DIH-9 and SM-DIH-10 | stable |
| | $X_2$ | -104.7 | SM-DIH-11 and SM-DIH-12 | stable, but differentiated with respect to derivatives, to the left given is the global mean value, however for separate sets of simulations the mean varies between -93.2 and -116.0 |
| H319 | $X_1$ | -67.8 | SM-DIH-13 and SM-DIH-14 | stable |
| | $X_2$ | 159.6 | SM-DIH-15 and SM-DIH-16 | in some derivatives also minor clusters |
| **Close to N-substituent** | | | | |
| M151 | $X_1$ | *dominant cluster*: 172.6<br>*minor cluster:* 78.7 | SM-DIH-17 and SM-DIH-18 | transient rotamer exchanges, without clear pattern with respect to derivatives |
| | $X_2$ | *dominant cluster*: -86.1<br>*minor clusters:* -177.2 and 85.9 | SM-DIH-19 and SM-DIH-20 | transient rotamer exchanges, without clear pattern with respect to derivatives |
| W293 | $X_1$ | -165.2 | SM-DIH-21 and SM-DIH-22 | stable |
| | $X_2$ | -106.3 (global mean) | 10, 11, SM-VOL-4, SM-DIH-23 and SM-DIH-24 | stable, but differentiated with respect to derivatives, a relationship between dihedral value and the volume sum of fentanyl's N-chain and anilide's aromatic |
| H297 | $X_1$ | -68.4 | SM-DIH-25 and SM-DIH-26 | stable |

| Residue | Dihedral | Value | Figures | Comment |
|---|---|---|---|---|
| | $X_2$ | 103.8 | SM-DIH-27 and SM-DIH-28 | stable |
| Y326 | $X_1$ | -67.6 | SM-DIH-29 and SM-DIH-30 | stable |
| | $X_2$ | -62.3<br>in **F03** and **F20RS** another cluster: 92.6 | SM-DIH-31 and SM-DIH-32 | stable |
| **Close to anilide's aromatic** | | | | |
| W133 | $X_1$ | 179.1 | SM-DIH-33 and SM-DIH-34 | stable |
| | $X_2$ | -97.8 (global mean) | SM-VOL-5, SM-VOL-6, SM-DIH-35 and SM-DIH-36 | stable, but differentiated with respect to derivatives, a relationship between dihedral value and the volume sum of fentanyl's N-chain and anilide's aromatic |
| **Outside the binding pocket** | | | | |
| Y336 | $X_1$ | -68.3 | SM-DIH-41 and SM-DIH-42 | stable |
| | $X_2$ | -76.6; in **F01** and **F13** another cluster: 111.4 | SM-DIH-43 and SM-DIH-44 | stable |

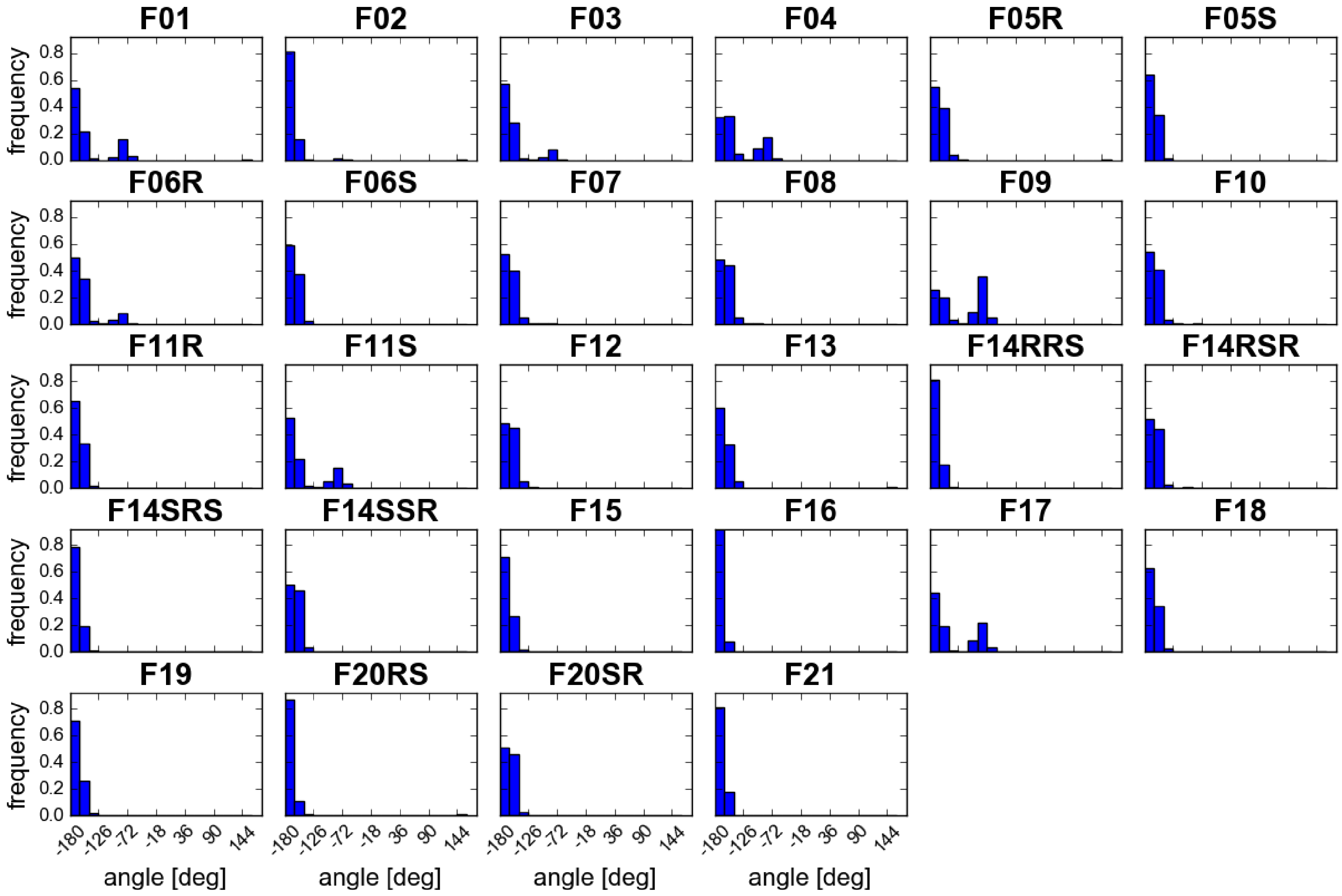


**Figure SM-DIH-1.** Distributions of X1 dihedral angle values of D147.
*Data are collected from last 10ns of production in 3 replicas.*

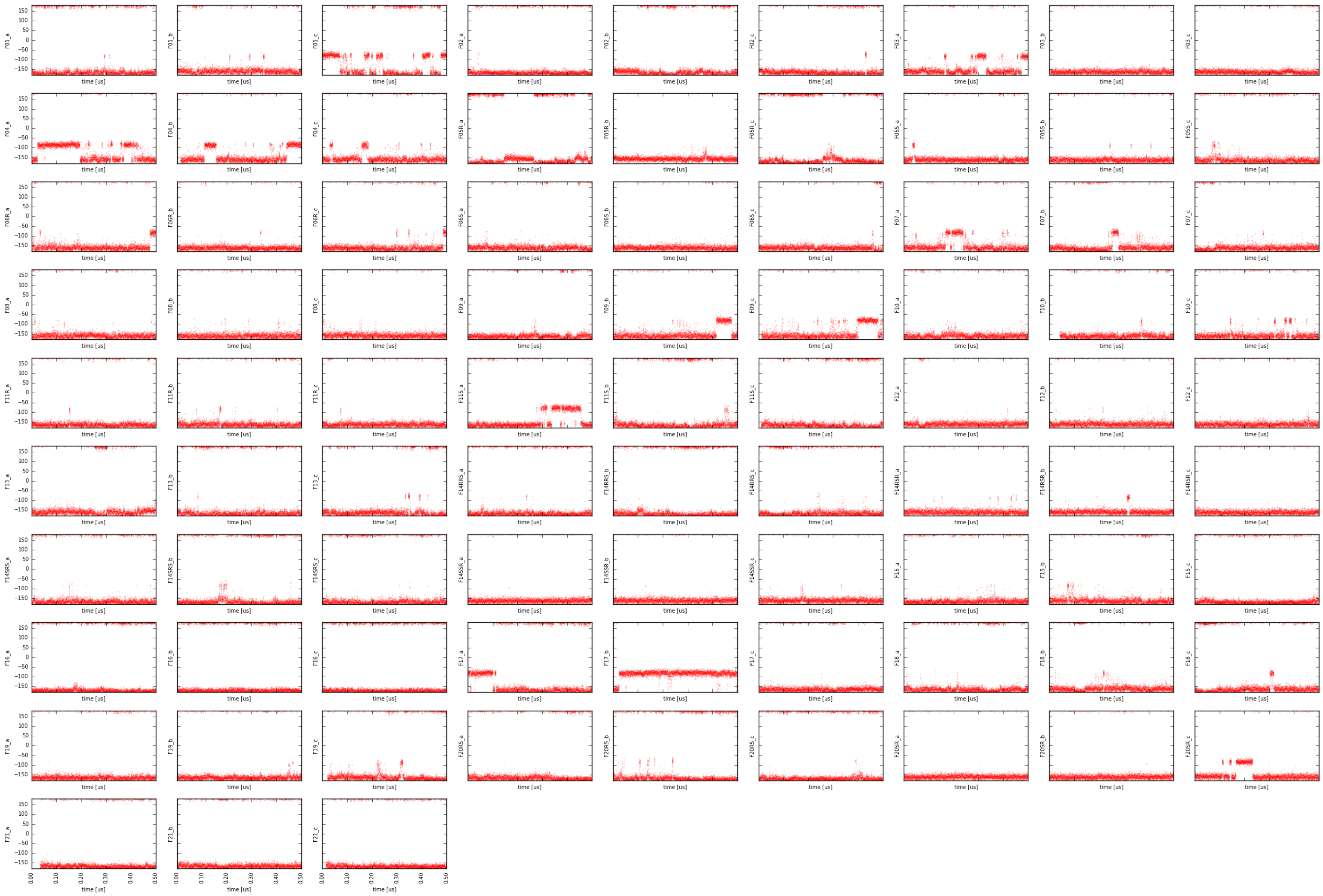


**Figure SM-DIH-2.** Time evolution of X1 dihedral angle of D147.
*Y-values are given in degrees.*

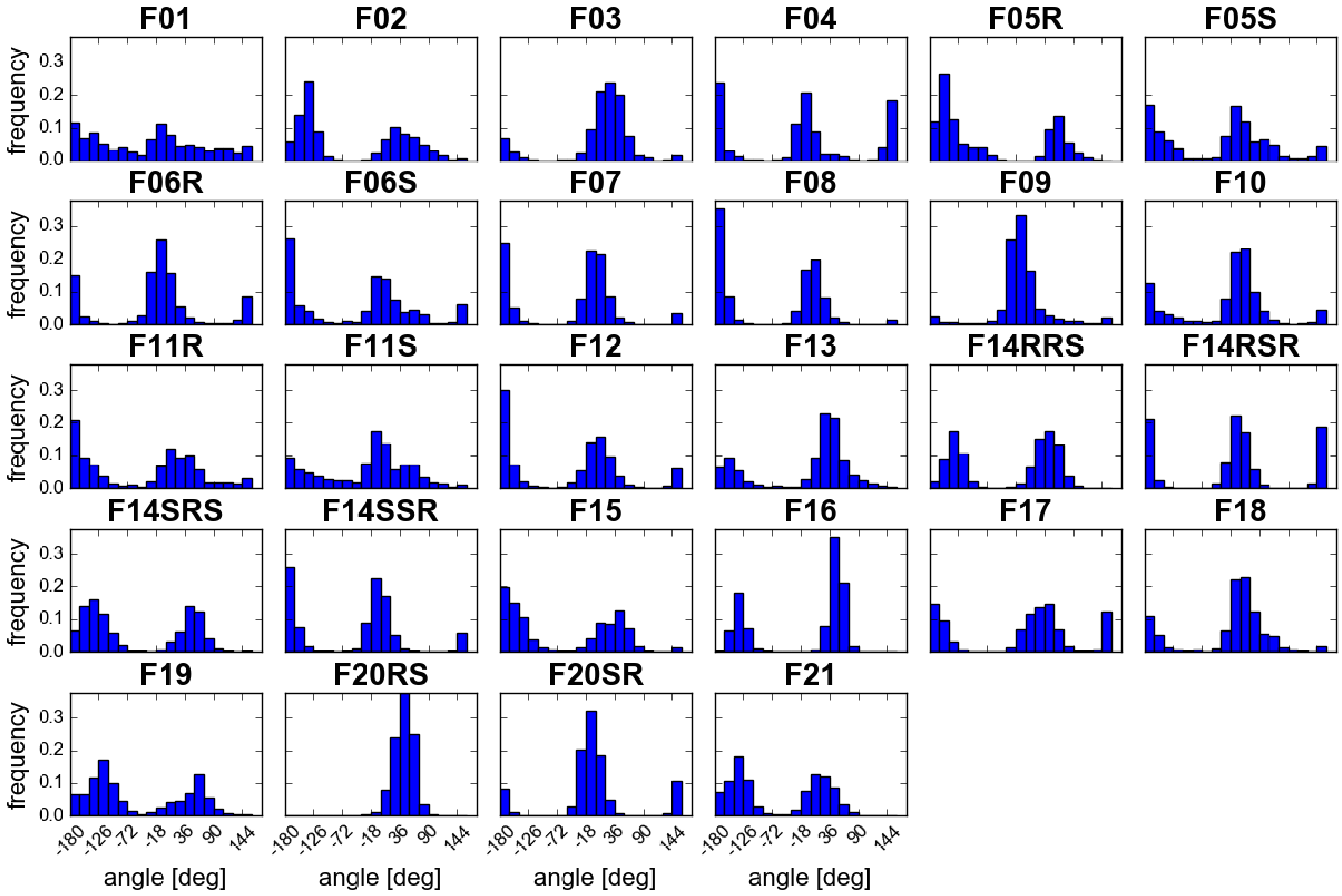


**Figure SM-DIH-3.** Distributions of X2 dihedral angle values of D147.
*Data are collected from last 10ns of production in 3 replicas.*

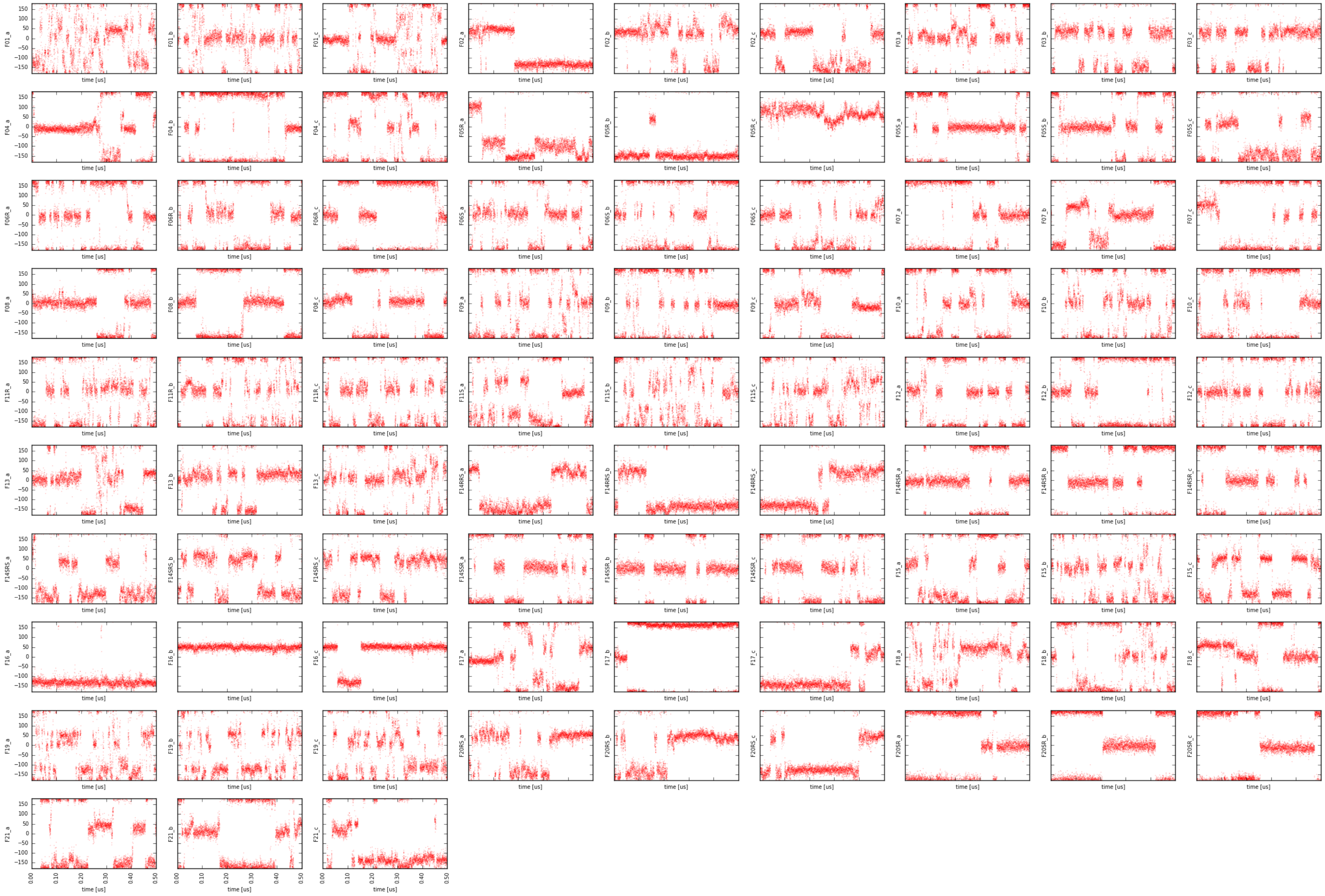

**Figure SM-DIH-4.** Time evolution of X2 dihedral angle of D147.

*Y-values are given in degrees.*

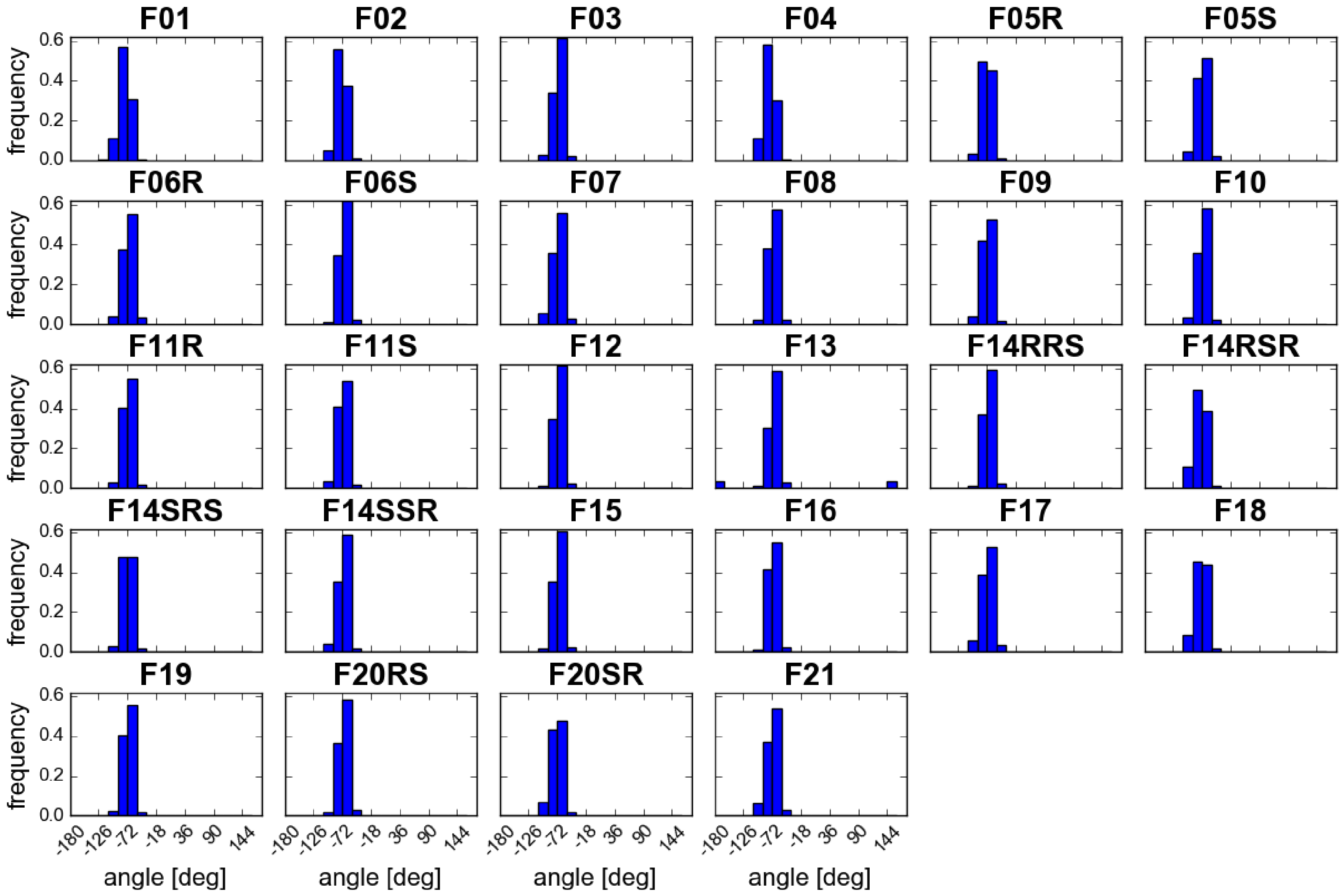


**Figure SM-DIH-5.** Distributions of X1 dihedral angle values of Y148.
*Data are collected from last 10ns of production in 3 replicas.*

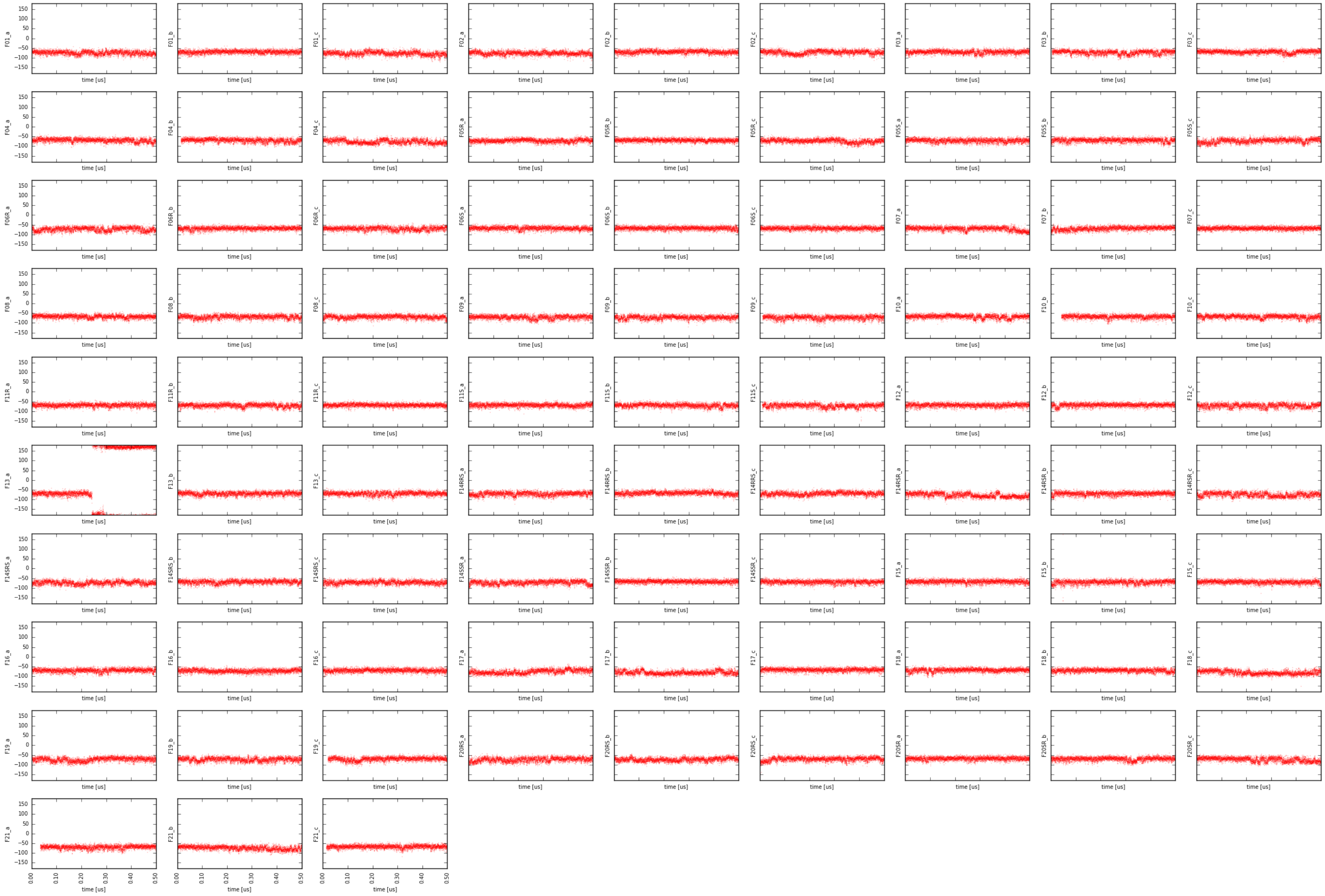

**Figure SM-DIH-6.** Time evolution of X1 dihedral angle of Y148.
*Y-values are given in degrees.*

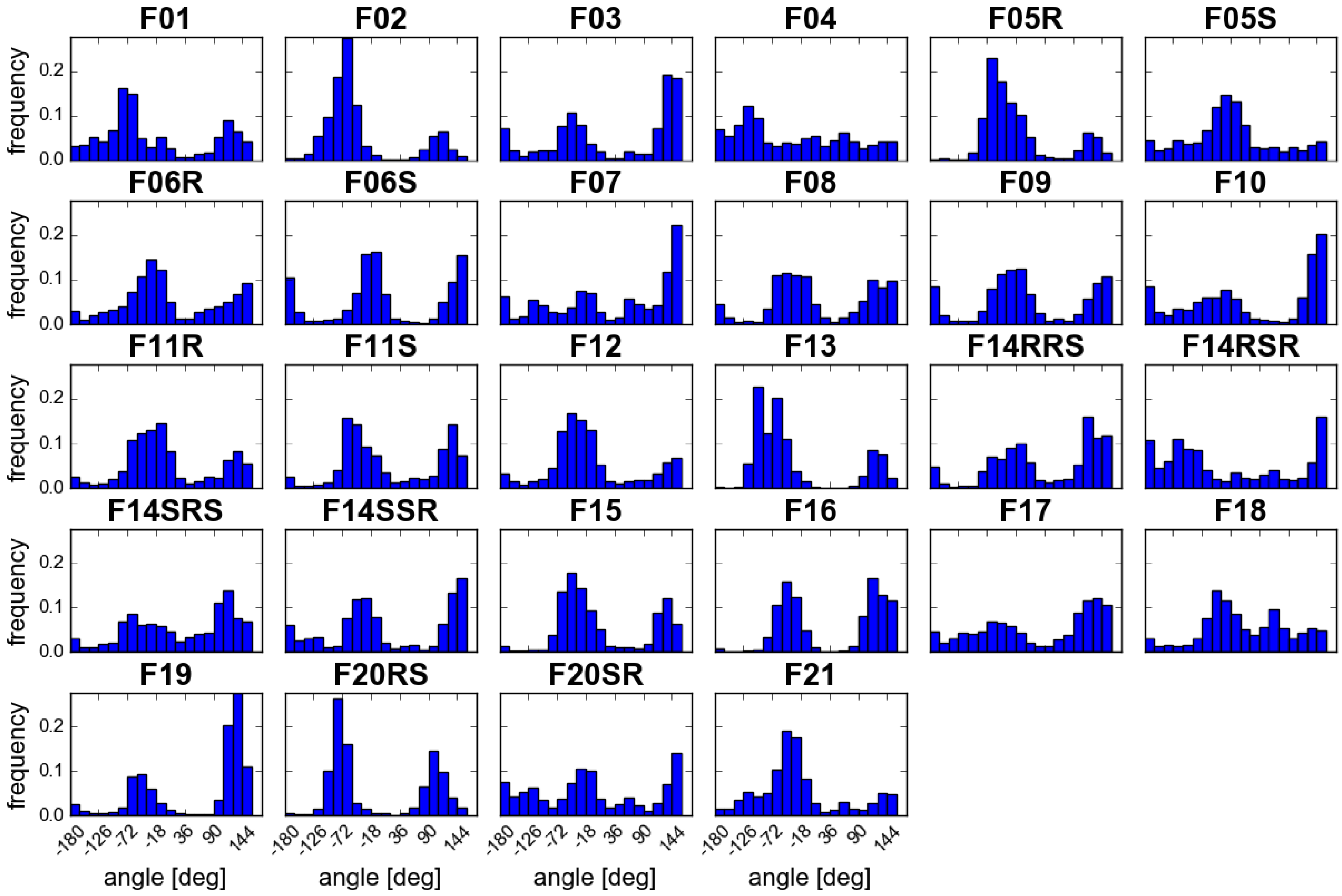


**Figure SM-DIH-7.** Distributions of X2 dihedral angle values of Y148.

*Data are collected from last 10ns of production in 3 replicas.*

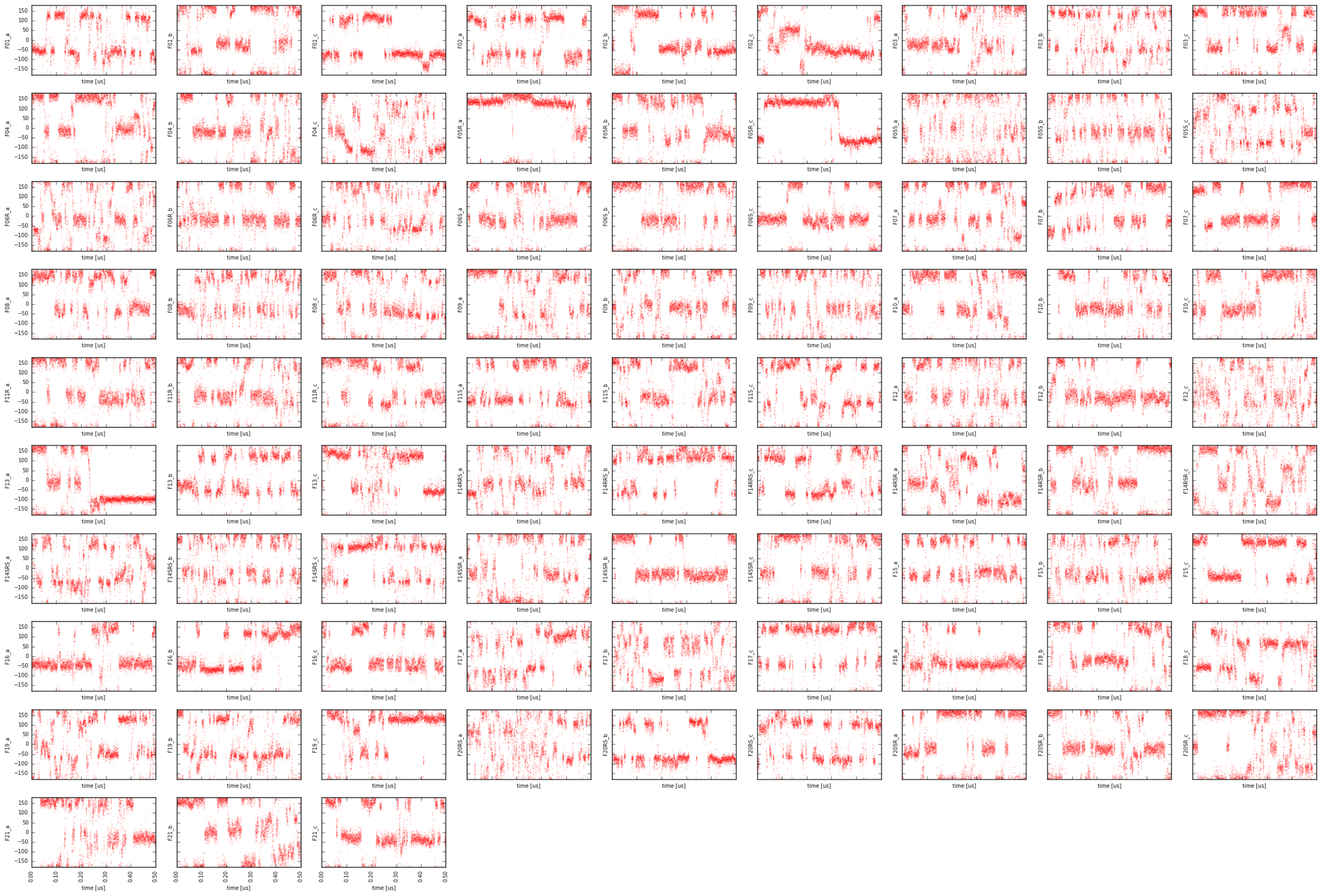


**Figure SM-DIH-8.** Time evolution of X2 dihedral angle of Y148.

*Y-values are given in degrees.*

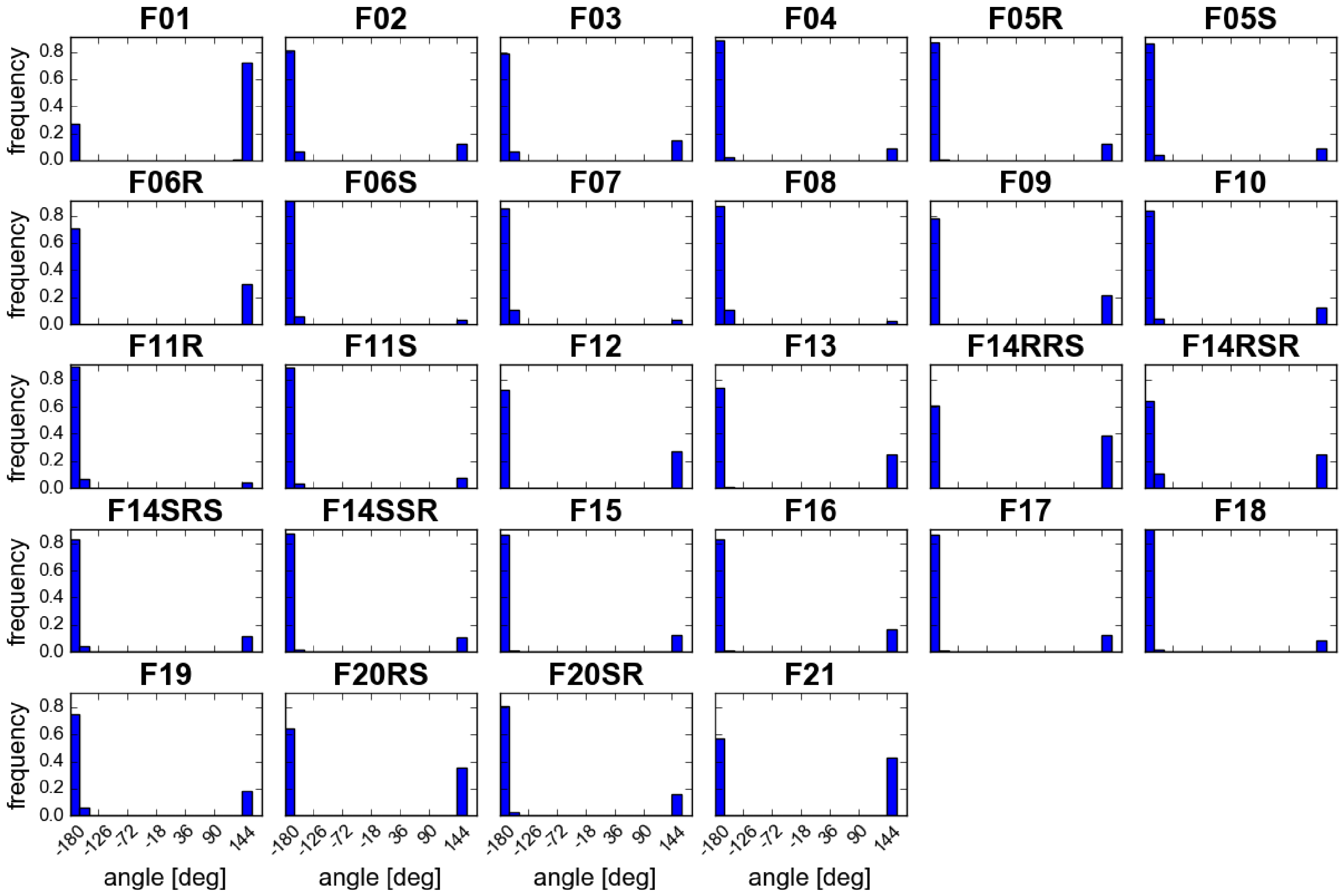


**Figure SM-DIH-9.** Distributions of X1 dihedral angle values of W318.
*Data are collected from last 10ns of production in 3 replicas.*

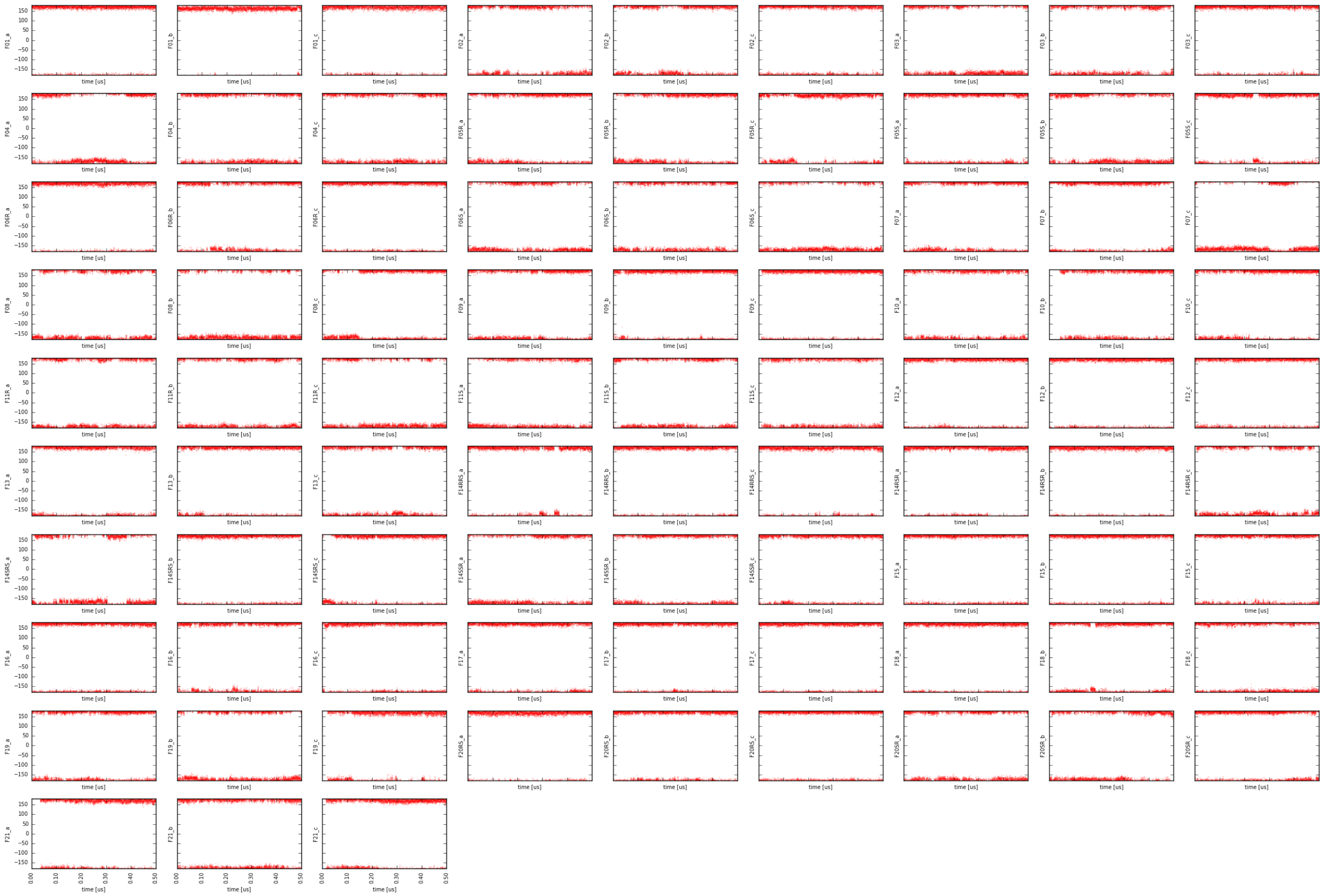

**Figure SM-DIH-10.** Time evolution of X1 dihedral angle of W318.
*Y-values are given in degrees.*

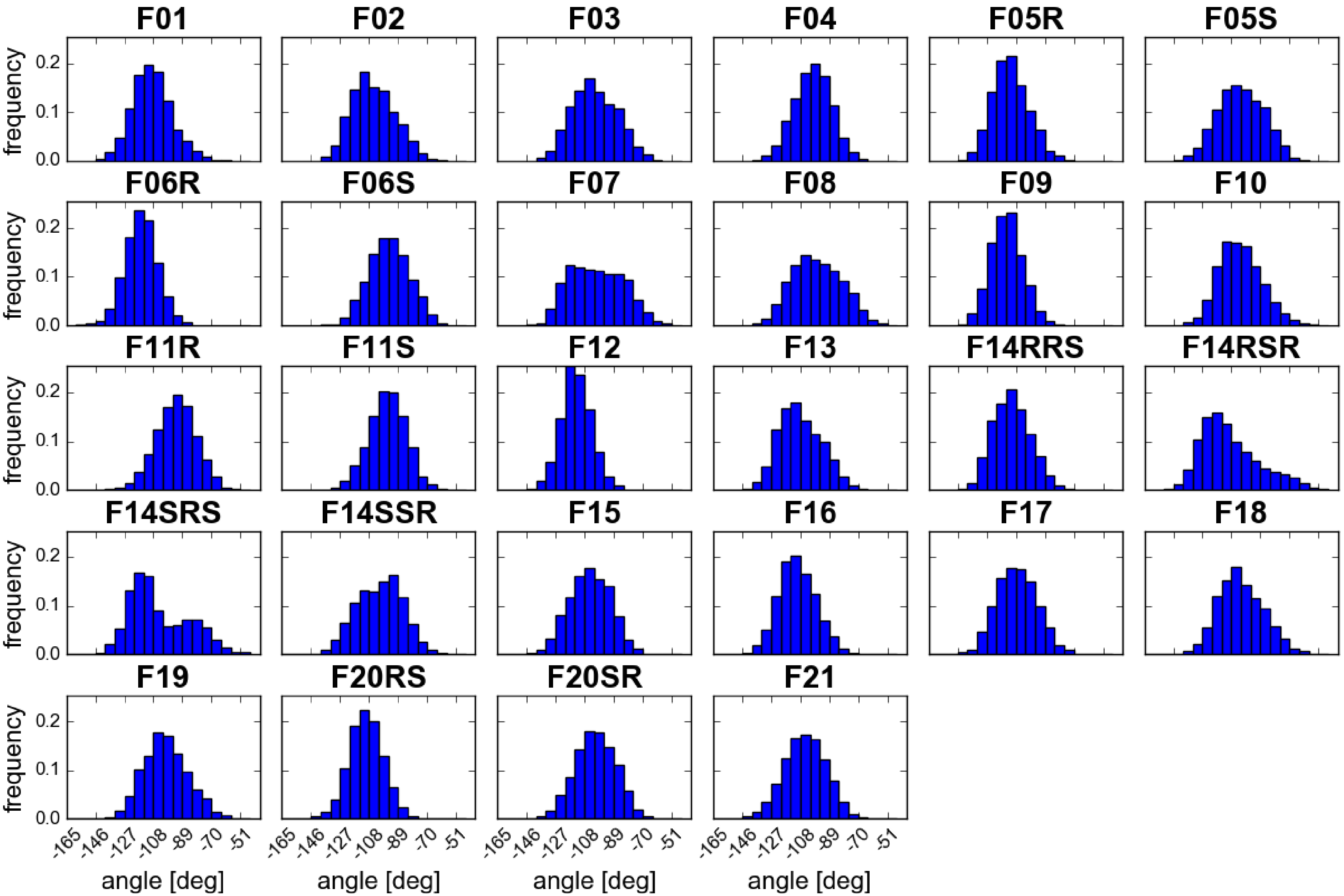


**Figure SM-DIH-11.** Distributions of X2 dihedral angle values of W318.
*Data are collected from last 10ns of production in 3 replicas.*

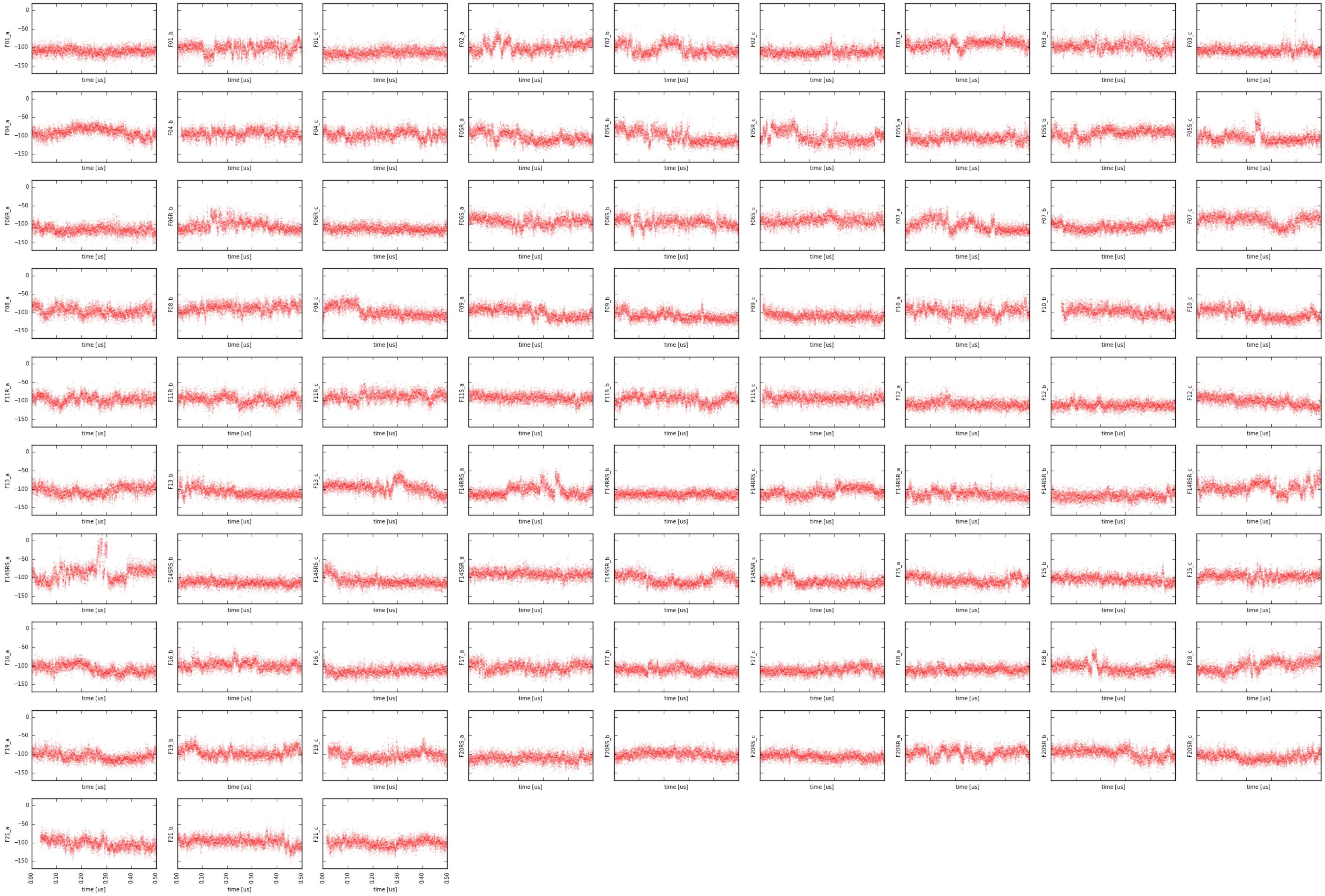


**Figure SM-DIH-12.** Time evolution of X2 dihedral angle of W318.

*Y-values are given in degrees.*

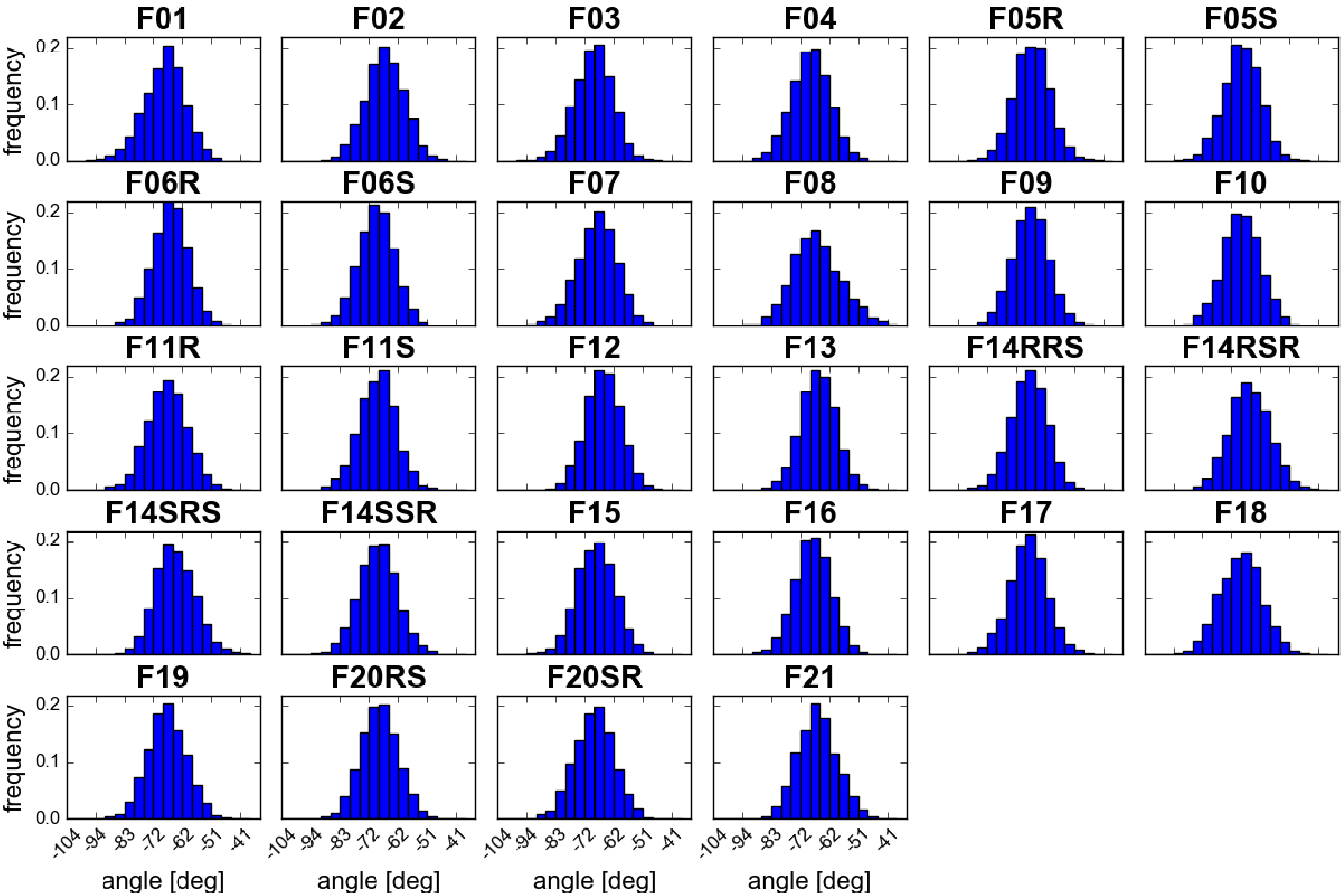


**Figure SM-DIH-13.** Distributions of X1 dihedral angle values of H319.
*Data are collected from last 10ns of production in 3 replicas.*

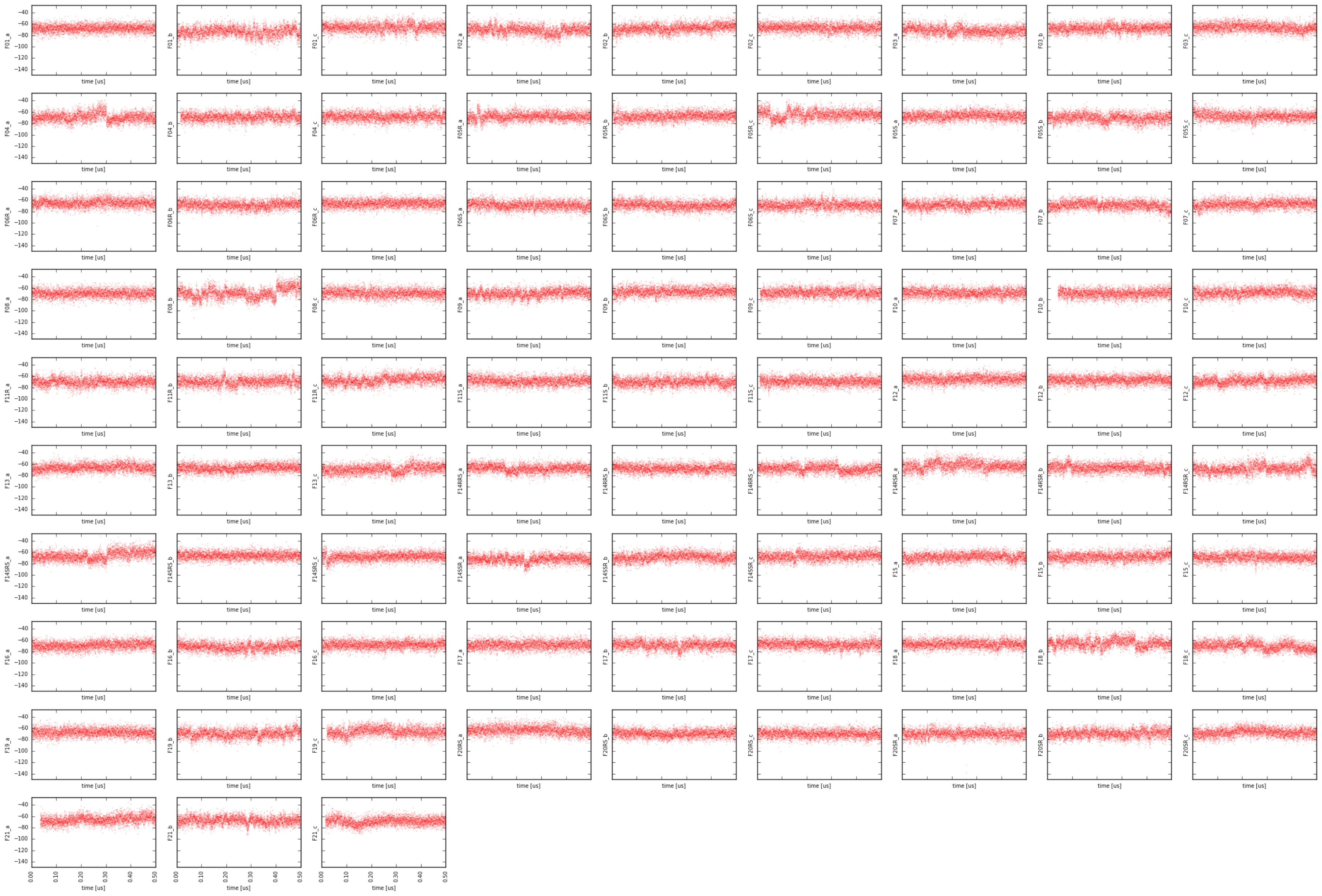


**Figure SM-DIH-14.** Time evolution of X1 dihedral angle of H319.

*Y-values are given in degrees.*

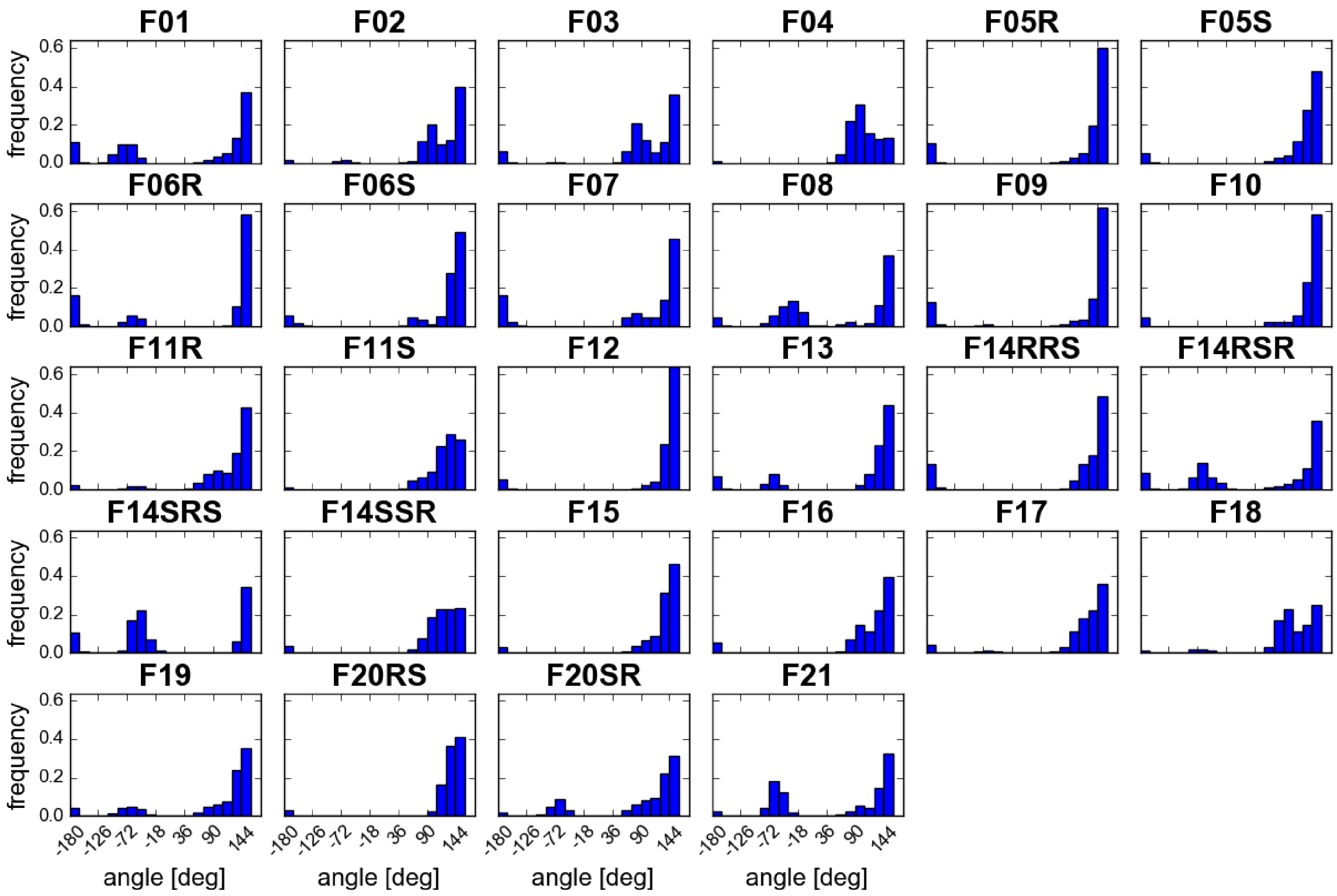


**Figure SM-DIH-15.** Distributions of X2 dihedral angle values of H319.
*Data are collected from last 10ns of production in 3 replicas.*

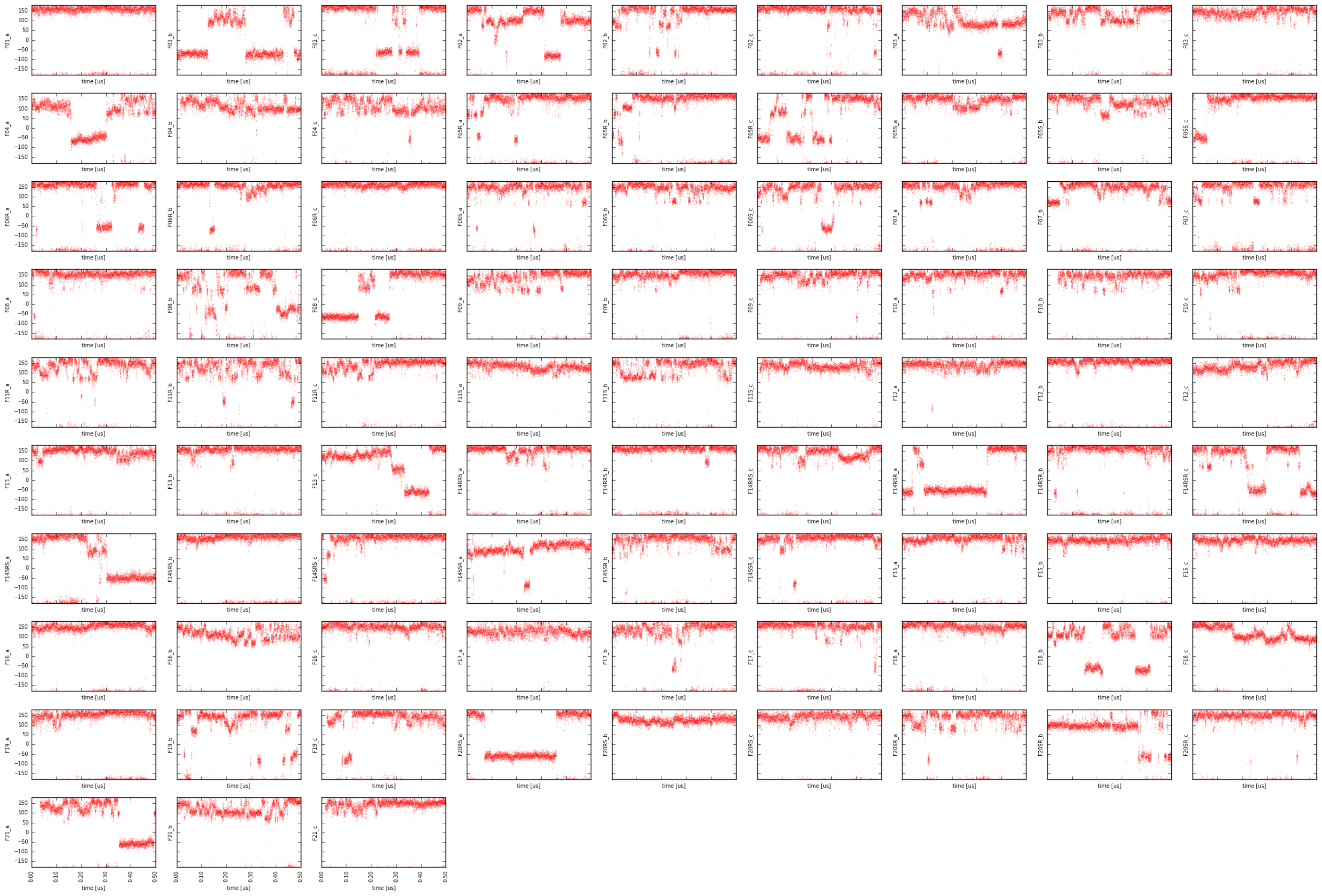

**Figure SM-DIH-16.** Time evolution of X2 dihedral angle of H319.
*Y-values are given in degrees.*

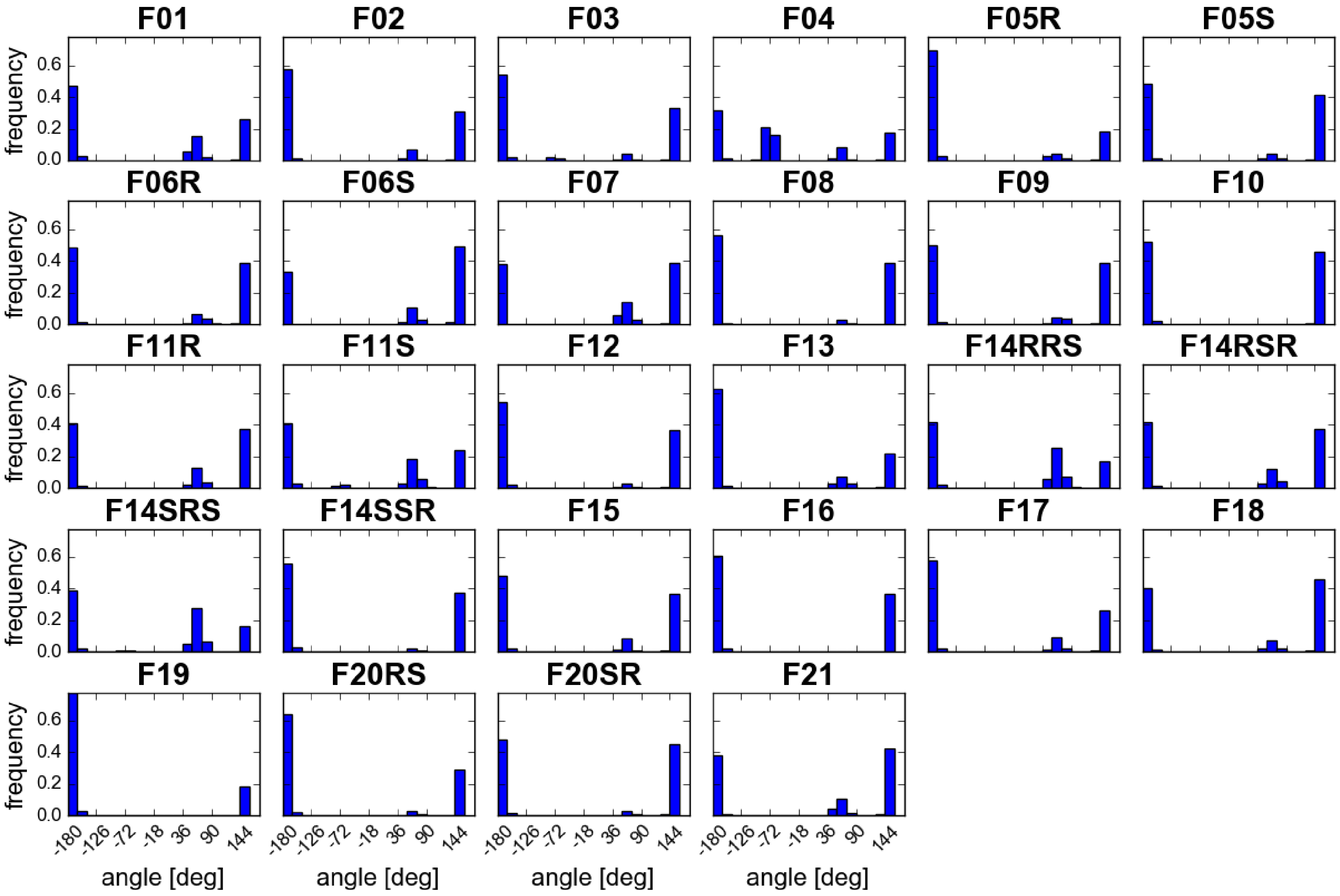


**Figure SM-DIH-17.** Distributions of X1 dihedral angle values of M151.
*Data are collected from last 10ns of production in 3 replicas.*

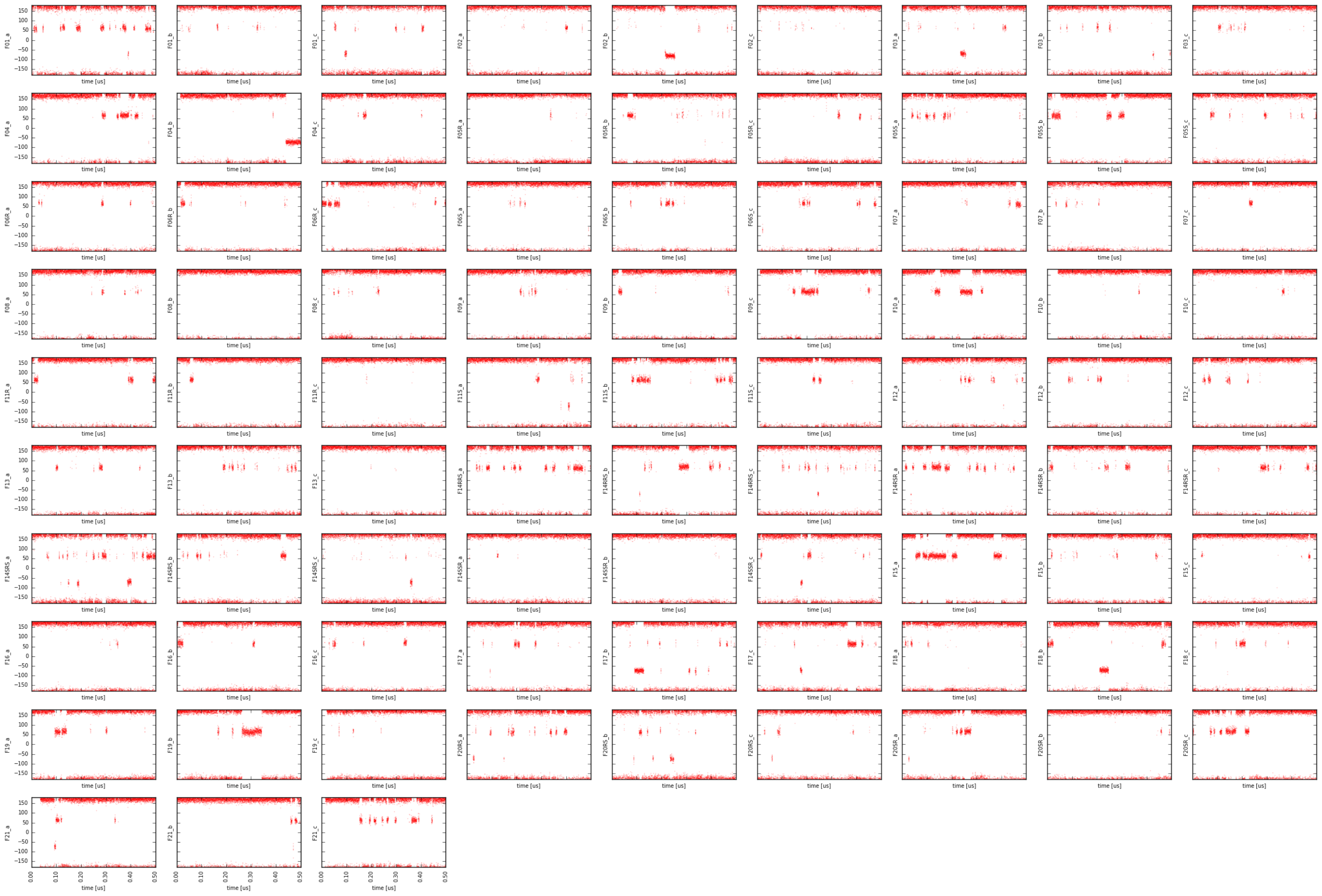


**Figure SM-DIH-18.** Time evolution of X1 dihedral angle of M151.

*Y-values are given in degrees.*

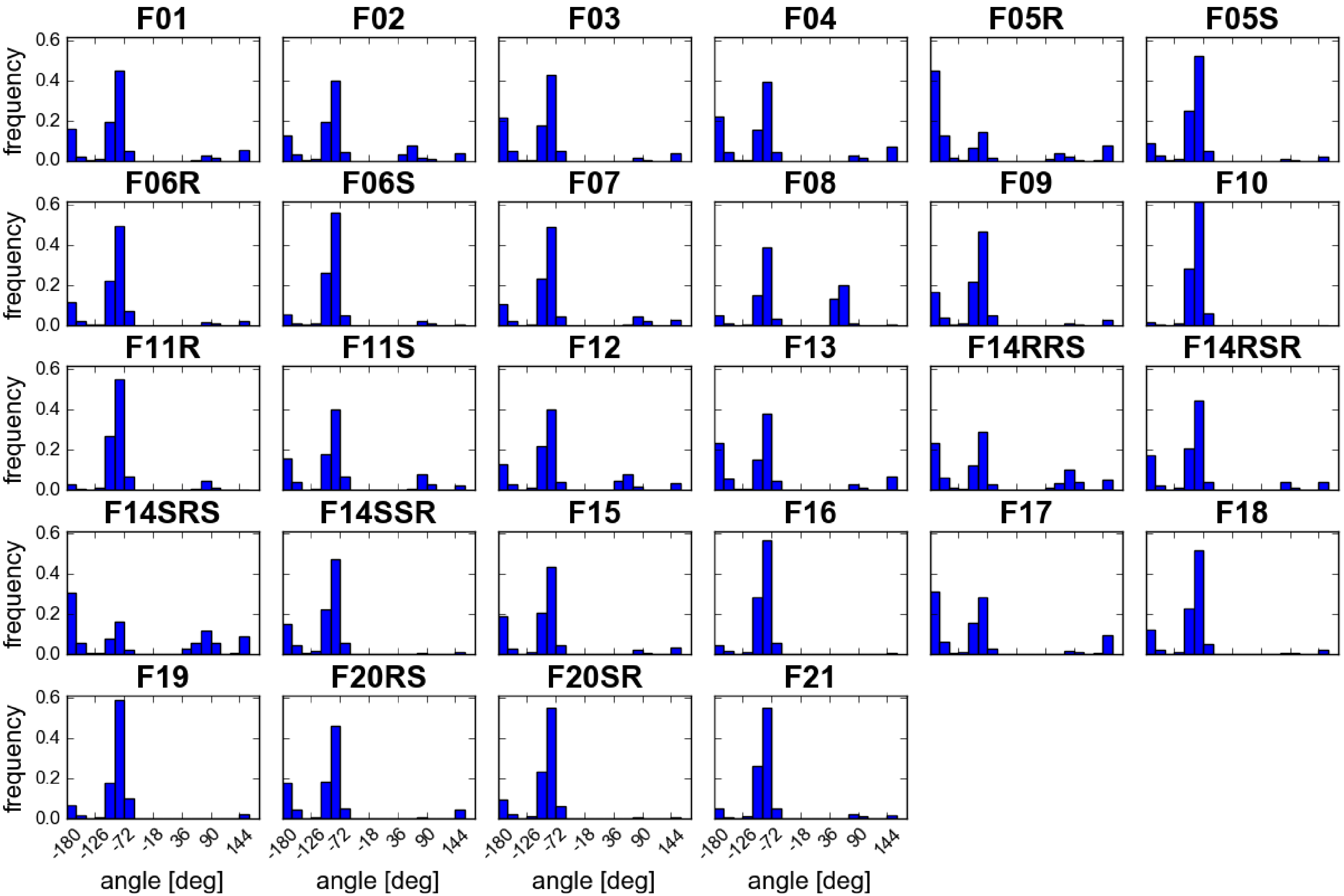


**Figure SM-DIH-19.** Distributions of X2 dihedral angle values of M151.
*Data are collected from last 10ns of production in 3 replicas.*

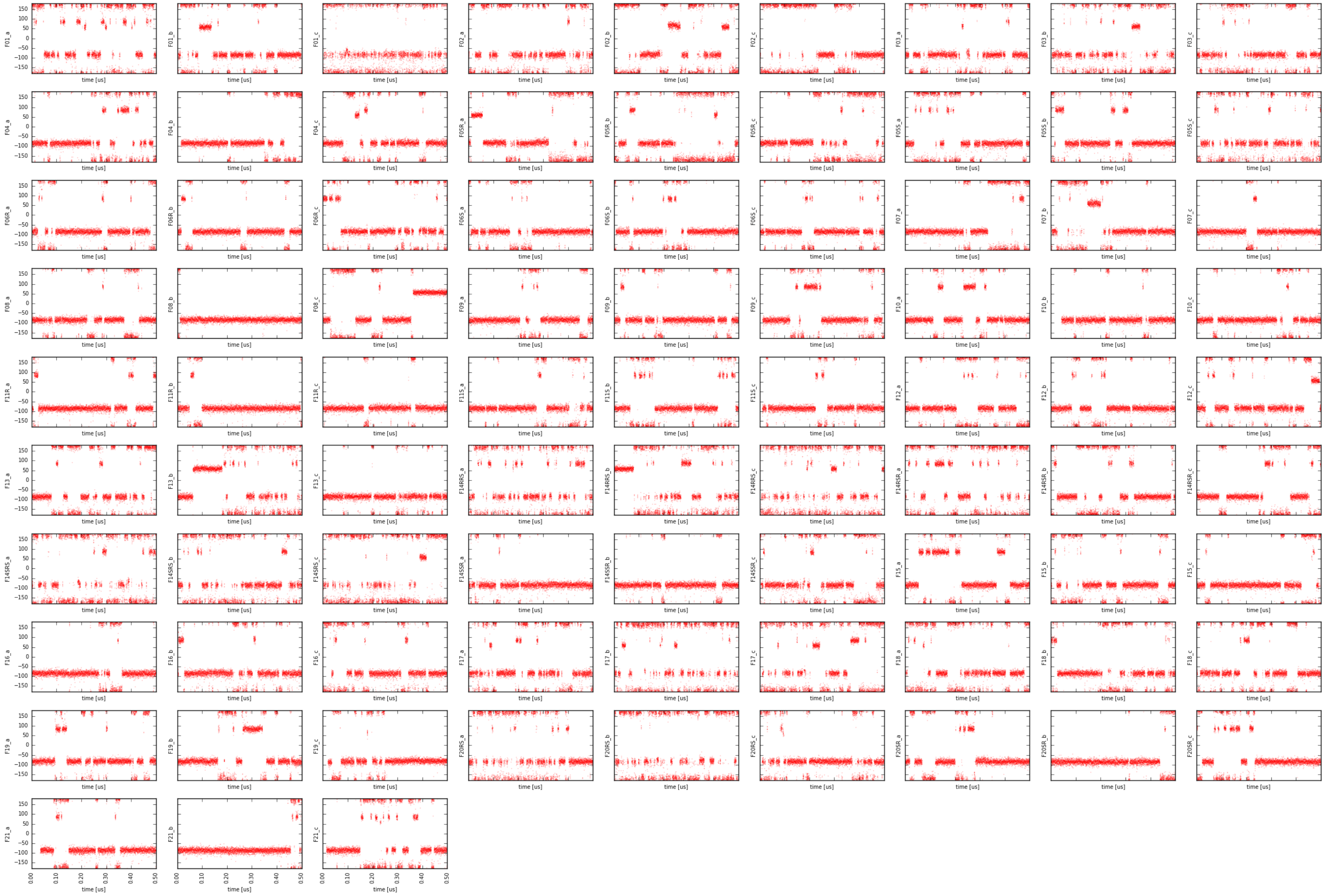


**Figure SM-DIH-20.** Time evolution of X2 dihedral angle of M151.

*Y-values are given in degrees.*

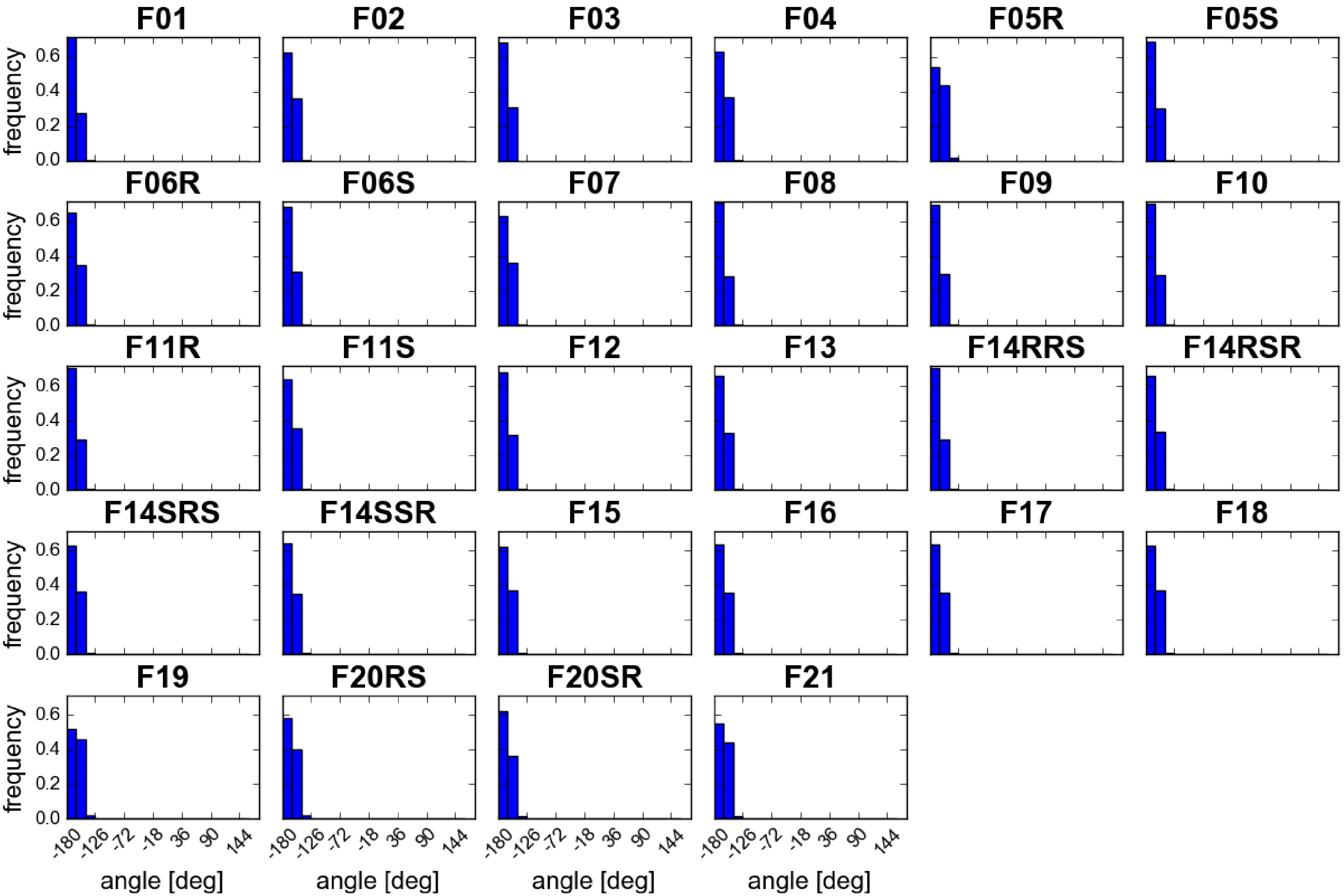


**Figure SM-DIH-21.** Distributions of X1 dihedral angle values of W293.
*Data are collected from last 10ns of production in 3 replicas.*

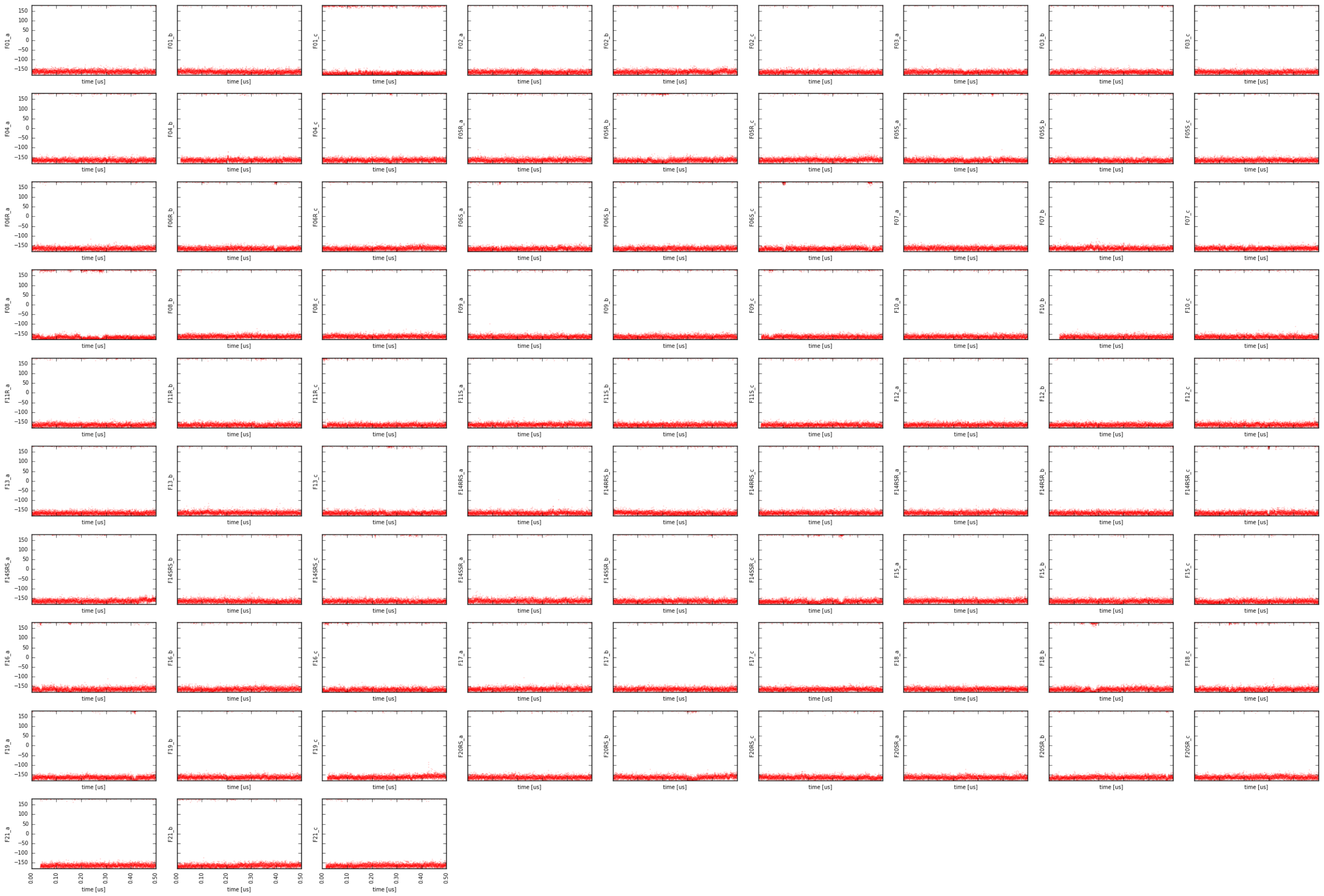

**Figure SM-DIH-22.** Time evolution of X1 dihedral angle of W293.
*Y-values are given in degrees.*

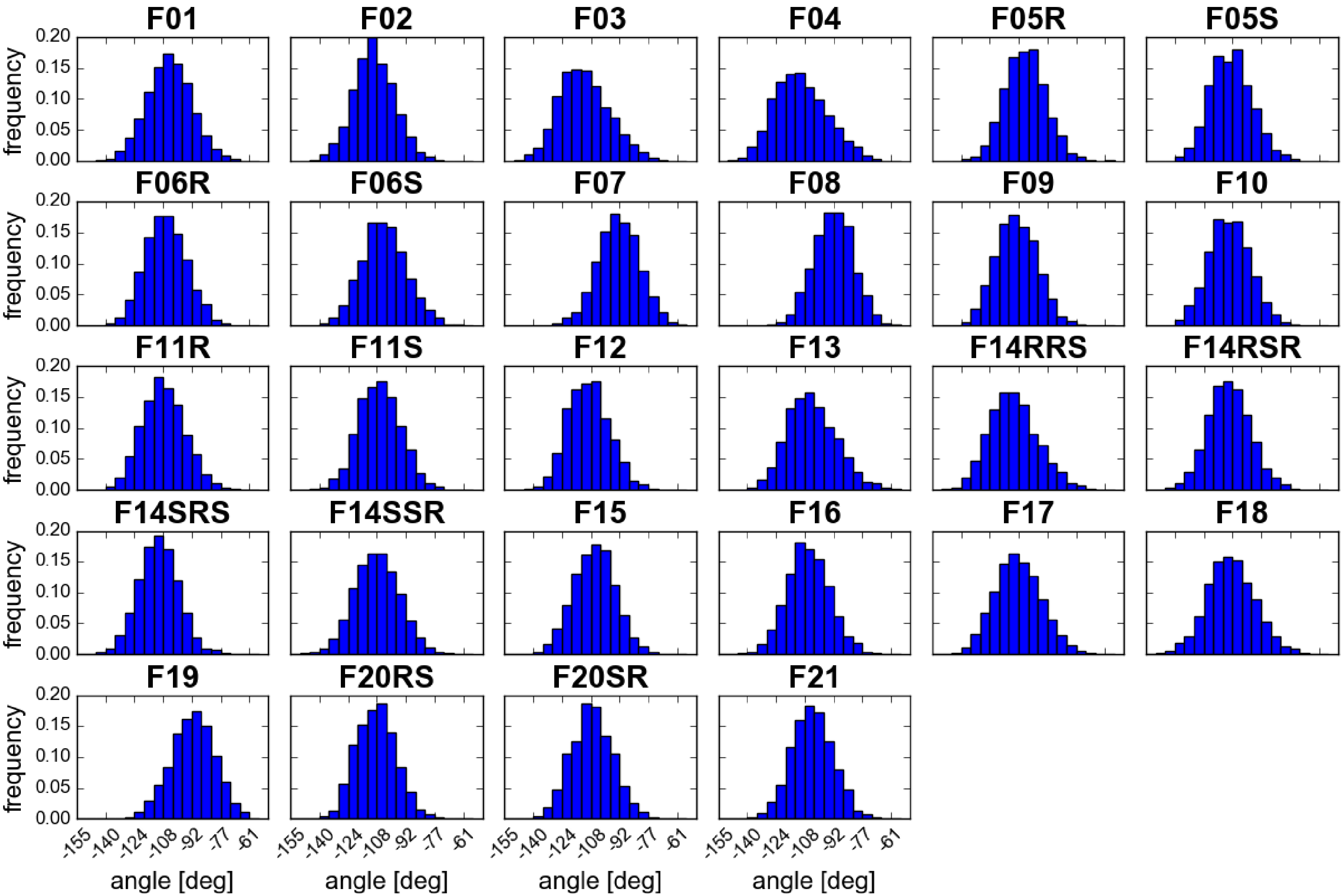


**Figure SM-DIH-23.** Distributions of X2 dihedral angle values of W293.
*Data are collected from last 10ns of production in 3 replicas.*

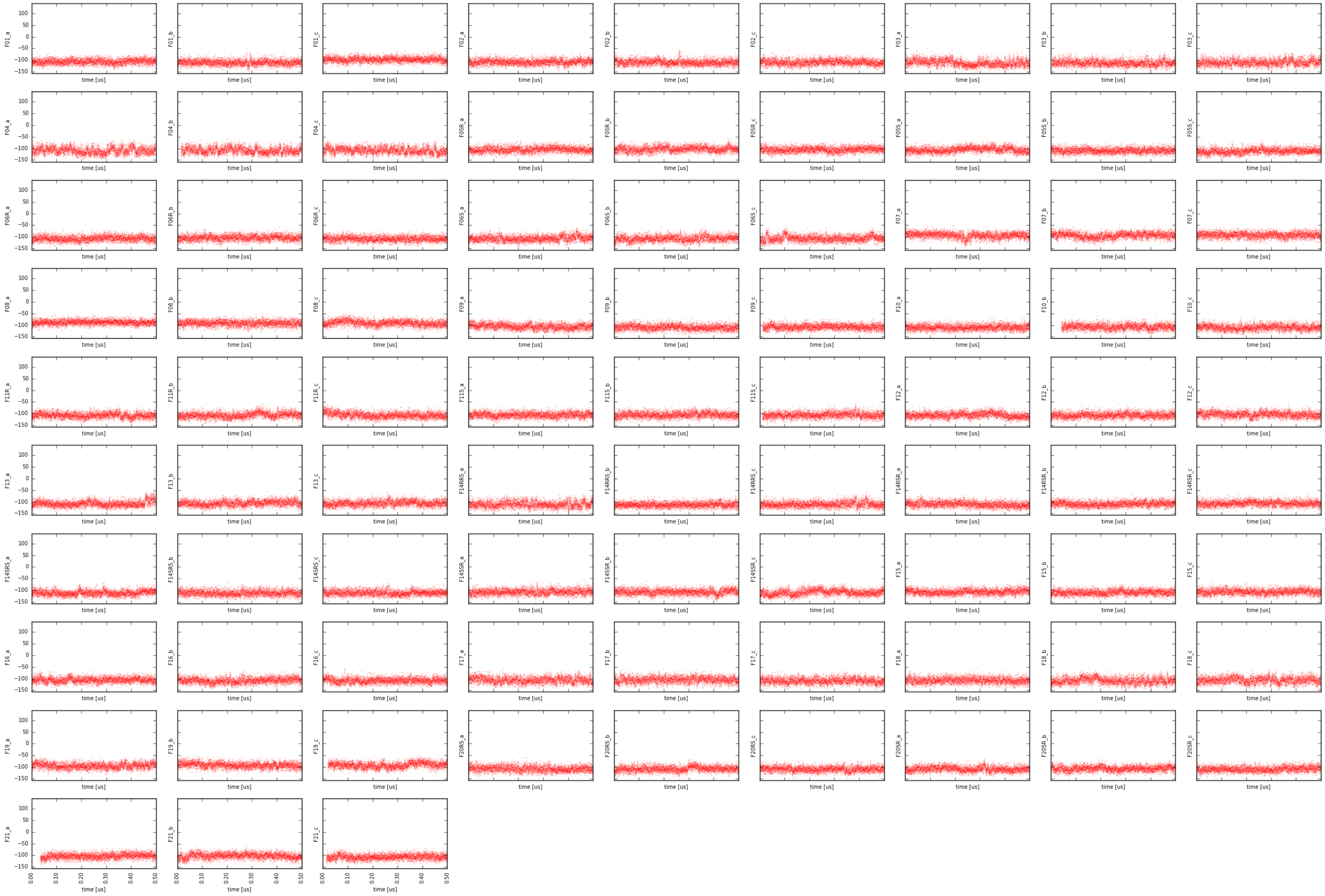


**Figure SM-DIH-24.** Time evolution of X2 dihedral angle of W293.
*Y-values are given in degrees.*

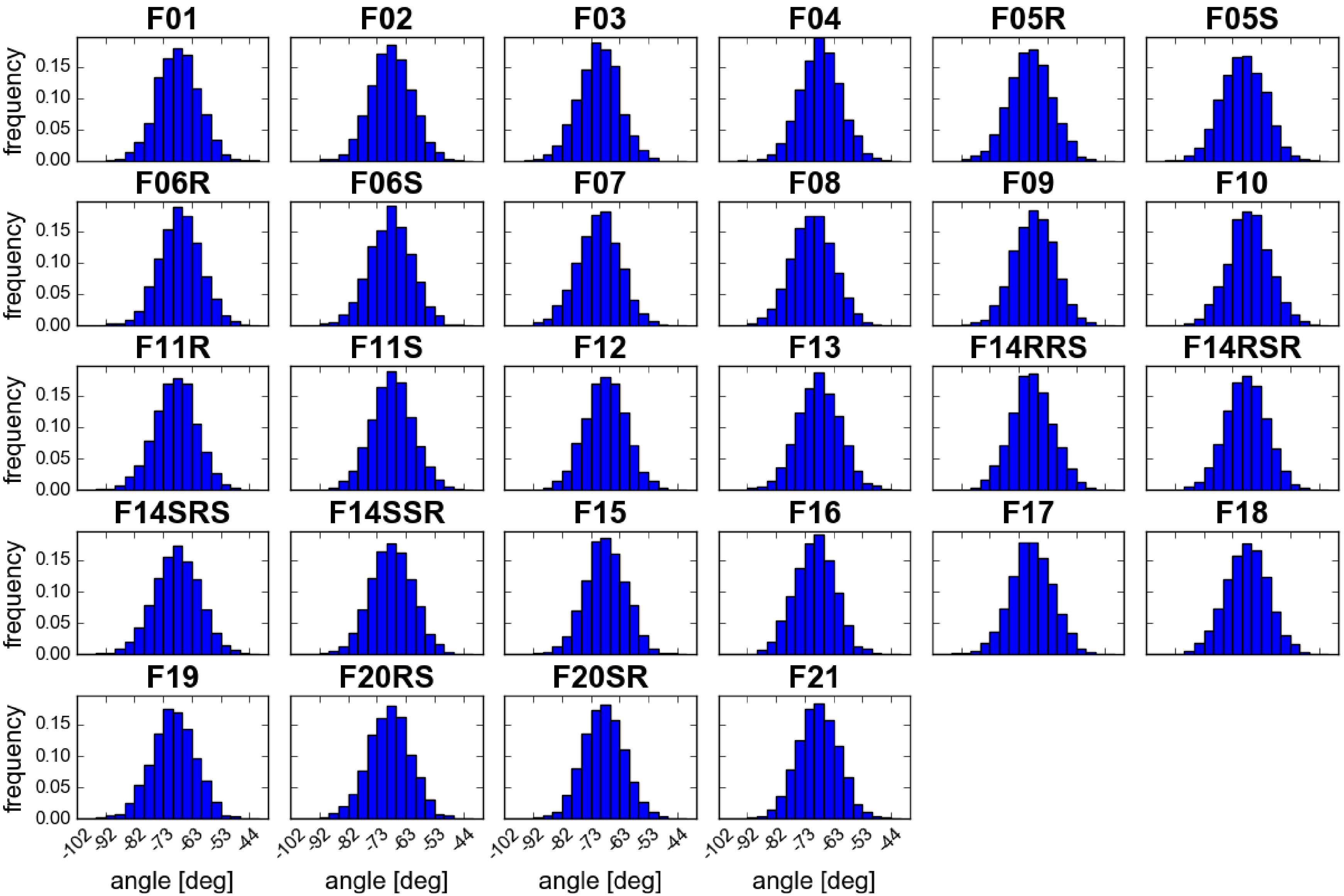


**Figure SM-DIH-25.** Distributions of X1 dihedral angle values of H297.
*Data are collected from last 10ns of production in 3 replicas.*

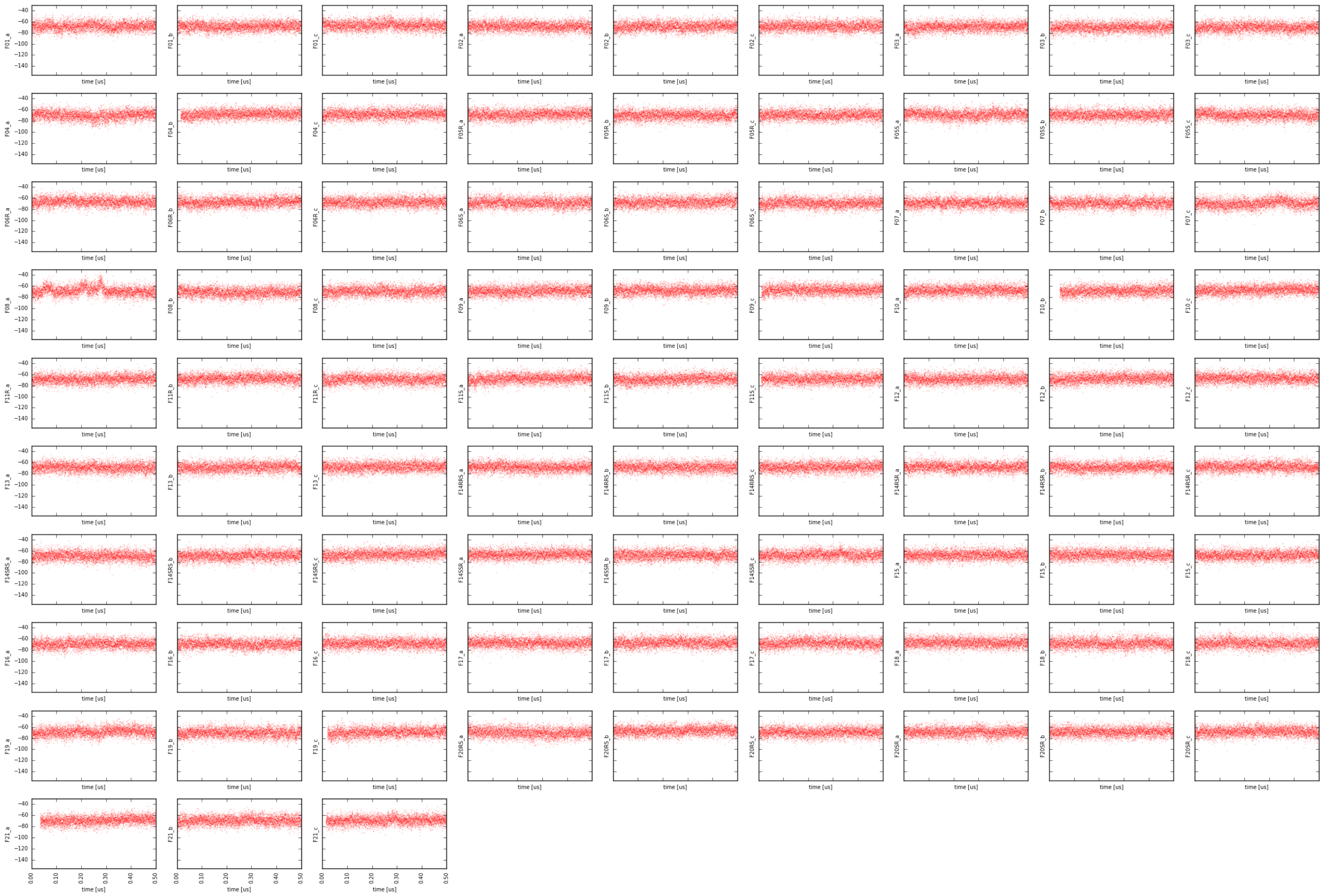

**Figure SM-DIH-26.** Time evolution of X1 dihedral angle of H297.
*Y-values are given in degrees.*

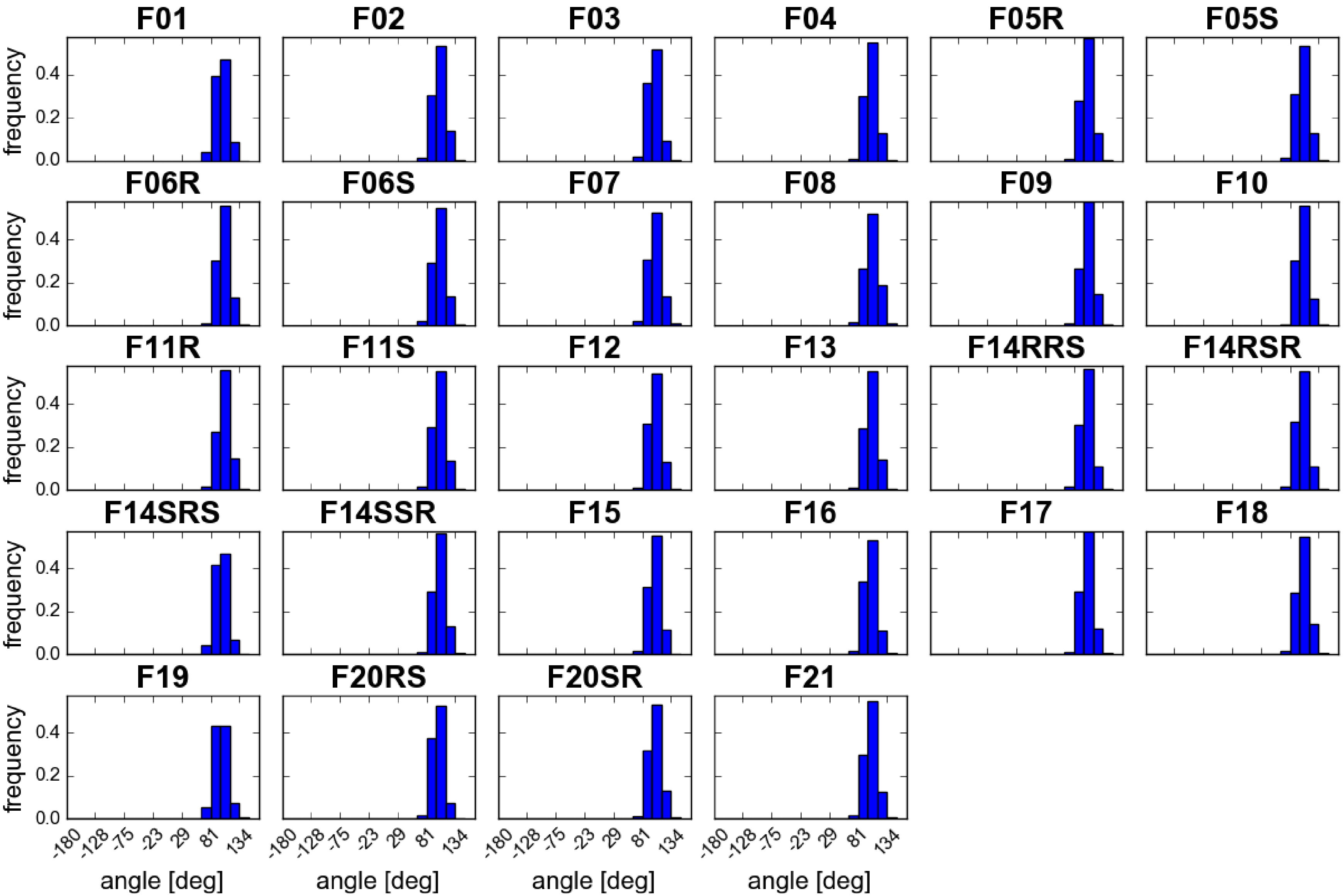


**Figure SM-DIH-27.** Distributions of X2 dihedral angle values of H297.
*Data are collected from last 10ns of production in 3 replicas.*

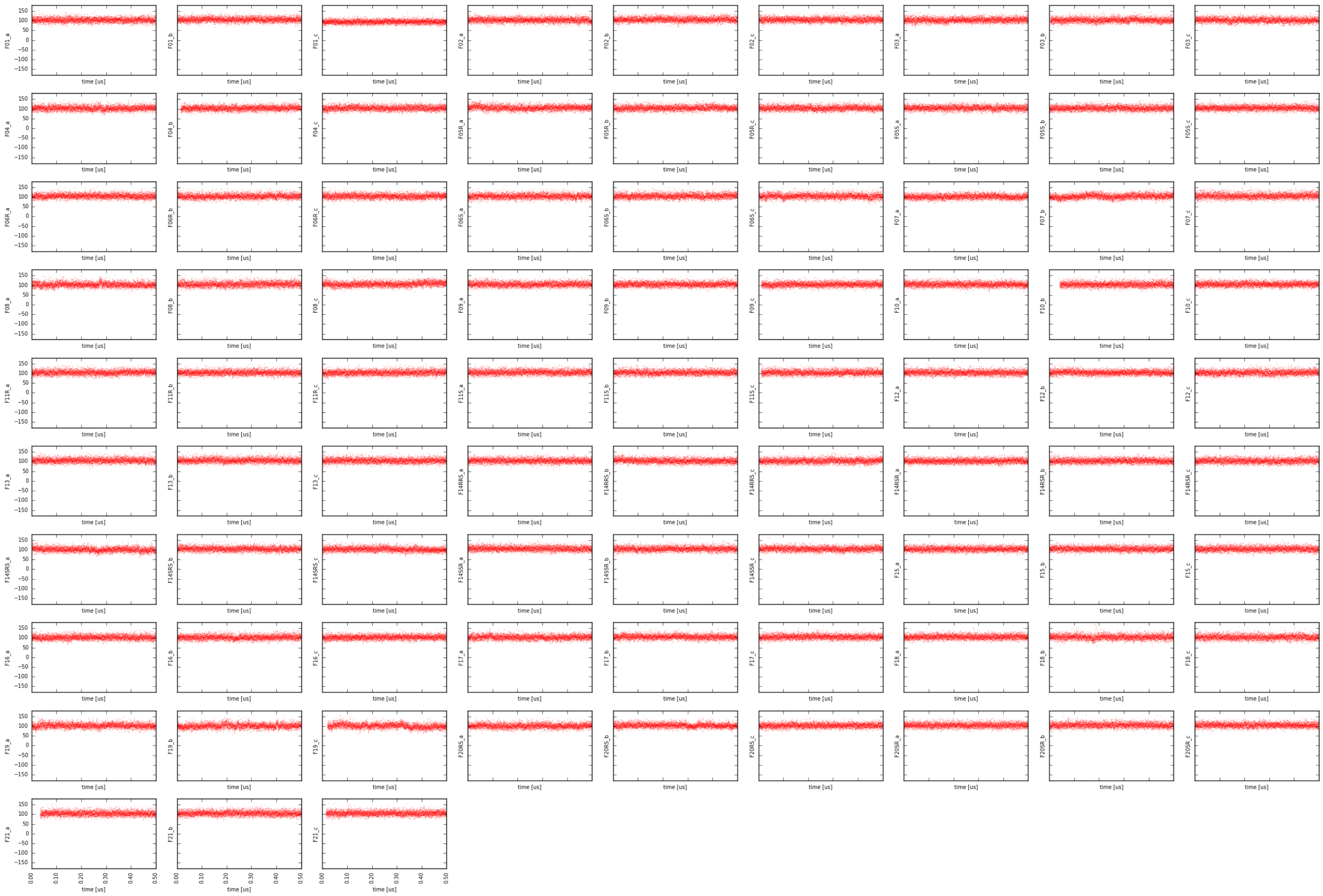

**Figure SM-DIH-28.** Time evolution of X2 dihedral angle of H297.

*Y-values are given in degrees.*

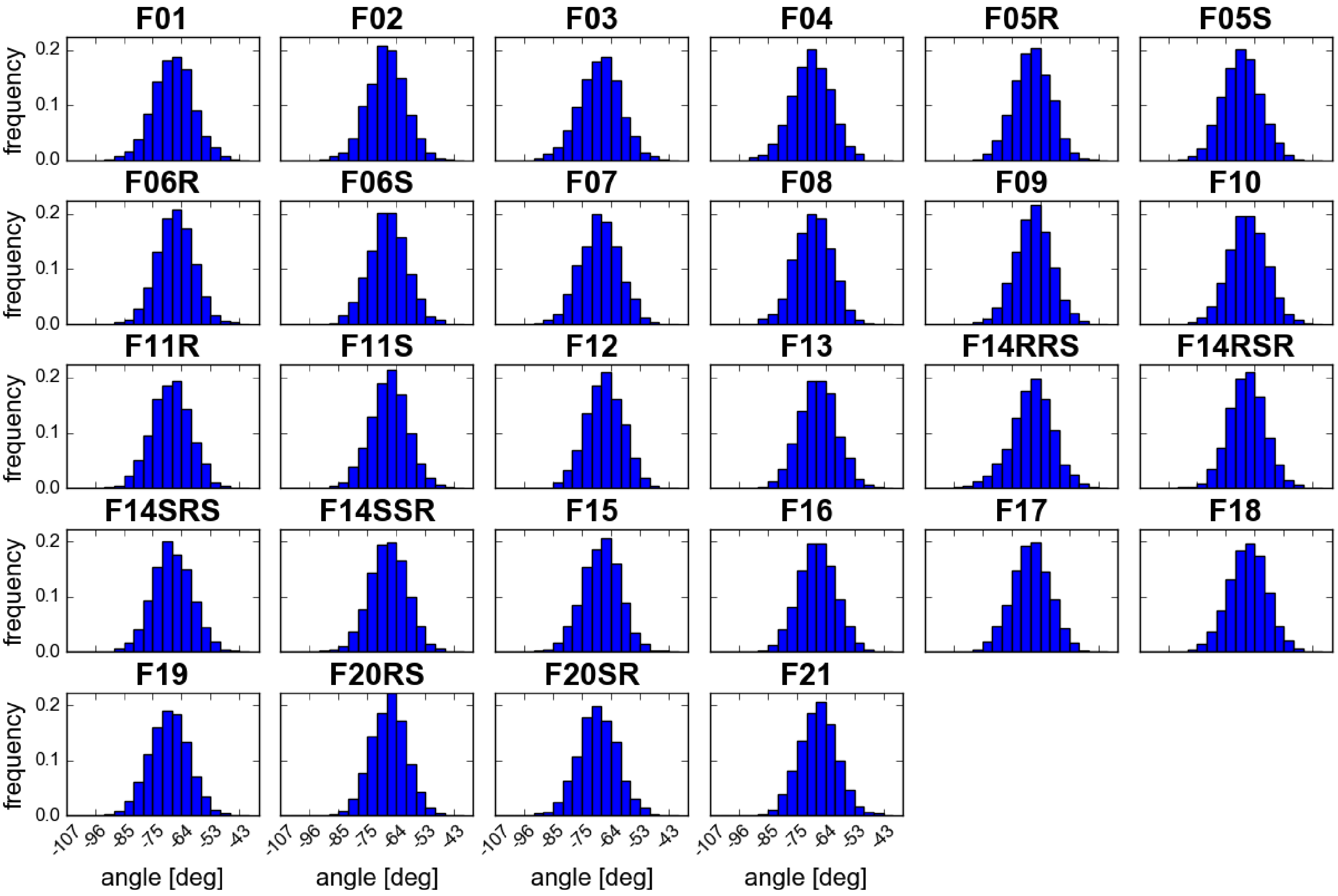


**Figure SM-DIH-29.** Distributions of X1 dihedral angle values of Y326.
*Data are collected from last 10ns of production in 3 replicas.*

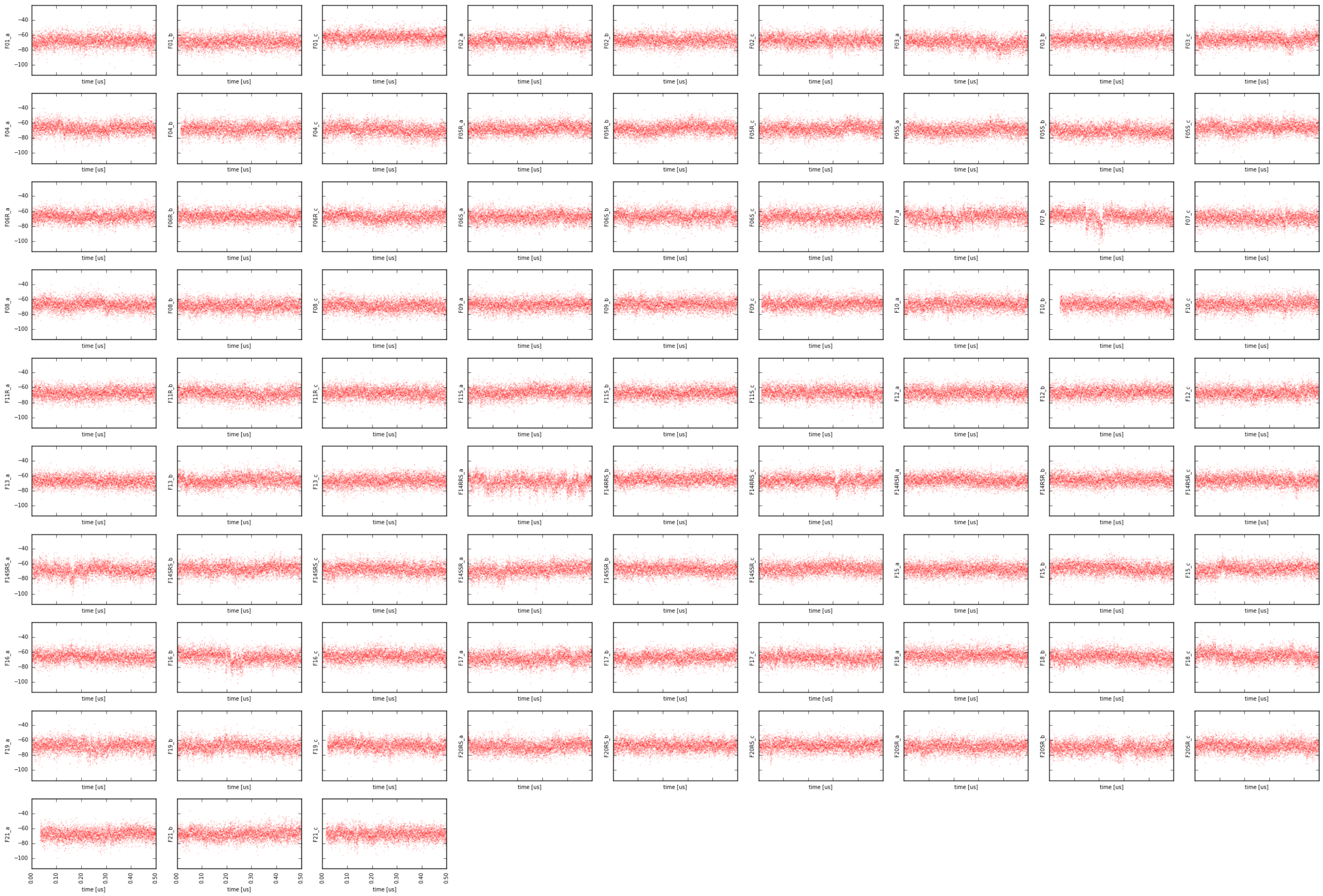

**Figure SM-DIH-30.** Time evolution of X1 dihedral angle of Y326.
*Y-values are given in degrees.*

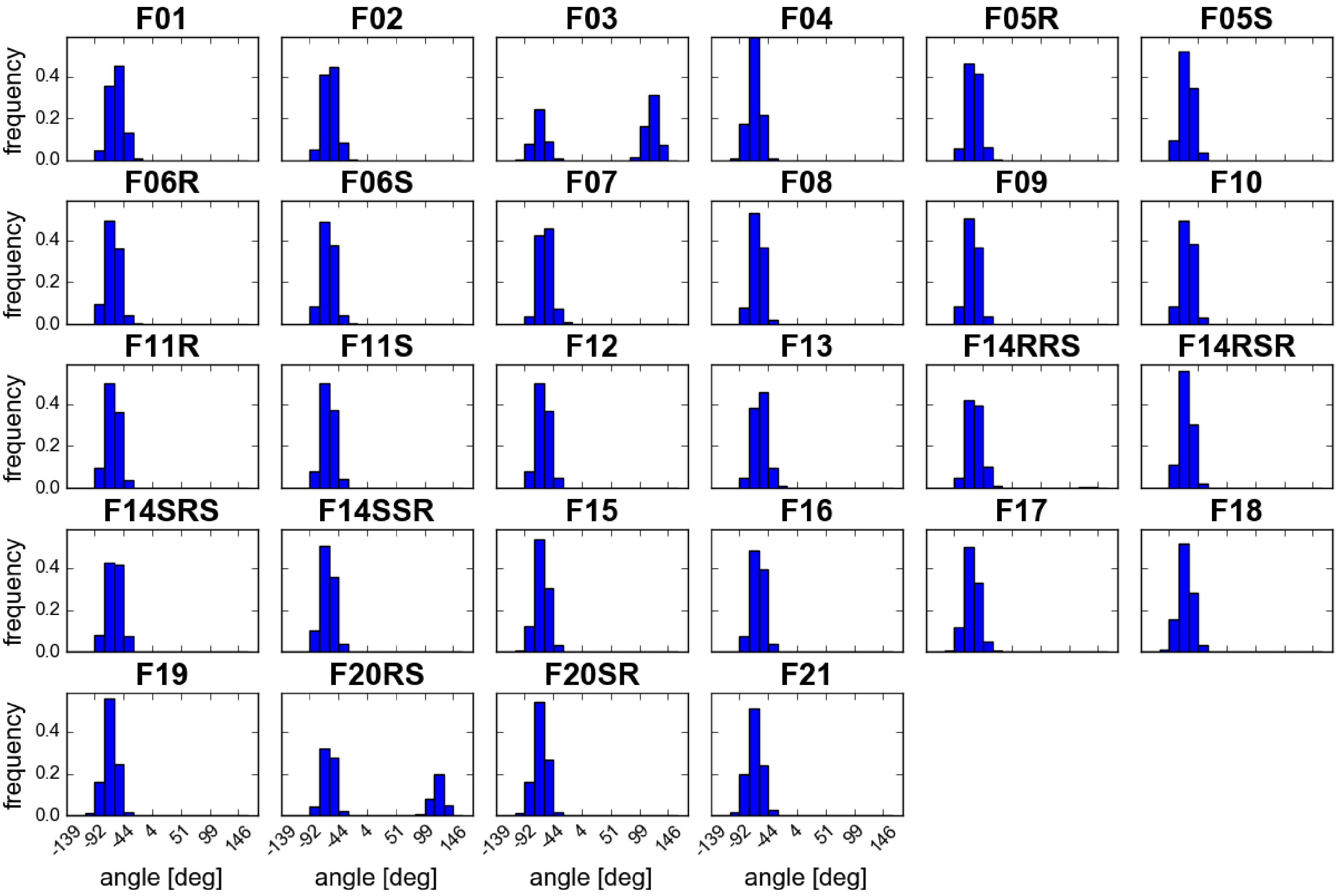


**Figure SM-DIH-31.** Distributions of X2 dihedral angle values of Y326.
*Data are collected from last 10ns of production in 3 replicas.*

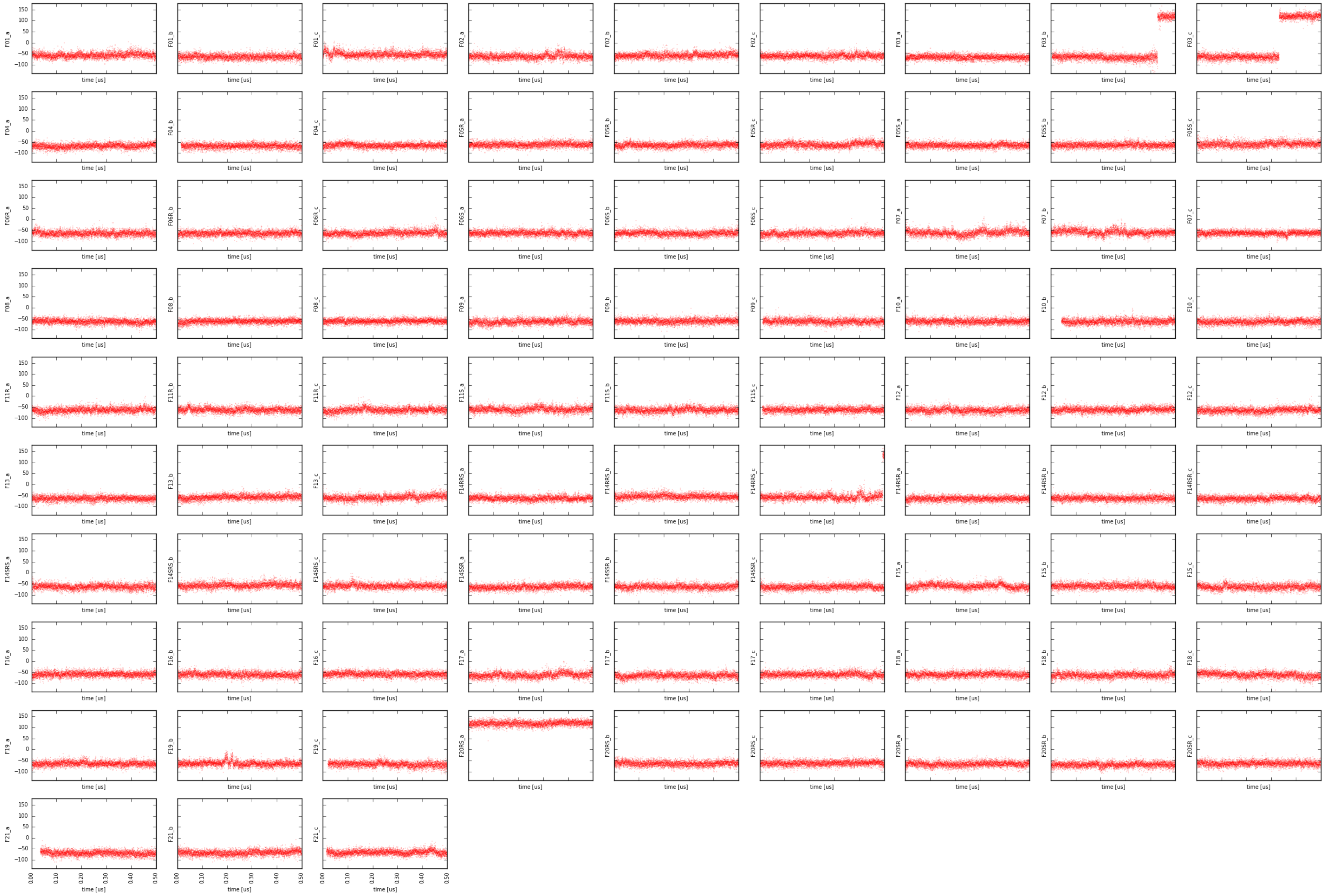


**Figure SM-DIH-32.** Time evolution of X2 dihedral angle of Y326.

*Y-values are given in degrees.*

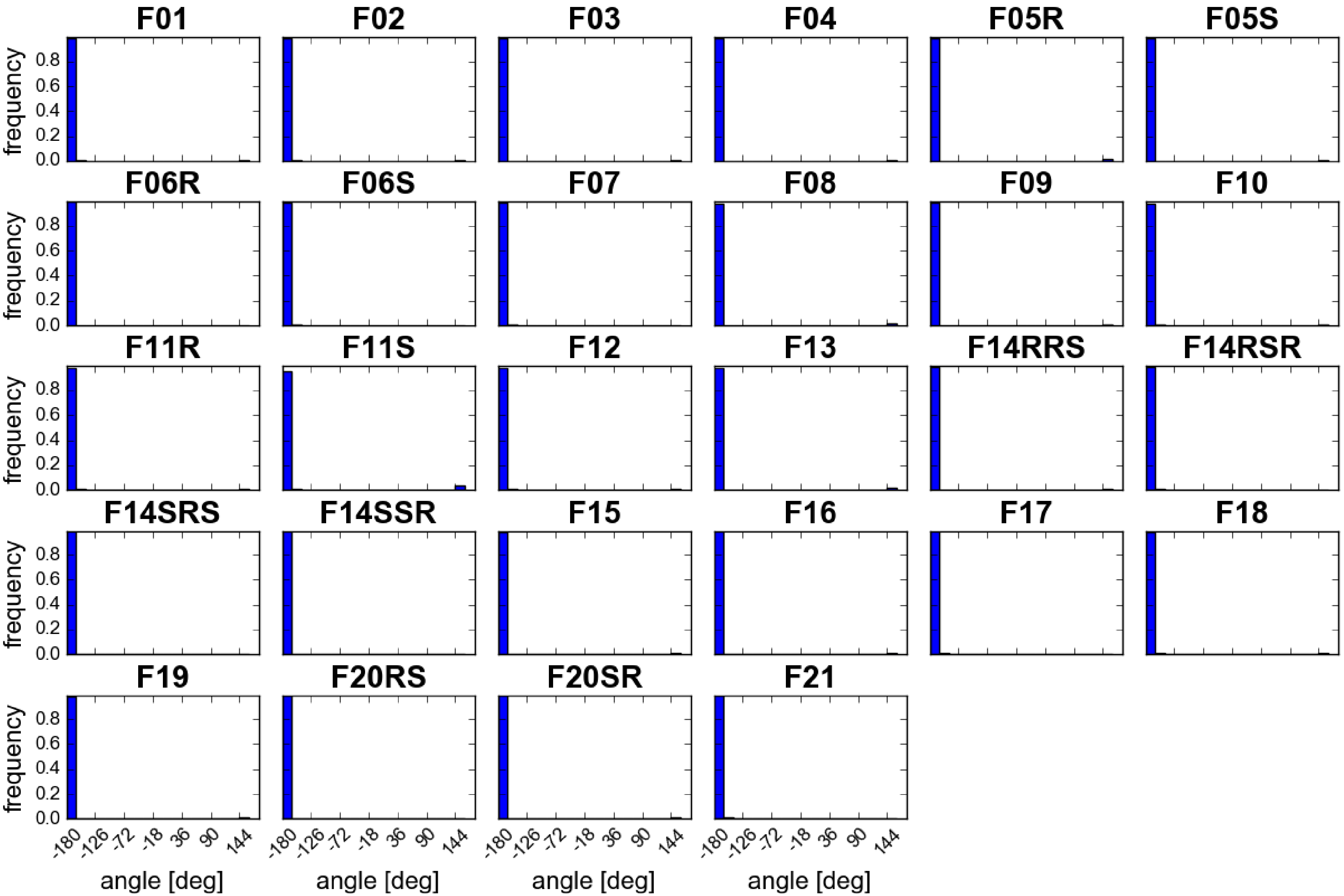


**Figure SM-DIH-33.** Distributions of X1 dihedral angle values of W133.

*Data are collected from last 10ns of production in 3 replicas.*

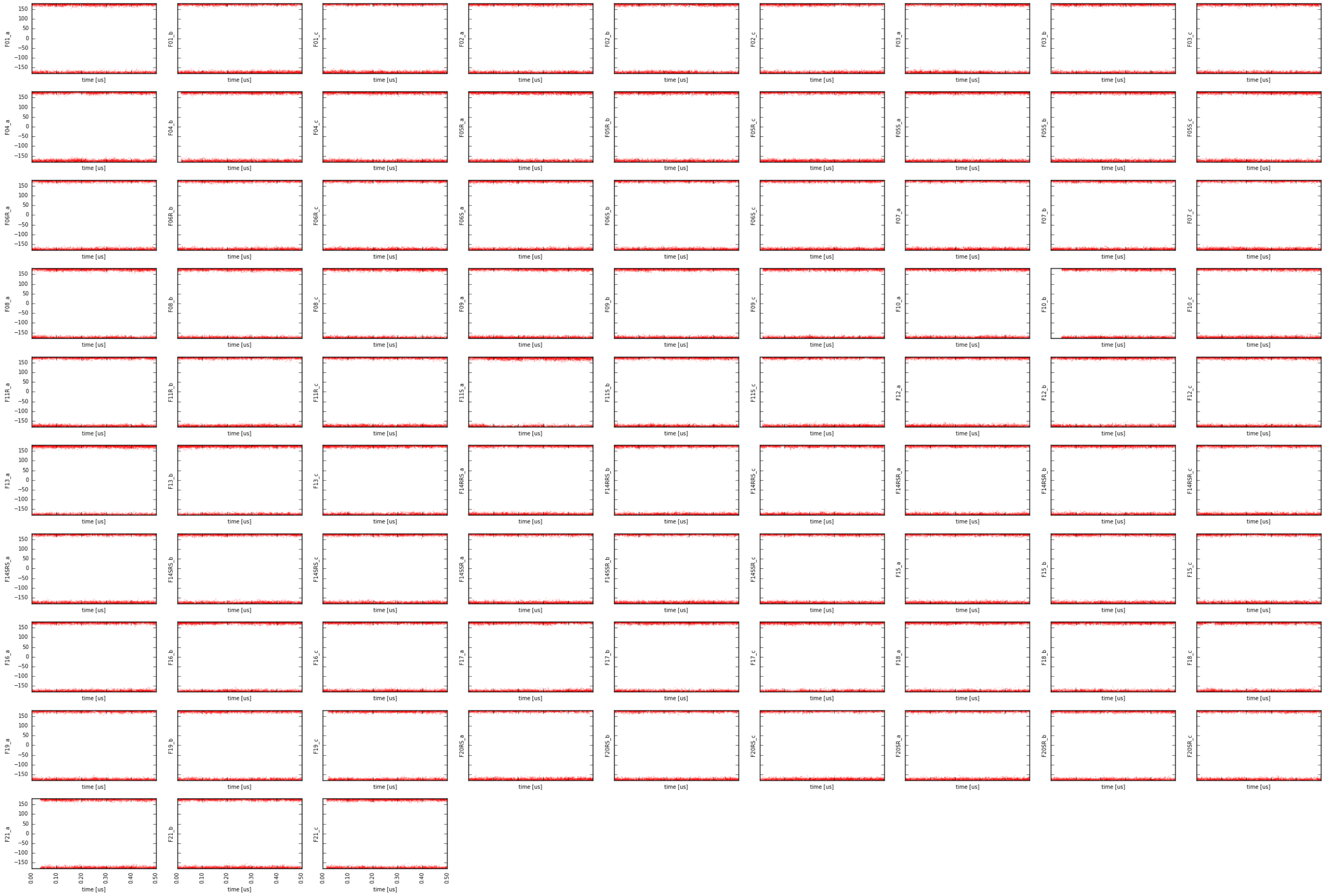


**Figure SM-DIH-34.** Time evolution of X1 dihedral angle of W133.

*Y-values are given in degrees.*

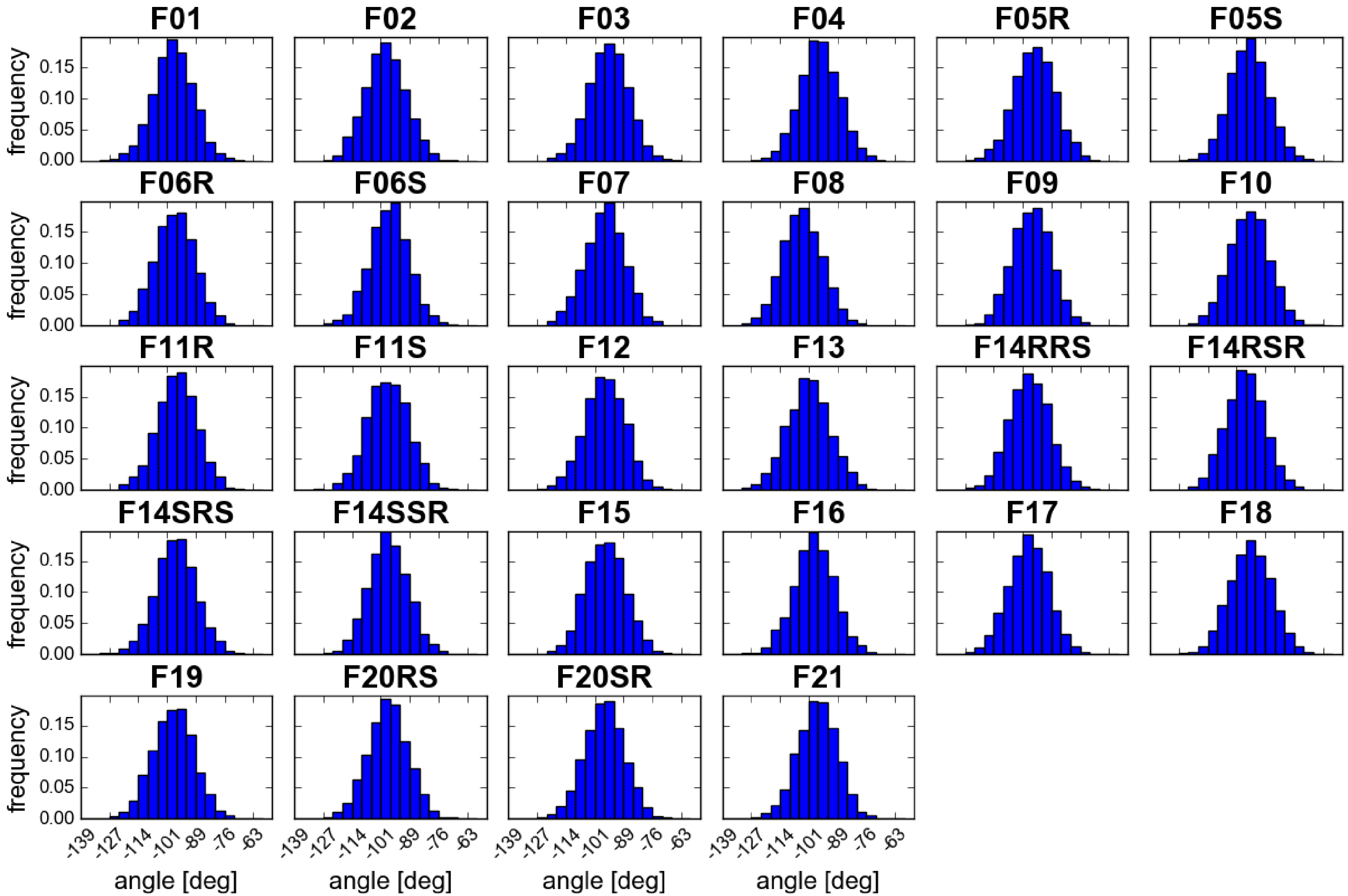


**Figure SM-DIH-35.** Distributions of X2 dihedral angle values of W133.
*Data are collected from last 10ns of production in 3 replicas.*

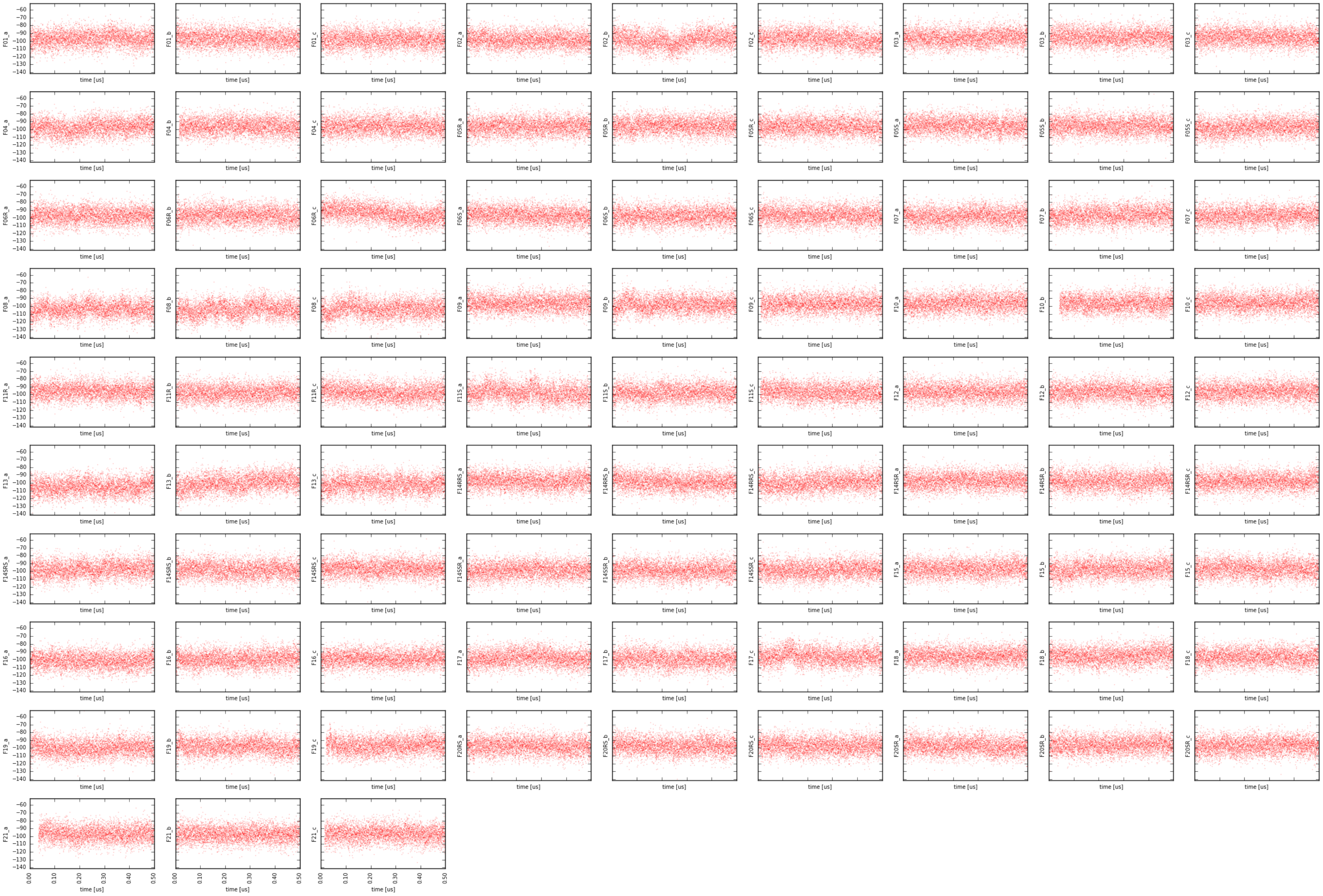

**Figure SM-DIH-36.** Time evolution of X2 dihedral angle of W133.

*Y-values are given in degrees.*

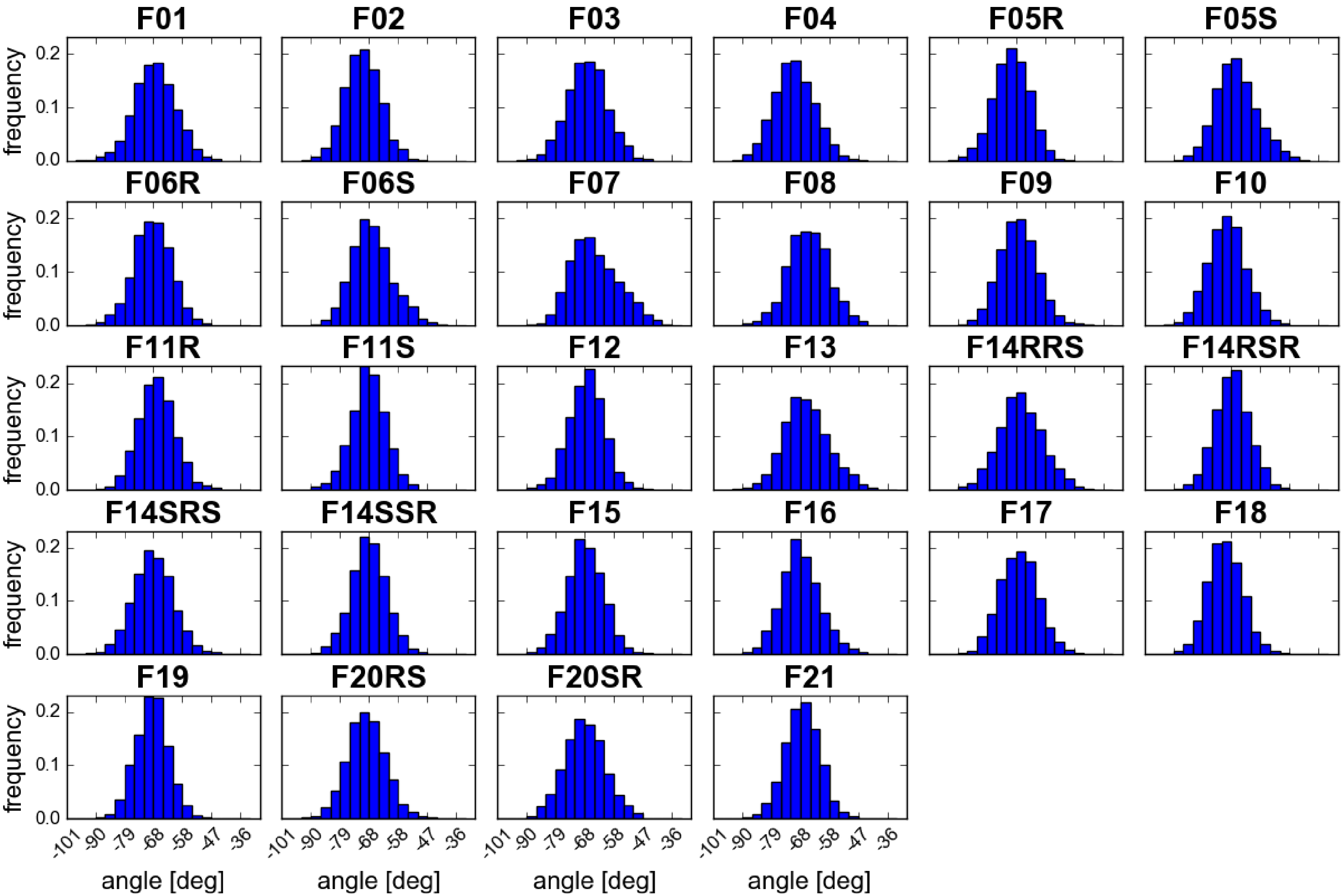


**Figure SM-DIH-37.** Distributions of X1 dihedral angle values of Y336.
*Data are collected from last 10ns of production in 3 replicas.*

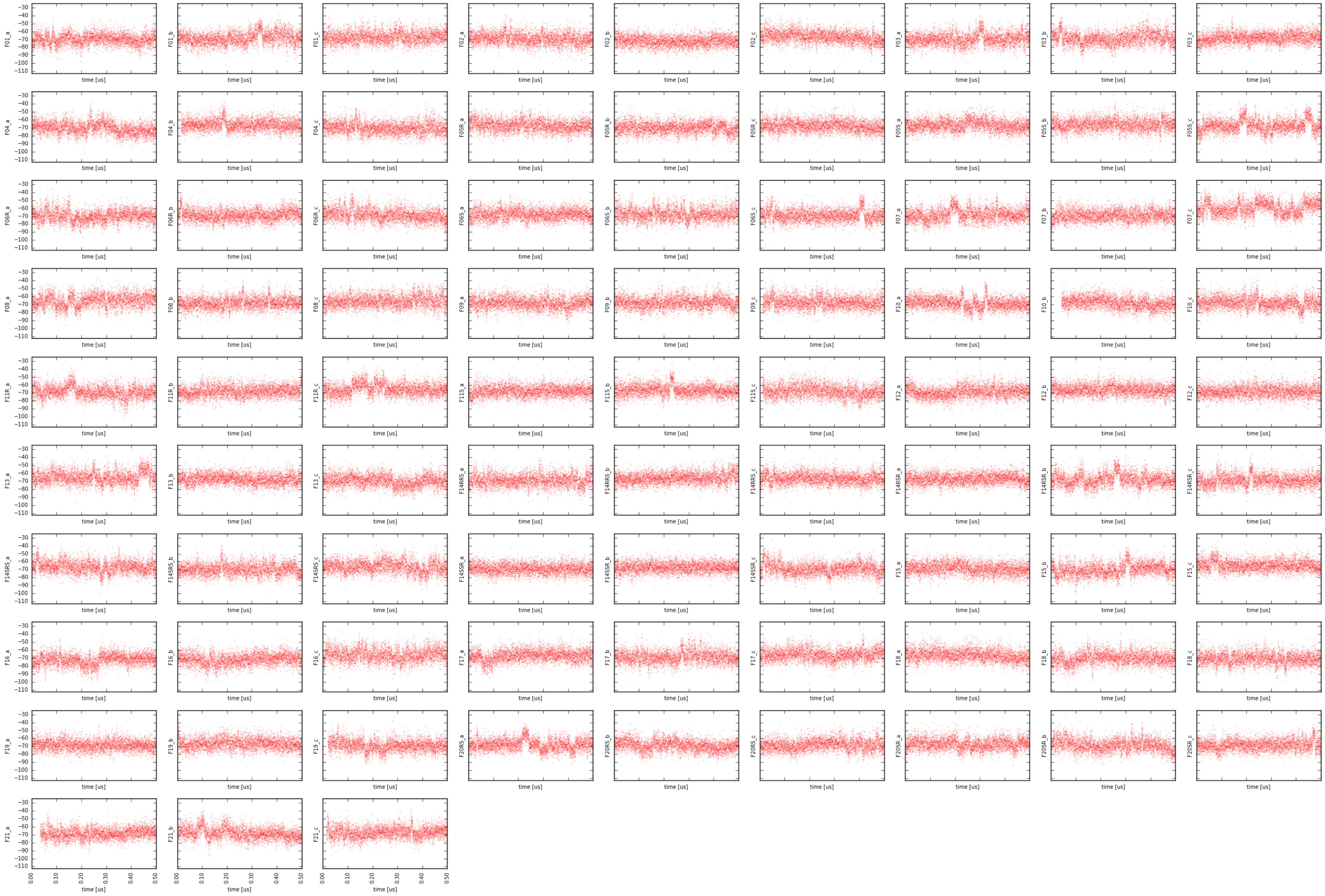

**Figure SM-DIH-38.** Time evolution of X1 dihedral angle of Y336.
*Y-values are given in degrees.*

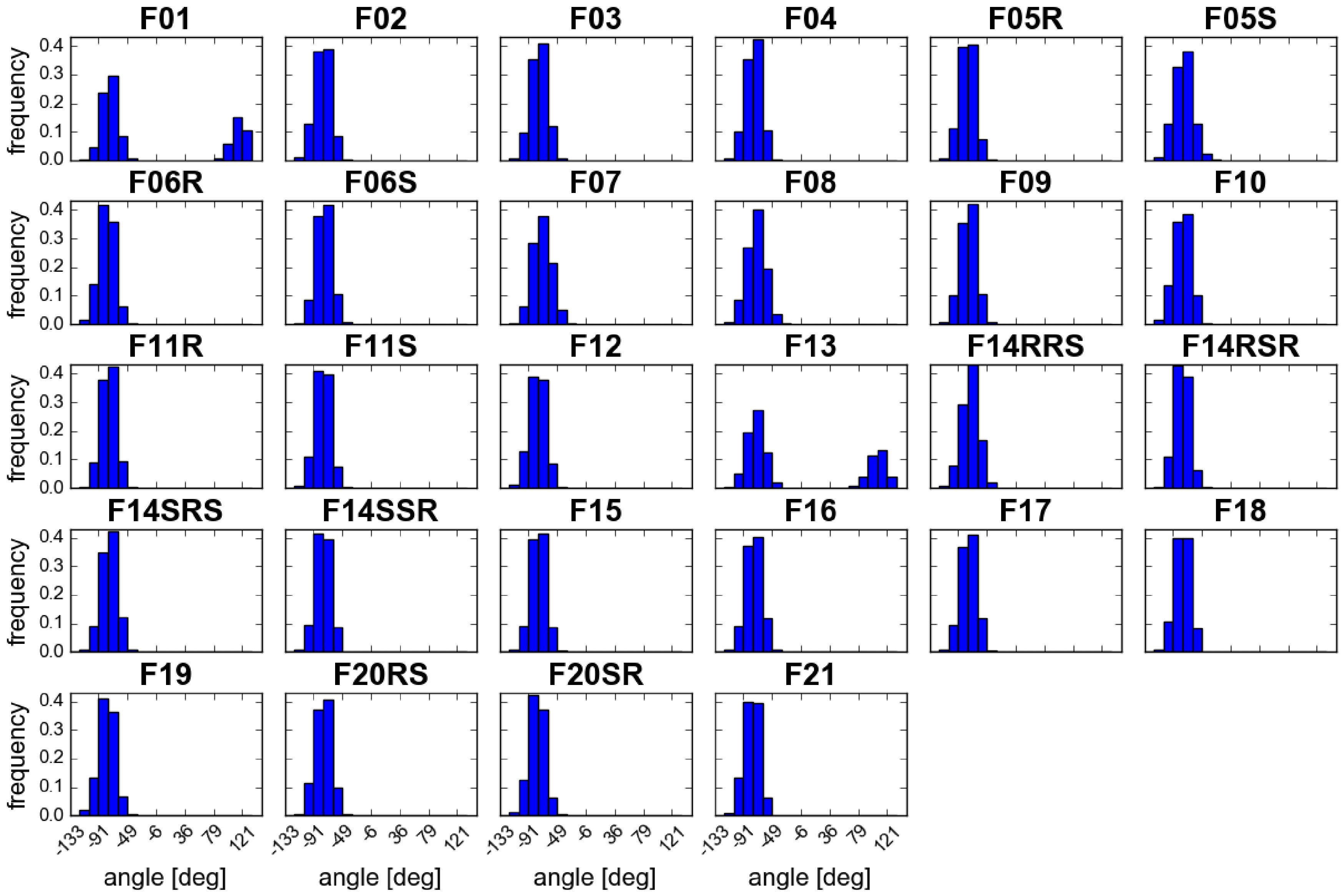


**Figure SM-DIH-39.** Distributions of X2 dihedral angle values of Y336.
*Data are collected from last 10ns of production in 3 replicas.*

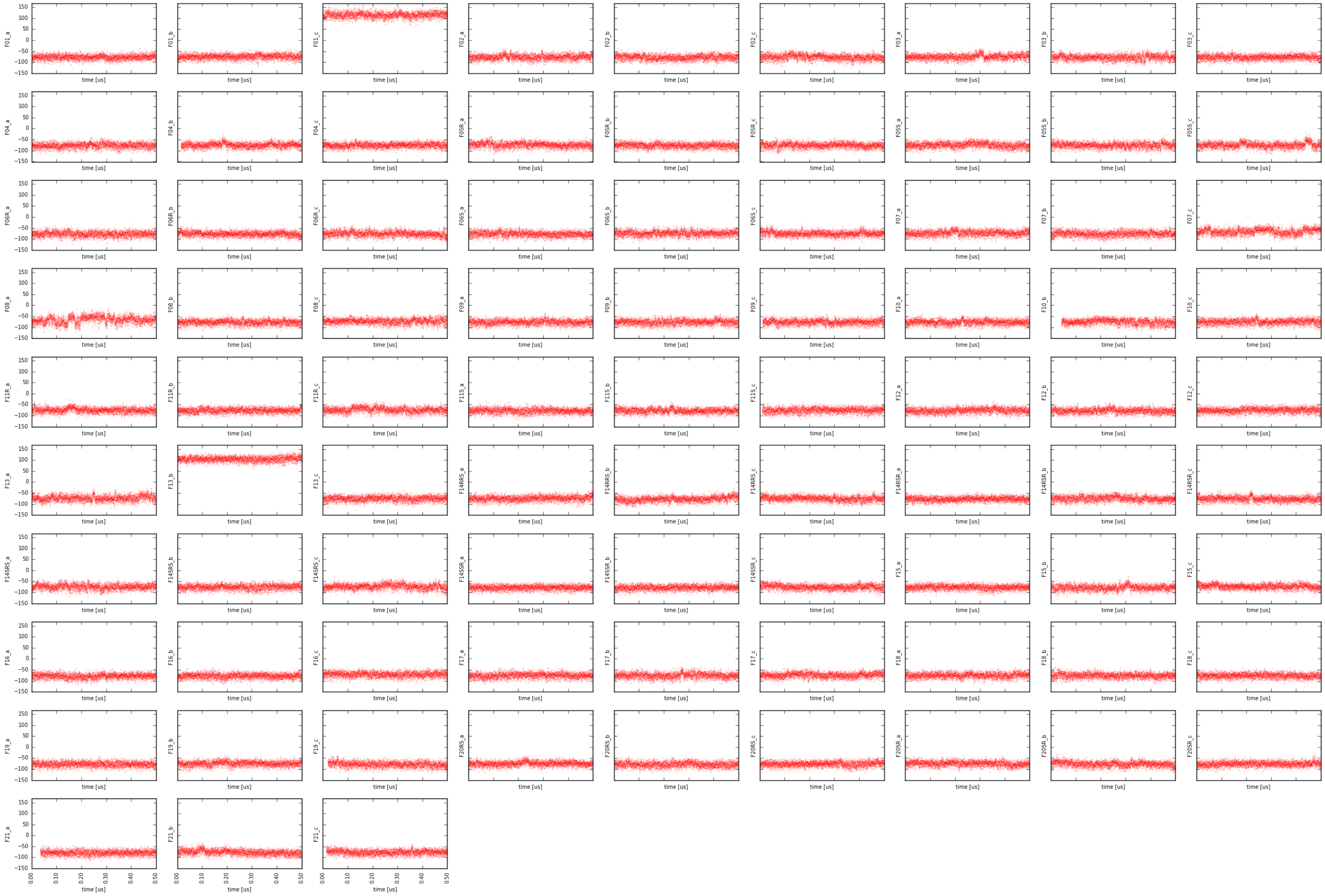

**Figure SM-DIH-40.** Time evolution of X2 dihedral angle of Y336.
*Y-values are given in degrees.*

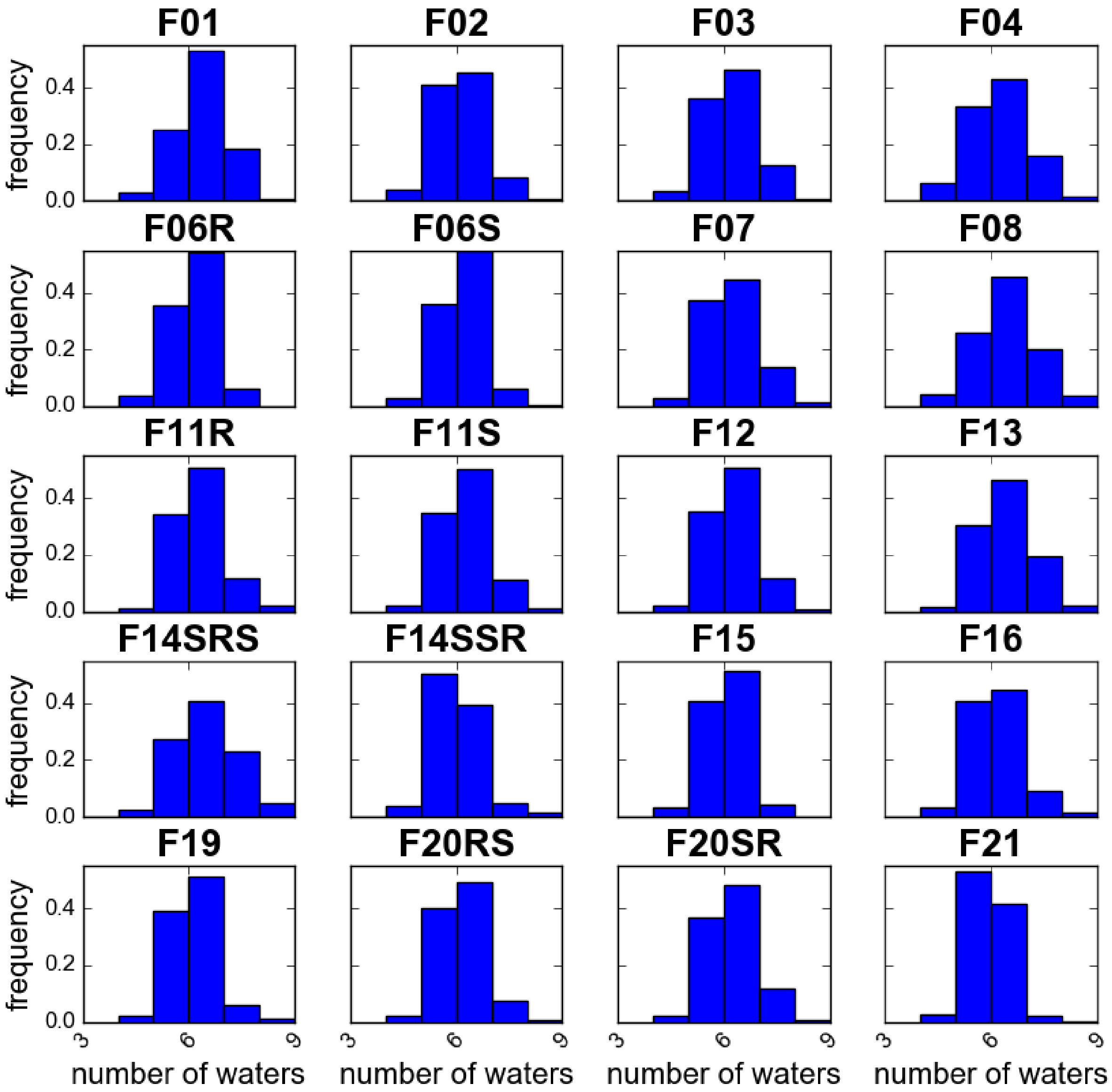


**Figure SM-WAT-1.** Distributions of the number of water molecules within 5.0 angstroms from Y336
*Data are collected from last 10ns of production in 3 replicas.*

**Table SM-WAT-1.** Mean number of water molecules within 5.0 angstroms from D114.
*Calculated on last 10 ns of simulations in 3 replicas.*

| derivative | replica | | | | | | | |
|---|---|---|---|---|---|---|---|---|
| | a | | b | | c | | | |
| | mean | sem | mean | sem | mean | sem | MEAN | SEM |
| F01 | 5.99 | 0.07 | 6.01 | 0.07 | 5.65 | 0.08 | 5.88 | 0.12 |
| F02 | 5.29 | 0.07 | 5.63 | 0.06 | 5.85 | 0.07 | 5.59 | 0.16 |
| F03 | 5.97 | 0.06 | 5.68 | 0.06 | 5.49 | 0.04 | 5.71 | 0.14 |
| F04 | 5.99 | 0.06 | 5.62 | 0.05 | 5.58 | 0.06 | 5.73 | 0.13 |
| F05R | 5.59 | 0.07 | 5.62 | 0.07 | 5.66 | 0.07 | 5.62 | 0.02 |
| F05S | 5.71 | 0.07 | 5.87 | 0.08 | 5.68 | 0.07 | 5.75 | 0.06 |
| F06R | 5.64 | 0.06 | 5.69 | 0.07 | 5.57 | 0.07 | 5.63 | 0.03 |
| F06S | 5.59 | 0.07 | 5.59 | 0.06 | 5.82 | 0.07 | 5.67 | 0.08 |
| F07 | 5.47 | 0.07 | 5.85 | 0.08 | 5.91 | 0.07 | 5.74 | 0.14 |
| F08 | 6.00 | 0.10 | 5.82 | 0.07 | 5.94 | 0.09 | 5.92 | 0.05 |
| F09 | 5.52 | 0.06 | 5.59 | 0.05 | 5.96 | 0.06 | 5.69 | 0.14 |
| F10 | 5.78 | 0.05 | 6.26 | 0.06 | 6.13 | 0.05 | 6.06 | 0.14 |
| F11R | 5.86 | 0.09 | 5.79 | 0.07 | 5.73 | 0.07 | 5.79 | 0.04 |
| F11S | 5.63 | 0.06 | 5.44 | 0.06 | 6.16 | 0.08 | 5.74 | 0.22 |
| F12 | 5.77 | 0.07 | 5.60 | 0.06 | 5.85 | 0.08 | 5.74 | 0.07 |
| F13 | 5.72 | 0.07 | 6.39 | 0.08 | 5.60 | 0.06 | 5.90 | 0.25 |
| F14RRS | 6.36 | 0.09 | 5.75 | 0.08 | 5.59 | 0.07 | 5.90 | 0.23 |
| F14RSR | 5.79 | 0.08 | 5.69 | 0.09 | 5.69 | 0.06 | 5.72 | 0.03 |
| F14SRS | 5.78 | 0.09 | 5.99 | 0.08 | 6.33 | 0.10 | 6.03 | 0.16 |
| F14SSR | 5.42 | 0.06 | 5.34 | 0.07 | 5.72 | 0.08 | 5.49 | 0.12 |
| F15 | 5.43 | 0.07 | 5.63 | 0.05 | 5.62 | 0.06 | 5.56 | 0.07 |
| F16 | 5.36 | 0.06 | 5.53 | 0.07 | 6.08 | 0.08 | 5.66 | 0.22 |
| F17 | 5.51 | 0.07 | 5.61 | 0.07 | 5.83 | 0.09 | 5.65 | 0.09 |
| F18 | 5.75 | 0.07 | 5.90 | 0.08 | 5.55 | 0.08 | 5.73 | 0.10 |
| F19 | 5.65 | 0.04 | 5.72 | 0.06 | 5.61 | 0.05 | 5.66 | 0.03 |
| F20RS | 5.48 | 0.05 | 5.54 | 0.06 | 5.94 | 0.08 | 5.65 | 0.14 |
| F20SR | 5.73 | 0.07 | 5.68 | 0.07 | 5.74 | 0.07 | 5.72 | 0.02 |
| F21 | 5.17 | 0.03 | 5.49 | 0.04 | 5.69 | 0.04 | 5.45 | 0.15 |
| | | | | | | MEAN | 5.73 | |

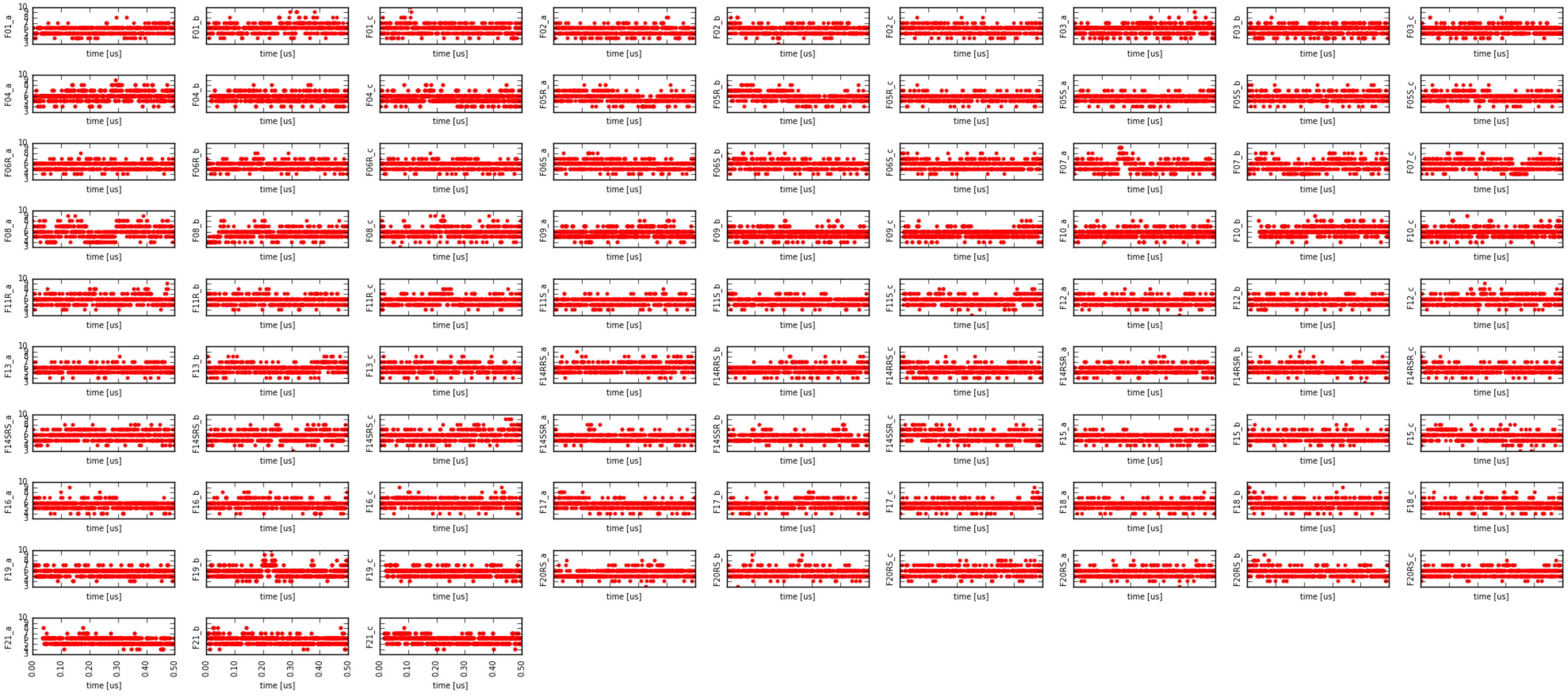

**Figure SM-WAT-2.** Number of waters in proximity (5.0 angstroms) of D114 over time.
*Y-values are unitless.*

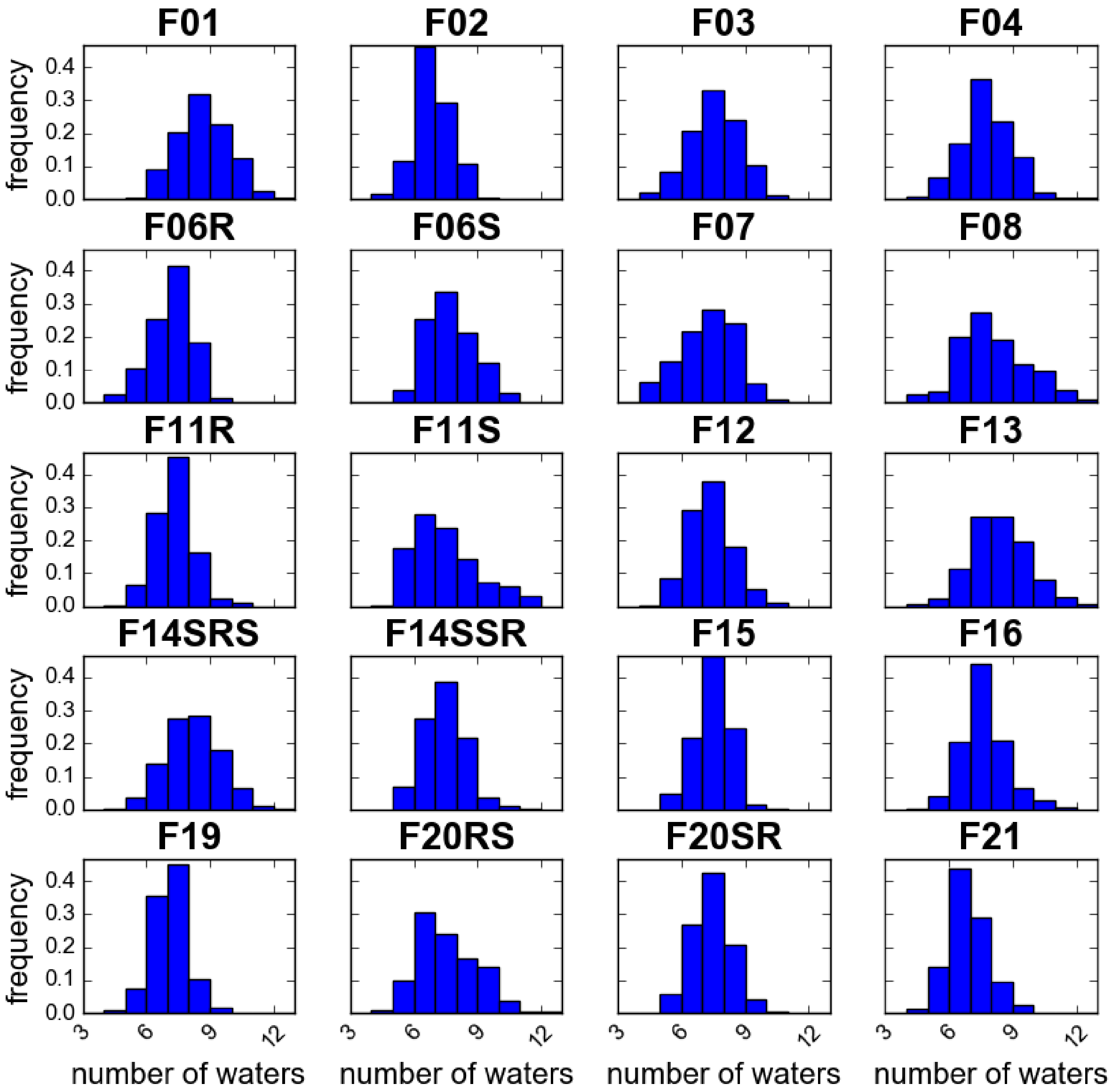


**Figure SM-WAT-3.** Distributions of the number of water molecules within 5.0 angstroms from Y336
*Data are collected from last 10ns of production in 3 replicas.*

**Table SM-WAT-2.** Mean number of water molecules within 5.0 angstroms from Y336.
*Calculated on last 10 ns of simulations in 3 replicas.*

| derivative | replica | | | | | | | |
|---|---|---|---|---|---|---|---|---|
| | a | | b | | c | | | |
| | mean | sem | mean | sem | mean | sem | MEAN | SEM |
| F01 | 8.35 | 0.12 | 7.39 | 0.10 | 8.79 | 0.12 | 8.18 | 0.41 |
| F02 | 6.69 | 0.09 | 6.01 | 0.08 | 6.41 | 0.08 | 6.37 | 0.20 |
| F03 | 7.60 | 0.08 | 7.48 | 0.07 | 6.05 | 0.07 | 7.04 | 0.50 |
| F04 | 7.09 | 0.07 | 6.87 | 0.08 | 7.79 | 0.10 | 7.25 | 0.28 |
| F05R | 6.89 | 0.08 | 5.93 | 0.09 | 6.57 | 0.09 | 6.46 | 0.28 |
| F05S | 7.41 | 0.10 | 6.69 | 0.09 | 6.19 | 0.12 | 6.76 | 0.35 |
| F06R | 6.74 | 0.08 | 6.52 | 0.13 | 6.70 | 0.09 | 6.65 | 0.07 |
| F06S | 6.47 | 0.06 | 8.05 | 0.12 | 7.10 | 0.12 | 7.21 | 0.46 |
| F07 | 7.13 | 0.09 | 7.40 | 0.12 | 5.61 | 0.12 | 6.71 | 0.56 |
| F08 | 8.03 | 0.14 | 6.57 | 0.10 | 8.27 | 0.21 | 7.62 | 0.53 |
| F09 | 7.07 | 0.06 | 7.36 | 0.08 | 6.99 | 0.08 | 7.14 | 0.11 |
| F10 | 6.79 | 0.05 | 9.20 | 0.07 | 7.38 | 0.07 | 7.79 | 0.73 |
| F11R | 6.86 | 0.08 | 6.52 | 0.08 | 7.07 | 0.11 | 6.82 | 0.16 |
| F11S | 5.71 | 0.07 | 6.58 | 0.09 | 8.57 | 0.13 | 6.95 | 0.85 |
| F12 | 6.57 | 0.10 | 6.57 | 0.09 | 7.41 | 0.11 | 6.85 | 0.28 |
| F13 | 7.63 | 0.15 | 8.92 | 0.11 | 7.09 | 0.08 | 7.88 | 0.54 |
| F14RRS | 7.62 | 0.16 | 7.36 | 0.11 | 7.47 | 0.08 | 7.48 | 0.08 |
| F14RSR | 6.05 | 0.09 | 6.98 | 0.07 | 6.70 | 0.10 | 6.58 | 0.28 |
| F14SRS | 7.26 | 0.12 | 7.92 | 0.11 | 7.87 | 0.15 | 7.68 | 0.21 |
| F14SSR | 6.68 | 0.09 | 6.66 | 0.10 | 7.43 | 0.11 | 6.92 | 0.25 |
| F15 | 7.14 | 0.08 | 6.62 | 0.08 | 7.17 | 0.09 | 6.98 | 0.18 |
| F16 | 6.87 | 0.06 | 6.67 | 0.09 | 7.98 | 0.13 | 7.17 | 0.41 |
| F17 | 7.42 | 0.10 | 6.48 | 0.09 | 7.09 | 0.12 | 7.00 | 0.28 |
| F18 | 6.66 | 0.12 | 7.84 | 0.08 | 7.55 | 0.09 | 7.35 | 0.36 |
| F19 | 6.85 | 0.05 | 6.60 | 0.06 | 6.42 | 0.06 | 6.62 | 0.12 |
| F20RS | 6.54 | 0.09 | 6.10 | 0.07 | 8.58 | 0.10 | 7.07 | 0.76 |
| F20SR | 6.82 | 0.10 | 6.79 | 0.07 | 7.11 | 0.10 | 6.91 | 0.10 |
| F21 | 6.36 | 0.05 | 5.99 | 0.06 | 6.84 | 0.08 | 6.40 | 0.25 |
| | | | | | | MEAN | 7.07 | |

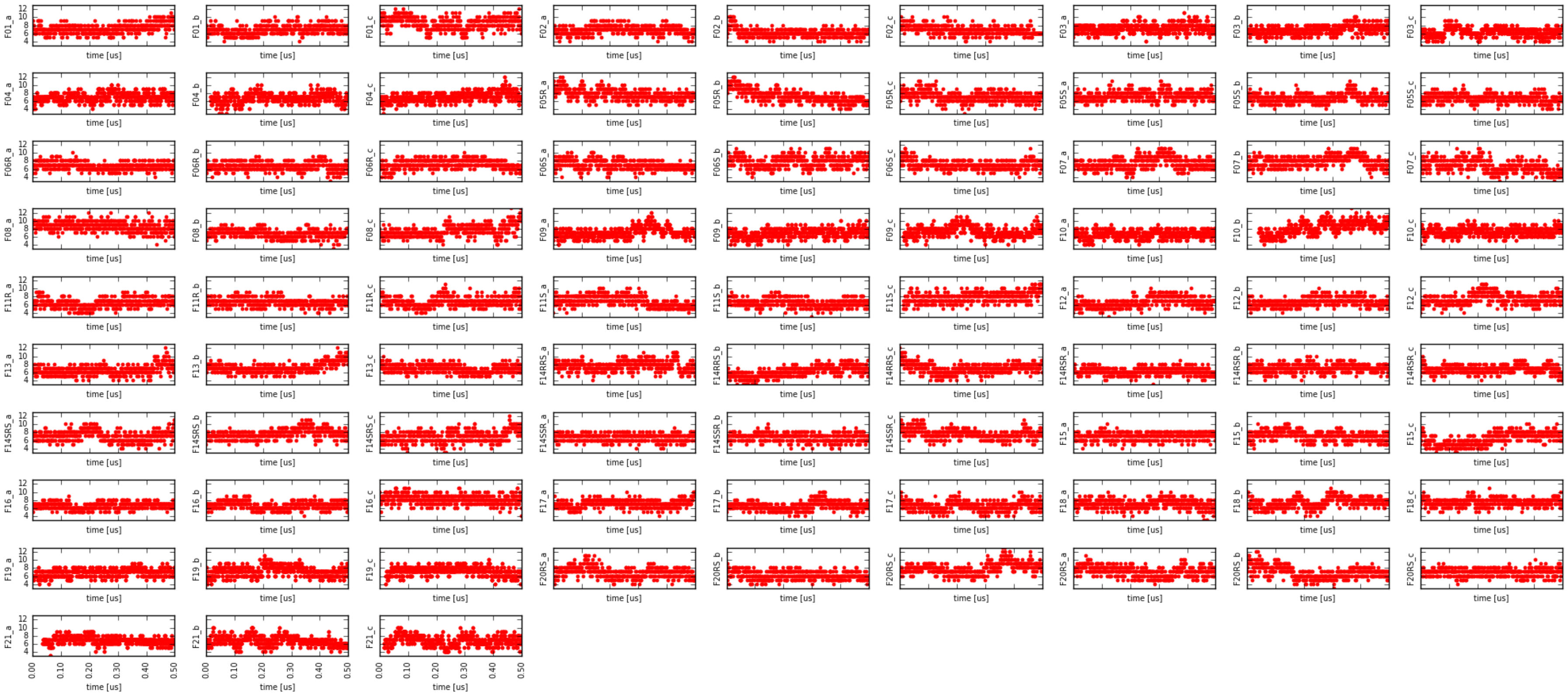


**Figure SM-WAT-4.** Number of waters in proximity (5.0 angstroms) of Y336 over time.
*Y-values are unitless.*

**Table SM-SCO-1.** Optimal scoring values for the tested derivatives.

*By optimal is meant either minimum or maximum (depending on the scoring function) scoring made on a set of snapshots from last 10 ns of production of 3 replicas of MD simulations.*

| derivative | PLP1 | PLP2 | PMF | PMF04 | DOCK_CS | DOCK_GS | Jain | LigScore1 | LigScore2 | Ludi1 | Ludi2 | Ludi3 | DSX | Ludi3_LIPSCO | VINA | VINA_repulsion |
|---|---|---|---|---|---|---|---|---|---|---|---|---|---|---|---|---|
| **F01** | 87.55 | 85.07 | 118.45 | 67.57 | -55.27 | -51.19 | 6.19 | 4.17 | 5.93 | 674.00 | 582.00 | 788.00 | -136.70 | 436.00 | -8.46 | 3.79 |
| **F02** | 78.59 | 75.72 | 107.63 | 58.75 | -51.49 | -48.25 | 5.40 | 3.56 | 5.75 | 630.00 | 553.00 | 707.00 | -121.41 | 380.00 | -6.71 | 4.04 |
| **F03** | 76.44 | 74.70 | 104.52 | 61.13 | -48.45 | -44.30 | 5.04 | 3.80 | 5.30 | 611.00 | 533.00 | 676.00 | -106.81 | 380.00 | -7.35 | 3.28 |
| **F04** | 70.01 | 72.16 | 100.53 | 61.79 | -47.30 | -44.49 | 4.72 | 3.51 | 5.30 | 590.00 | 527.00 | 661.00 | -113.12 | 376.00 | -6.74 | 4.40 |
| **F05R** | 85.46 | 83.80 | 123.25 | 71.54 | -52.80 | -49.76 | 6.36 | 4.31 | 5.87 | 708.00 | 590.00 | 797.00 | -123.17 | 418.00 | -8.11 | 4.17 |
| **F05S** | 86.84 | 82.80 | 113.00 | 65.81 | -50.73 | -45.35 | 6.25 | 3.96 | 5.65 | 663.00 | 582.00 | 735.00 | -121.14 | 418.00 | -7.85 | 3.57 |
| **F06R** | 87.55 | 87.62 | 119.70 | 65.25 | -51.14 | -48.81 | 5.48 | 4.10 | 5.80 | 657.00 | 547.00 | 719.00 | -122.06 | 391.00 | -7.91 | 4.76 |
| **F06S** | 88.19 | 85.01 | 118.93 | 69.25 | -52.71 | -48.58 | 5.90 | 5.25 | 6.28 | 716.00 | 598.00 | 752.00 | -124.12 | 395.00 | -8.01 | 3.73 |
| **F07** | 91.01 | 85.04 | 127.35 | 70.01 | -56.92 | -52.85 | 6.57 | 4.78 | 6.26 | 713.00 | 596.00 | 667.00 | -133.66 | 440.00 | -7.62 | 4.46 |
| **F08** | 100.67 | 99.32 | 133.51 | 89.36 | -61.80 | -58.86 | 6.28 | 5.38 | 6.57 | 678.00 | 594.00 | 698.00 | -149.91 | 470.00 | -8.75 | 5.51 |
| **F09** | 84.36 | 81.95 | 108.91 | 57.84 | -49.85 | -46.65 | 6.60 | 3.69 | 5.76 | 692.00 | 575.00 | 745.00 | -119.41 | 409.00 | -7.96 | 3.45 |
| **F10** | 85.54 | 83.94 | 118.11 | 63.90 | -51.88 | -47.97 | 5.95 | 4.81 | 6.12 | 717.00 | 571.00 | 743.00 | -121.93 | 359.00 | -7.44 | 3.65 |
| **F11R** | 84.41 | 85.32 | 127.00 | 73.72 | -52.42 | -49.84 | 6.76 | 5.30 | 6.19 | 735.00 | 599.00 | 753.00 | -122.24 | 378.00 | -7.91 | 3.17 |
| **F11S** | 77.03 | 76.05 | 119.19 | 64.74 | -51.07 | -46.34 | 5.05 | 4.35 | 5.90 | 705.00 | 585.00 | 804.00 | -117.94 | 351.00 | -7.50 | 4.05 |
| **F12** | 89.05 | 84.85 | 111.94 | 70.53 | -51.72 | -47.80 | 5.81 | 4.60 | 6.04 | 770.00 | 658.00 | 822.00 | -123.73 | 403.00 | -7.87 | 3.31 |
| **F13** | 83.58 | 80.32 | 119.59 | 80.36 | -54.06 | -50.56 | 5.37 | 4.47 | 5.85 | 676.00 | 594.00 | 693.00 | -135.31 | 403.00 | -8.06 | 4.22 |
| **F14RRS** | 81.78 | 78.27 | 117.29 | 58.53 | -48.24 | -45.50 | 3.05 | 3.04 | 5.41 | 482.00 | 441.00 | 636.00 | -115.19 | 397.00 | -7.67 | 2.23 |
| **F14RSR** | 80.19 | 76.44 | 110.32 | 60.92 | -52.50 | -49.42 | 5.19 | 3.59 | 5.77 | 595.00 | 523.00 | 739.00 | -115.46 | 388.00 | -7.89 | 4.11 |
| **F14SRS** | 82.43 | 79.40 | 106.37 | 60.25 | -48.24 | -46.17 | 3.28 | 3.25 | 5.65 | 532.00 | 465.00 | 684.00 | -109.51 | 384.00 | -7.47 | 3.04 |
| **F14SSR** | 87.65 | 83.99 | 128.24 | 68.24 | -52.65 | -47.74 | 6.08 | 4.21 | 5.98 | 670.00 | 554.00 | 727.00 | -118.05 | 386.00 | -7.62 | 4.67 |
| **F15** | 84.74 | 81.33 | 128.65 | 69.46 | -52.18 | -50.00 | 5.75 | 3.42 | 5.95 | 683.00 | 584.00 | 768.00 | -122.04 | 422.00 | -7.29 | 3.43 |
| **F16** | 86.19 | 81.39 | 117.95 | 64.54 | -54.85 | -52.03 | 4.28 | 4.03 | 6.00 | 648.00 | 554.00 | 778.00 | -124.85 | 434.00 | -7.75 | 3.96 |
| **F17** | 83.06 | 79.00 | 122.88 | 68.68 | -52.81 | -49.17 | 5.67 | 5.32 | 5.80 | 560.00 | 507.00 | 558.00 | -118.92 | 393.00 | -6.43 | 3.59 |
| **F18** | 61.11 | 62.12 | 83.26 | 52.10 | -40.33 | -38.30 | 4.51 | 3.76 | 5.10 | 557.00 | 481.00 | 485.00 | -83.83 | 295.00 | -5.74 | 3.06 |
| **F19** | 82.85 | 73.27 | 118.19 | 70.02 | -54.73 | -51.51 | 4.79 | 4.68 | 5.98 | 602.00 | 528.00 | 634.00 | -112.87 | 393.00 | -7.16 | 3.94 |
| **F20RS** | 80.05 | 77.46 | 101.51 | 60.38 | -49.46 | -46.44 | 3.16 | 2.22 | 5.42 | 491.00 | 462.00 | 544.00 | -121.97 | 409.00 | -6.89 | 3.00 |
| **F20SR** | 78.58 | 76.51 | 99.61 | 63.33 | -49.47 | -46.19 | 5.97 | 3.31 | 5.62 | 591.00 | 525.00 | 719.00 | -108.94 | 397.00 | -6.71 | 4.38 |
| **F21** | 81.37 | 77.56 | 113.83 | 68.62 | -52.65 | -49.48 | 5.67 | 4.41 | 6.16 | 678.00 | 590.00 | 737.00 | -114.95 | 424.00 | -6.73 | 4.12 |

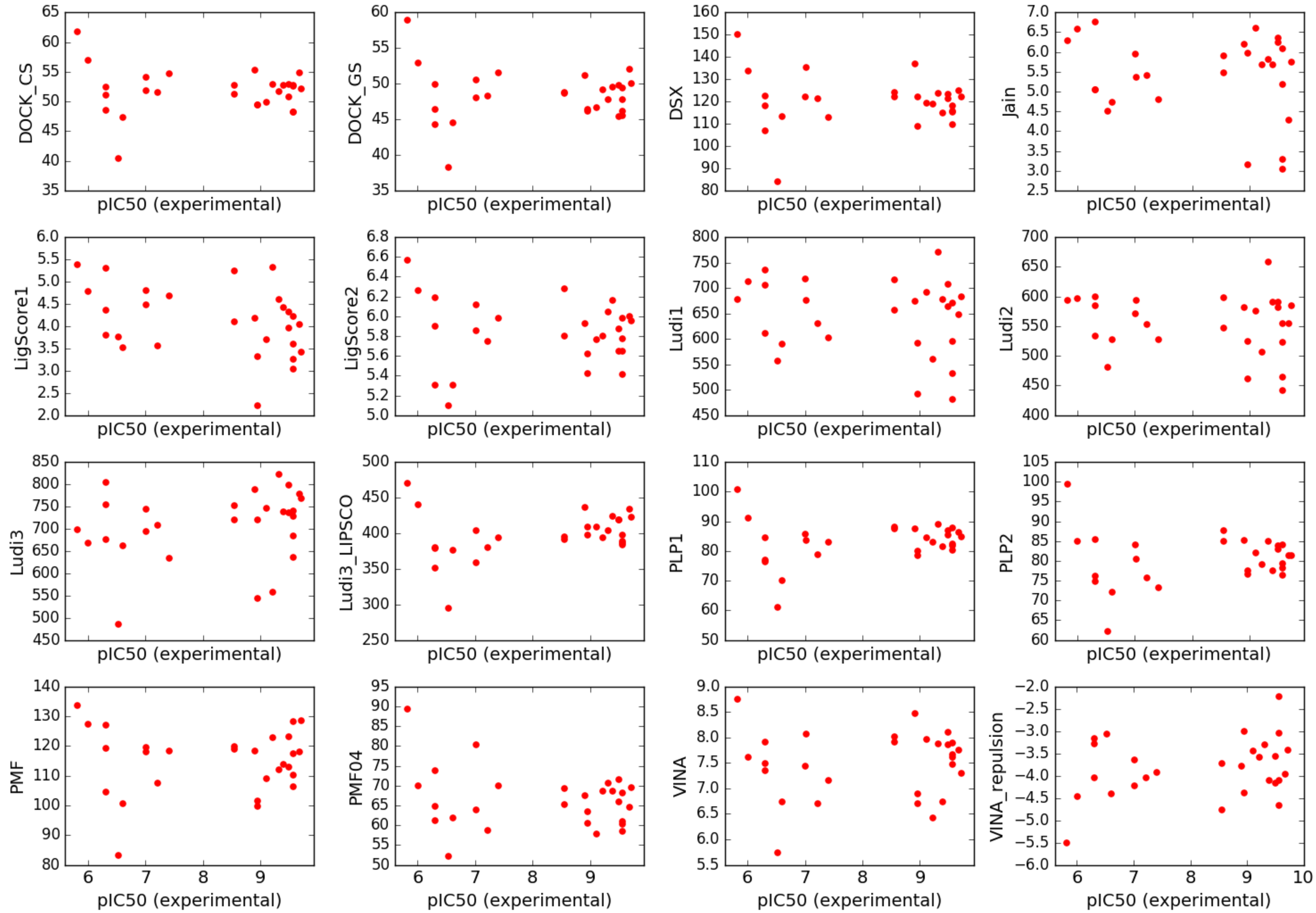


**Figure SM-SCO-1.** Plots of binding estimation by scoring functions (arbitrary units) against the experimental pIC50.
*The plot includes all tested derivatives. See Methods section for scoring functions labels.*

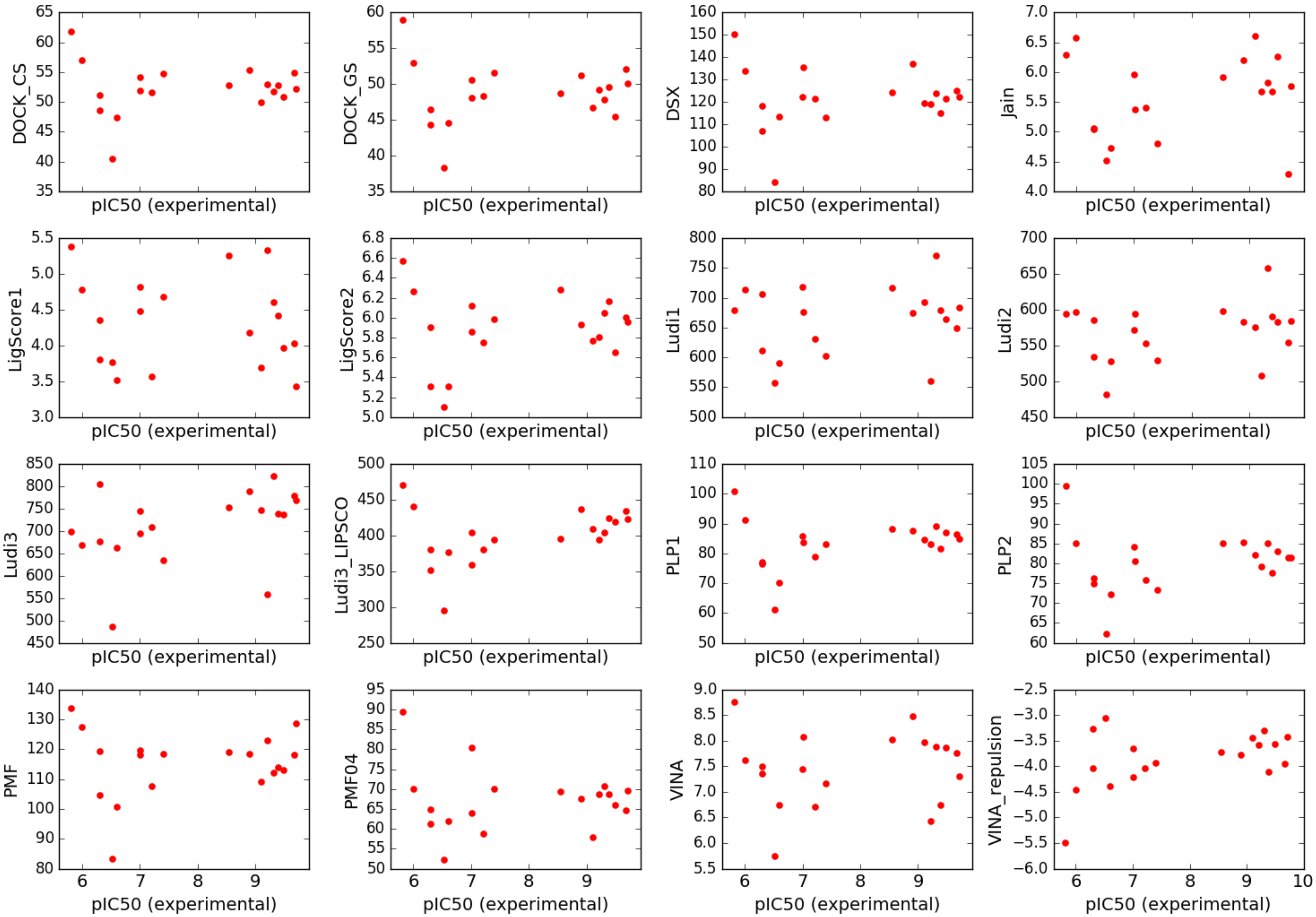


**Figure SM-SCO-2.** Plots of binding estimation by scoring functions (arbitrary units) against the experimental pIC50.
*The plot includes all tested derivatives except for the chiral compounds. See Methods section for scoring functions labels.*

$$
\begin{aligned}
pIC_{50} = \quad & -5.5527 \\
+ \quad & 0.00063 * \text{AROSCO (score for aromatic interactions)} \\
+ \quad & 0.00389 * \text{HBONDSCO (score for H-bonds)} \\
- \quad & 0.02543 * \text{IONSCO (score for ionic interactions)} \\
+ \quad & 0\ 04235 * \text{LIPSCO (score for lipophilic interactions)} \\
- \quad & 0.01135 * \text{ROTSCO (score for rotational freedom loss)}
\end{aligned}
$$

**Figure SM-SCO-3.** Expression for reweighted LUDI3 scoring function.
*This is internal validation on the set used for training the coefficients of the equation's elements.*

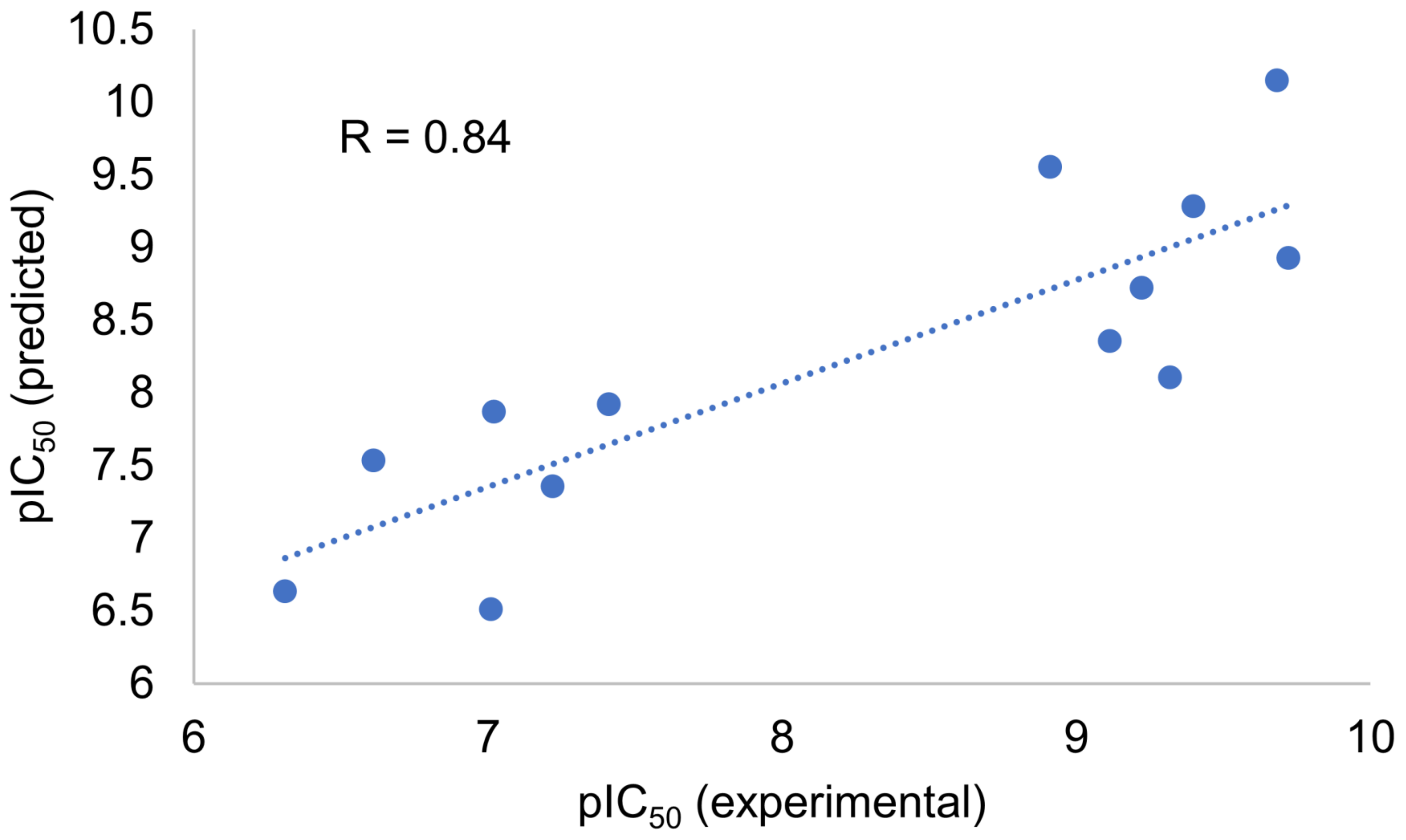


**Figure SM-SCO-4.** Plots of binding estimation by reweighted LUDI3 scoring function against the experimental pIC50. Internal validation. *Excluded are chiral compounds, F07, F08 and F18.*

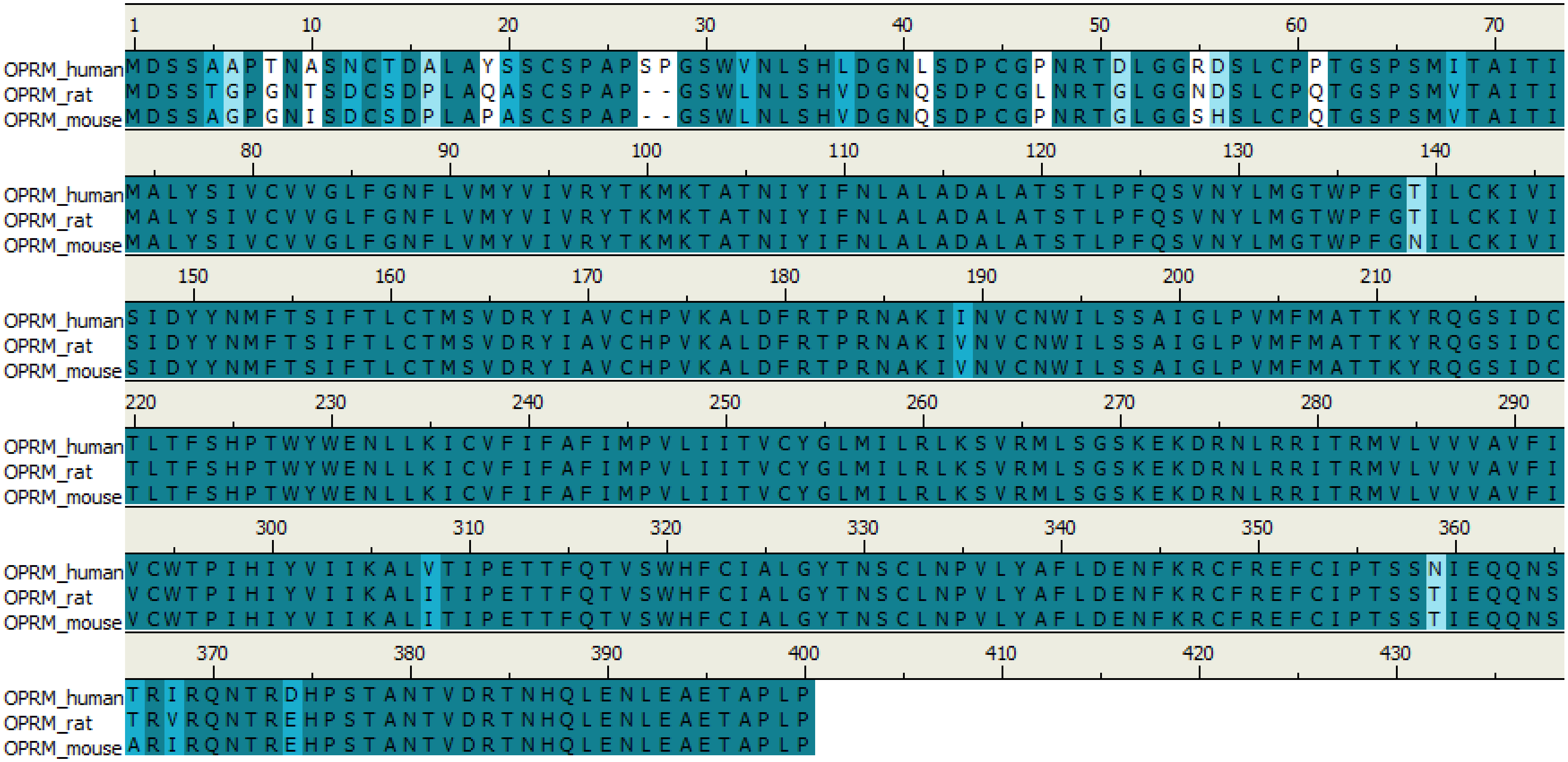

**Sequence SM-SEQ-1.** Sequence alignment of human, rat and murine µOR.

The interspecies differences in the sequences of µOR are of minor character and should not impact binding or scoring to a significant extent. Most of them are located in the N-terminus which is largely absent from 5C1M crystal (used for docking). Others, including 66:V:V:I (position: murine – rat – human), 137:N:T:T, 187:V:V:I, 306:I:I:V are 1) conservative 2) remote from the binding site or at least the side chain is not directed to the binding site. Three others are in the C-terminus, also absent from crystals, also remote from the binding site.
